# Order-to-Disorder and Disorder-to-Order Transitions of Proteins upon Binding to Phospholipid Membranes: Common Ground and Dissimilarities

**DOI:** 10.3390/biom15020198

**Published:** 2025-01-30

**Authors:** Reinhard Schweitzer-Stenner

**Affiliations:** Department of Chemistry, Drexel University, 3141 Chestnut Street, Philadelphia, PA 19104, USA; rs344@drexel.edu

**Keywords:** cytochrome *c*, α-synuclein, peripheral membrane proteins, electrostatic and hydrophobic binding, molecular crowding, α-synuclein self-assembly, anionic lipid membranes, Parkinson’s disease

## Abstract

Cytochrome *c* is one of the most prominent representatives of peripheral membrane proteins. Besides functioning as an electron transfer carrier in the mitochondrial respiratory chain, it can acquire peroxidase capability, promote the self-assembly of α-synuclein, and function as a scavenger of superoxide. An understanding of its function requires knowledge of how the protein interacts with the inner membrane of mitochondria. The first part of this article provides an overview of a variety of experiments that were aimed at exploring the details of cytochrome *c* binding to anionic lipid liposomes, which serve as a model system for the inner membrane. While cytochrome *c* binding involves a conformational change from a folded into a partially disordered state, α-synuclein is intrinsically disordered in solution and subjected to a partial coil -> helix transition on membranes. Depending on the solution conditions and the surface density of α-synuclein, the protein facilitates the self-assembly into oligomers and fibrils. As for cytochrome *c*, results of binding experiments are discussed. In addition, the article analyzes experiments that explored α-synuclein aggregation. Similarities and differences between cytochrome *c* and α-synuclein binding are highlighted. Finally, the article presents a brief account of the interplay between cytochrome *c* and α-synuclein and its biological relevance.

## 1. Introduction

A thorough understanding of the interplay between lipid membranes and proteins in biological cells is pivotal for gaining a picture of how biological cells work in their complex environment. Generally, one distinguishes between lipid interactions with proteins integrated into the membrane such as receptor proteins and interactions with so-called peripheral proteins, which, contrary to membrane proteins, are soluble in water. With regard to the latter, one differentiates between specific and unspecific binding. However, the boundary between both is somewhat blurred. The binding of many peripheral proteins to lipids frequently involves electrostatic interactions between positively charged residues on the protein surface and lipids with negatively charged head groups. In the case of, e.g., cytochrome *c* binding to the inner membrane of mitochondria, the rather special phospholipid cardiolipin, the head group of which carries two negative charges at physiological pH, seems to be the specific target [1]. The intrinsically disordered protein α-synuclein prefers cardiolipin and phosphatidylserine lipids [2]. Nonspecificity can be added to the binding process by hydrophobic interactions, which can lead to a stabilization of protein–lipid complexes. Folded proteins bind to membranes via specific binding domains, which might add some nonspecificity to the binding process. A list of prominent binding domains of peripheral membrane proteins can be found in the review of Monje-Galvan and Klauda [3]. The binding processes are generally reversible and involve solely non-covalent interactions. They are heavily influenced by the lipid membrane composition, the respective phase (crystalline, gel, or liquid-like), the presence of ions in solution and on the surface of biological cells, and by the presence of membrane proteins.

While general aspects of the binding of peripheral proteins to lipid membranes are understood in principle, a closer look at experimental results and theoretical analyses reveals a complex and intrinsically inconsistent picture. In this Perspective article, I delineate this complexity and the lack of thorough understanding for two classical peripheral binding proteins. The first one is the mitochondrial electron transport protein cytochrome *c*. This protein is fully folded in the solution, but several lines of evidence suggest that under certain conditions, it undergoes partial unfolding on the surface of cardiolipin-containing membranes [4,5,6,7]. The second one is α-synuclein. Contrary to cytochrome *c*, it is intrinsically disordered in solution, but partially adopts secondary structures on the surface of membranes with anionic lipids [8,9]. Hence, the two molecules represent two types of peripheral membrane-binding proteins with rather opposite structural properties. Interestingly, however, they do not always act isolated from each other [10,11]. As I will describe in more detail below cytochrome *c* might act as an agonist-type promoter of α-synuclein aggregation and fibrilization in the mitochondria of dopaminergic neurons.

**Figure 1:**
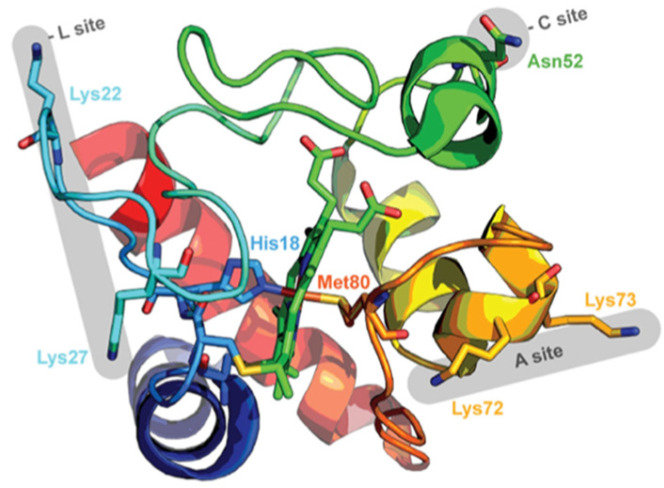
Solution structure of oxidized cytochrome *c* obtained from NMR studies (PDB 1AKK, Banci et al. [17]) with the proposed sites involved in the binding to membrane surfaces. The figure was taken from Soffer [18] with permission.

Mitochondrial cytochrome *c* is a classical heme protein with 104 amino acid residues. It belongs to class I of the family of cytochrome molecules. As shown in Figure 1, it is a globular protein with two major α-helices at the N- and C-terminals. Compared with, e.g., myoglobin, it contains significantly more loop sections (note that such segments that one finds in nearly all globular proteins should be distinguished from intrinsically disordered regions). The protein contains a heme *c* as prosthetic group, namely, an iron protoporphyrin IX group covalently attached to two cysteine residues via thioether bridges. The central metal atom is hexacoordinate to the four pyrrole nitrogens of the heme macrocycle, as well as to a nitrogen atom of the proximal histidine side chain (H18) and the sulfur atom of a methionine side chain (M80). The latter is mainly responsible for its high redox potential (0.254 V) [12]. The high pI value of the protein (9.6) indicates that the protein predominantly carries positive charges at physiological pH. Textbooks generally focus on its main function, namely, the shuttling of electrons from the CoQH2–cytochrome *c* reductase to the cytochrome *c* oxidase complex, which is an essential part of the energy-producing respiratory chain [13]. However, the protein is by no means monofunctional. Besides its electron transfer capability, its peroxidase activity is its most prominent function. It involves the binding to cardiolipin lipids in the outer leaflet of the inner membrane and its release from the inner to the extramitochondrial space, where it initiates a cascade that leads to mitochondrial apoptosis [4,5,14]. As already mentioned above and described in more detail in Section 5 of this article, it can promote α-synuclein assembly in the presence of H_2_O [10]. Moreover, it can function as a scavenger of superoxide, which is an important reactive oxygen species [15,16]. A full account of the multifunctionality of different cytochrome *c* species can be found in the review of Alvarez-Paggi et al. [5].

**Figure 2:**
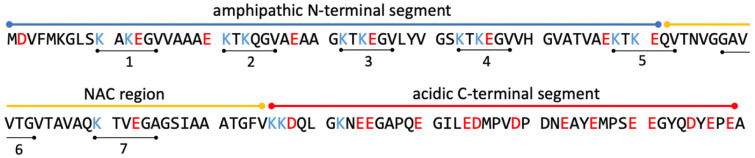
Amino acid residue composition and segment structure of α-synuclein. Residues with charge side chains at neutral pH are marked in color (red for negative and blue for positive charges). The numbers indicate the occurrence of the repeat motif. Taken from ref. [22] (open source).

α-Synuclein is a protein with 140 amino acid residues. Thus, where just size is concerned, it belongs more or less in the same class as cytochrome *c*. Contrary to the latter, it does not adopt a folded structure in solution. We call such proteins intrinsically disordered [19,20,21]. In spite of the absence of any secondary structure, the protein can be divided into three domains (Figure 2), i.e., the N-terminal domain (1–60), which carries a positive net charge, the so-called non-amyloid component (NAC, 61–95) domain, and finally the C-terminal domain with a negative net charge (96–140) [22]. The protein is prone to self-assembly into oligomers and amyloid fibrils, particularly on the surface of membranes with anionic lipids (*vide infra*). Since amyloid plaques of this protein have been found in neuronal intraplasmic Lewy bodies in the *substantia nigra* of Parkinson’s patients, it has been implicated as playing an important role in the development of this disease. The exact mechanism, however, it not yet known with certainty. The normal function of the protein has not been unambiguously identified either, but it is currently thought to play a role in the regulation of synaptic plasticity and neurotransmitter release [23,24,25]. However, it is known from a plethora of experimental data that α-synuclein binds to anionic lipids and that this binding process causes a partial disorder-to-order transition. Under certain circumstances to be described in Section 4, the accumulation of α-synuclein on lipid membranes causes oligomer and amyloid fibril formation. Interestingly, the protein can interact with (partially unfolded) cytochrome *c* in the cytosol during a phase of mitochondrial dysfunction in pesticide-related Parkinson’s diseases (Section 5).

As I show in this article, our current understanding of how these two proteins interact with lipid membranes is incomplete in spite of a multitude of experimental data. The article is aimed at on elucidating similarities and discrepancies between the physicochemical processes that govern the binding processes of both proteins. To this end, I will focus on addressing the following questions.

- To what extent do hydrophobic interactions contribute to the binding to lipid membranes in addition to the already mentioned electrostatic interactions?
- Do the proteins undergo a conformational transition upon their binding to lipid membranes and how does the probability for this to occur depend on the lipid-to-protein ratio?
- Do the above proteins penetrate the lipid membrane upon binding by at least partially moving into the interior of corresponding lipid vesicles?
- To what extent does the binding to lipid membranes change the function of the protein?

Regarding cytochrome *c*, I focus on how the protein might acquire peroxidase activity. With respect to α-synuclein, I discuss to what extent binding promotes oligomerization and fibril formation. As I show in this article, different experimental approaches yield rather different and sometimes contradictory answers to these questions.

The remainder of this article is organized as follows. In order to provide the necessary theoretical background for the understanding of this article, Section 2 starts with a short account of thermodynamic models used to describe the binding of proteins to negatively charged membrane surfaces. Section 3 deals entirely with the binding of cytochrome *c* to lipid vesicles of various sizes. Here, I discuss different types of binding experiments that have led to somewhat inconsistent results. Furthermore, I discuss structural studies of membrane-bound cytochrome c. Some of them argue in favor of conformational transitions, while others indicate a nearly native-like structure. I will briefly discuss to what extent cytochrome *c* binding can cause membrane fusion and peroxide activity. Section 4 deals with α-synuclein. Here, I will discuss and relate the role of electrostatics, membrane curvature, and membrane penetration. The rather specific conditions at which membrane-bound α-synuclein forms oligomers and/or amyloid fibrils is delineated. The interplay between cytochrome *c* and α-synuclein in the development of mitochondrial Parkinson’s disease (involving the functional loss of respective dopaminergic cells) is briefly outlined in Section 5. The article finishes with a summary and outlook (Section 6).

## 2. Thermodynamics of Protein Binding to Membrane Surfaces

### 2.1. Basic Electrostatic Theory

The simplest model frequently used to describe isotherms for the binding of proteins to membrane surfaces utilizes the Hill function:

(1) $$f_{pb}=\frac{{\left(K_{eff}\left[P\right]\right)}^{n}}{1+{\left(K_{eff}\left[P\right]\right)}^{n}}$$

where *K_eff_* is an effective binding constant, [*P*] is the free ligand concentration and *n* the Hill coefficient. Normally. Equation (1) is a convenient way of accounting for cooperative (*n* > 1) or anticooperative binding (*n* < 1). For *n* = 1, Equation (1) becomes a Langmuir isotherm. When applied to the binding of specific proteins to surfaces like lipid bilayers, *n* is generally interpreted as the number of (charged) lipids incorporated in a protein–lipid complex. If a sufficient number of protein-binding lipids are clustered prior to the binding (in the case of dominant fraction of such lipids or the occurring of lipid–lipid demixing), *n* is just a stoichiometric factor. However, if demixing occurs upon protein binding, *n* can in principle be understood as an indicator of cooperativity [26].

The above binding model is (too) simplistic because it assumes individual and independent binding sites. It does not explicitly consider the possibility that protein binding might be followed by conformational transitions and that in the limit of high occupation molecular crowding effects might affect the binding process. The possibility of membrane insertion is not taken into account either. The model does not specify individual contribution like electrostatic interactions, hydrogen bonding, or van der Waals interactions. In what follows below, I introduce the main elements of statistical mechanical approaches, which allow for a more detailed and differentiated mathematical account of protein binding to lipid membrane surfaces. The basics of the theoretical aspects sketched below have been developed by Heimburg, Marsh, Minton and their respective collaborators [27,28,29,30,31]. As shown in the subsequent section, they applied their theory to the binding of cytochrome *c* to anionic phospholipid membranes under various conditions. I add some aspects of a later approach of Kinnunen and coworkers [32] that utilized the Heimburg-Minton formalism for the interpretation of fluorescence energy transfer experiments. The text below just lists and discusses those equations that are most relevant for an understanding of the underlying concepts. Readers who are interested in the mathematical details are referred to the papers cited above. A detailed account of the theory can also be found in Heimburg’s textbook [33].

I start with the basic equation of the original Heimburg–Marsh theory [27]. It relates the binding isotherm to the Helmholtz energy change produced by the binding of a protein to membrane surfaces:

(2) $$\left\langle i\right\rangle =\left[P\right]K_{0}exp\left(-\frac{d}{di}\left[\frac{\Delta F\left(i\right)}{RT}\right]\right)$$

where *K*_0_ is an intrinsic binding constant and Δ*F(i)* is the change in the Helmholtz energy associated with the binding of *i* proteins. Note that Equation (2) uses energies in units of J/mol, while the equations in the original literature express energies in units of joule per molecule.

In order to arrive at a more explicit form for the binding isotherm, one has to derive a formalism for the Helmholtz energy. For a homogeneously charged surface, Heimburg and Marsh considered two contributions. The first one is called distributional free energy and is written as:

(3) $$\Delta F_{D,vdW}\left(i\right)=-iRTln\left(n-i\right)-E_{pp}RT\left(\frac{i^{2}}{n}\right)$$

The first term in Equation (3) reflects a Boltzmann-type contribution where the linear dependence on the number *i* of bound proteins is modified by a logarithmic term that reflects the decreasing entropy gain for *i→n*, where *n* is the total number of binding sites. The second term scales linearly with the interaction energy *E_pp_* between bound proteins. In other words, this term accounts for the influence of molecular crowding on the proteins’ aggregation or repulsion. In the formalism of Heimburg and Marsh, it is positive for attractive and negative for repulsive interactions, somewhat at variance with normal conventions. Equation (3) treats the surface protein ensemble as a two-dimensional van der Waals gas. Alternatively, one can derive a slightly different equation by using the scaled particle theory (SPT), which treats the surface proteins and lipids as hard-sphere fluids [34]:

(4) $$\Delta F_{D,SPT}\left(i\right)=-iRT\left[ln\left(n-i\right)-1\right]-RT\left(\frac{i\cdot n}{n-i}\right)$$

Apparently, interactions between proteins are now exclusively repulsive. The modification of the first term is rather modest and becomes relevant only when *i→n.* Note that the second term approaches ∞ in such a case. Hence, binding becomes improbable close to saturation even though there are still empty spots to fill. As a consequence, the SPT model overestimates the hard-disk pressure, while the van der Waals model underestimates the repulsive free energy.

For a homogeneously charged surface (e.g., a lipid membrane with 100% cardiolipin or phosphatidylglycerol), the electrostatic free energy difference between unbound and bound states of a protein reads as:

(5) $$\Delta F_{el}=F_{el,s}\left(i\right)-F_{el,s}\left(0\right)-{iF}_{el,P}$$

where the first, second, and third terms represent the electrostatic free energy of the membrane with *i* proteins bound to its surface, without any bound proteins, and for the unbound protein in solution, respectively. Regarding the high-potential limit, Jähnig showed that the first term can be written as [35]:

(6) $$F_{el}\left(i\right)=2\frac{q}{e}\cdot RT\cdot ln\left(\frac{-\Lambda_{0}\sigma }{\sqrt{I}}\right)$$

with:

(7) $$q=-\left(n\alpha f_{A}-iZ\right)$$

where *Z* is the number of charges on the protein and *α* denotes the number of lipids in contact with a single protein, *f_A_* is the fraction of homogeneously distributed negatively charged lipids, and *Z* the number of effective protein charges. The elementary charge is written as *e*. The charge density *σ* in the logarithmic term of Equation (6) is written as:

(8) $$\sigma =-\frac{\left(n\alpha f_{a}-iZ\right)e}{n\Delta A}$$

where Δ*A* is the area occupied by a single protein. It should be noted that the argument of the logarithmic function in Equation (6) has to be larger than 2 for the validity of the high potential approach. The factor *Λ*_0_ in Equation (6) is a temperature dependent factor

(9) $$\Lambda_{0}=\sqrt{\frac{\pi }{2\epsilon RT}}$$

where *ε* denotes the dielectric constant of the solution. One easily sees that Equation (8) reduces to the number of lipid surface charges for *i* = 0, which is the second term in Equation (4). As shown by Heimburg and Marsh, the third term in this equation can be approximated by:

(10) $$F_{el,p}={RTZ}^{2}\Lambda_{2}\left(1-1/2\cdot ln\left(\kappa r_{0}\right)\right)$$

where *κ* is the ionic strength-dependent reciprocal Debye length, *r*_0_ is the radius of a sphere representing the protein, and:

(11) $$\Lambda_{2}=\frac{N_{A}e^{2}}{4\pi \epsilon_{0}r_{0}RT}$$

with *N_A_* denoting the Avogadro number and *ε*_0_ the vacuum permeability.

It should be noted that the electrostatic part of Helmholtz energy describes binding as the potential difference between proteins in solution and on the surface of the lipid membrane. It does not explicitly contain a term that reflects the strength of electrostatic interactions between protein and lipid head group. This notion can be directly inferred from Equations (6) and (7). The latter term is just a measure of the net charge of the protein–lipid ensemble and approaches zero if the number of charged lipids involved in the binding processes becomes equal to the protein charge. The intrinsic binding energy solely contributes to the *K*_0_ constant in Equation (2). The ionic strength of the solution enters the formalism in Equation (6) and indirectly via the Debye length in Equation (10). As we will see in the subsequent section, even moderate ionic strengths in the centimolar region can reduce the effective binding constants of proteins effectively. In order to derive a binding isotherm that can be used to fit experimental data, one has to add up all the Helmholtz energy terms presented above and calculate the derivative in Equation (2). Details and the final formalism can be inferred from the paper of Heimburg and Marsh [27].

### 2.2. Redistribution of Anionic Lipids After Protein Binding

What happens if the presence of bound ligands causes a lipid redistribution and thus the clustering of anionic ligands at the binding site of proteins? Heimburg et al. treated this case as an equilibrium between the binding of proteins to two different lipids, termed A and B [29]. A relative binding constant that relates the binding to A to the binding of B can be written as:

(12) $$K_{r}=\frac{\left[PA\right]\left[B\right]}{\left[A\right]\left[PB\right]}$$

The Helmholtz energy *F_LD_* of the demixing–redistribution process is written as:

(13) $$\Delta F_{LD}\left(N_{\theta },N(A)\right)=-ln\left(\frac{\Omega_{N_{\theta }(A)}\left(N_{\theta },N(A)\right)}{\Omega_{N_{\theta }^{0}(A)}\left(N_{\theta },N(A)\right)}\right)\cdot K_{r}^{N_{0}(A)-N_{0}^{0}(A)}$$

where *N_θ_* is the total number of lipids bound to proteins, *N(A)* is the number of A lipids, and *N_θ_(A)* is the number of A sites associated with proteins. The subscript *θ* indicates that *N* depends on the degree of surface coverage. The degeneracy functions *Ω*(*N_θ_, N(A)*) can be calculated as follows:

(14) $$\Omega_{N_{\theta }(A)}=\left(\frac{N_{\theta }!}{\left(N_{\theta }-N_{\theta }\left(A\right)\right)!N_{\theta }(A)!}\right)\left(\frac{\left(N-N_{\theta }\right)!}{\left(N-N_{\theta }-N(A)+N_{\theta }(A)\right)!\left(N(A)-N_{\theta }(A)\right)!}\right)$$

*N* is the total number of lipids. For a calculation binding isotherms, Equation (14) can be treated with Stirling’s equation and subsequently inserted into Equation (13), which has to be added to the total free energy function.

### 2.3. Protein Binding and Membrane Penetration

**Figure 3:**
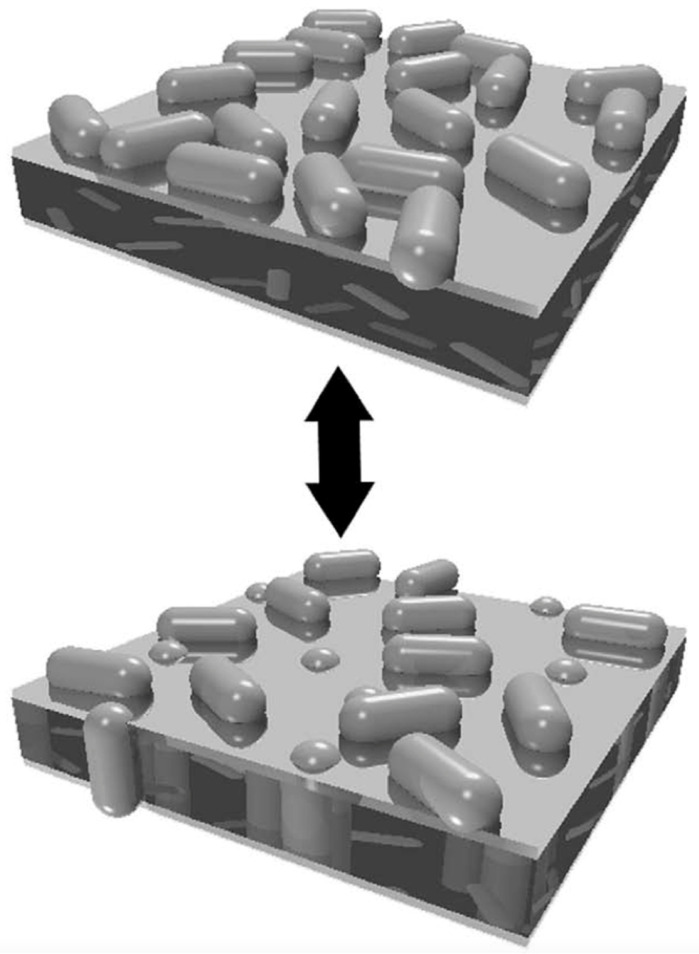
Illustration of two orientations of a rod-like protein bound to a membrane surface. Upper figure: All proteins are oriented perpendicularly to the surface normal. Lower figure: Mixture of proteins oriented perpendicularly and parallel to the surface normal. Taken from ref. [28] with permission. Elsevier, 2001.

I finish this section with a brief outline of a theoretical approach that takes membrane penetration of proteins into account. It is based on a theory developed by Minton [30]. Here, I utilize the representation of the theory by Zuckermann and Heimburg [28]. In his paper, Minton considered the possibility that a protein could adopt different structures with different orientations on the membrane surface (Figure 3). With respect to the membrane surface, the respective footprints of the protein would be different in form and size. Let us, for example, assume that proteins can be represented by a rod. If its main axis is parallel to the membrane surface, the footprint is a rectangle. If the orientation is perpendicular, the footprint is a circle or a square. The local pressure on the membrane in the latter scenario is larger and thus is the probability of insertion. Two rather general equations illuminate the conceptual aspects of the theory. The binding isotherm looks actually rather simple:

(15) $$K_{n}\left[P\right]=\tau_{n}\rho_{n}$$

where *K_n_* is the equilibrium constant related to the binding of the protein and adopting the *nth* conformation. The corresponding surface density of bound ligands is *ρ_n._* The factor *τ_n_*, which can be understood as an activity coefficient, contains the entire complexity of the binding process. Talbot used the scale particle theory to arrive at the following somewhat convoluted expression [36]:

(16) $$\tau_{n}=-ln\left(1-\left\langle \rho a\right\rangle \right)+\frac{A_{n}\left\langle \rho \right\rangle +s_{n}\left\langle \rho s\right\rangle /2\pi }{1-\left\langle \rho a\right\rangle }+\frac{A_{n}}{4\pi }\left[\frac{\left\langle \rho s\right\rangle }{1-\left\langle \rho a\right\rangle }\right]$$

where *A_n_* is the footprint area of the *nth* protein conformation on the membrane. The averages of protein density, footprint areas, and circumferences of the latter are denoted $\left\langle \rho \right\rangle$, $\left\langle \rho a\right\rangle$, and $\left\langle \rho s\right\rangle$, respectively. What Equation (16) tells us is that the equilibrium regarding a specific conformation *n* depends on how many other ligand conformations are already present. The formalism averages over all these conformations. Hence, Equation (16) resembles to some extent a mean field approach. If these averages for footprint area and circumference increase, the activity coefficient increases. If *K_n_* is a constant, *ρ_n_* decreases concomitantly. Zuckermann and Heimburg used this model to simulate binding isotherms just for the two protein orientations introduced above. Details of their formalism can be inferred from their paper [28].

Taken together, the contents of this section briefly outline the very basic physical principles that govern the binding of proteins to the surface of membranes. In what follows, I will discuss the binding of cytochrome *c* and α-synuclein in the light of these theoretical approaches.

## 3. Cytochrome *c*–Phospholipid Binding

### 3.1. Binding Isotherms

#### 3.1.1. Variation in Cytochrome *c* Concentration

In this section, I briefly discuss multiple studies of cytochrome *c* binding to anionic phospholipids at various conditions and with different binding protocols. The focus will be on the main (and as I show, to some extent contradictory) results rather than on a more detailed description of experimental protocols and experimental results, which can be found in a recent review [6].

Experiments exploring the binding of cytochrome *c* to anionic lipids can be generally divided into two categories. Category I involves the change in cytochrome *c* concentration in the bulk while keeping the concentration of anionic lipids and thus liposomes constant. Category II experiments keep the cytochrome *c* concentration constant and vary the concentration of lipids (liposomes). Generally, if the binding process was at equilibrium all the time, one would expect that both types of experiments should yield the same results in terms of equilibrium binding constants. However, as we will see below, this is not the case.

**Figure 4:**
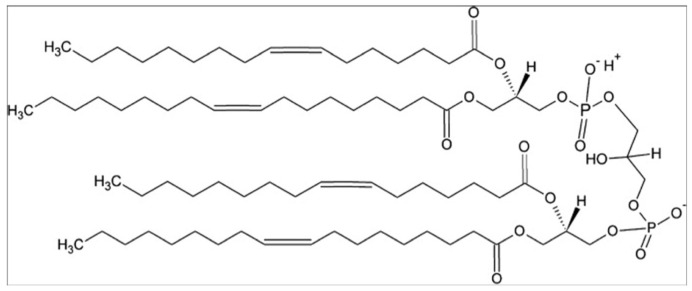
Structure of tetra-oleoyl-cardiolipin (TOCL).

Let us start with some of the experiments performed by the Kinnunen group in the nineties. Their results are still important, since they have guided the discussion and understanding of cytochrome *c* binding to cardiolipin-containing vesicles until today. Cardiolipin is the lipid of interest because its head group is the target of cytochrome *c* binding on the inner membrane of mitochondria. Figure 4 depicts the structure of 1,1′,1.2′-tetraoleoyl cardiolipin (TOCL) which has been used in many binding experiments. Its rather peculiar head group is a phosphodiester of glycerol. Each phosphate group is esterified with two oleic (18:1) acids. Its use in experiments is in part due to the fact that it cannot be oxidized when cytochrome *c* acquires peroxidase capability. However, other cardiolipin derivatives with different numbers of unsaturated bonds have been used in the field. Rytömaa et al. used cardiolipin from bovine heart, which contains polyunsaturated (two double bonds) fatty acid chains [37]. The liposomes used in their experiments (most likely small unilamellar vesicles, SUVs, based on their experimental protocol) contained 10 mol % of bovine cardiolipin, 85% egg phosphatidylcholine (PC), and 5% 1-palmitoyl-2[6-(pyrene-1-yl]-hexanoyl-*sn*-glycerol-3-phosphocholine (PPHPC). Phosphatidylcholine is zwitterionic at neutral pH and thus does not contribute to the net charge of the lipid membrane. The pyrene group in PPHPC was used as a fluorescence probe in that its emission is reduced by fluorescence resonance energy transfer (FRET) to the heme group of cytochrome *c* upon the binding to the membrane surface of the latter.

**Figure 5:**
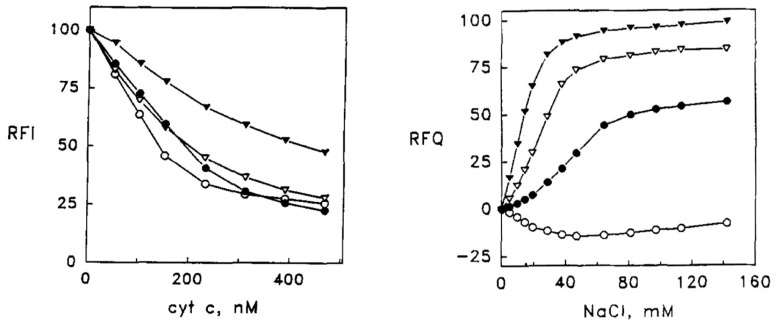
Fluorescence titration of oxidized cytochrome *c* to fluorescence-labeled egg PC with 10 mol% bovine cardiolipin. The total lipid concentration was 25 μM (2.5 μM total cardiolipin concentration). Left: Titrations performed at pH 7 (filled triangles), 6.0 (open triangles), 5.0 (filled circles) and 4.0 (open circles). Right: Fluorescence titrations of NaCl to a mixture of 470 nM cytochrome *c* and 25 μM total lipid concentration (2.5 total cardiolipin concentration) performed at the above pH. Taken from ref. [37] (open source).

Figure 5 shows the results of fluorescence titration experiments where oxidized cytochrome *c* was added to the above SUVs. Increasing the protein concentration leads to more protein binding and thus quenching of pyrene emission. The depicted titration curves were measured at the indicated pH. Upon lowering the pH from 7.0 to 6.0, the effective binding affinity and the degree of quenching increases significantly. A further, though slight, increase in binding affinity seems to occur at pH 4.

**Figure 6:**
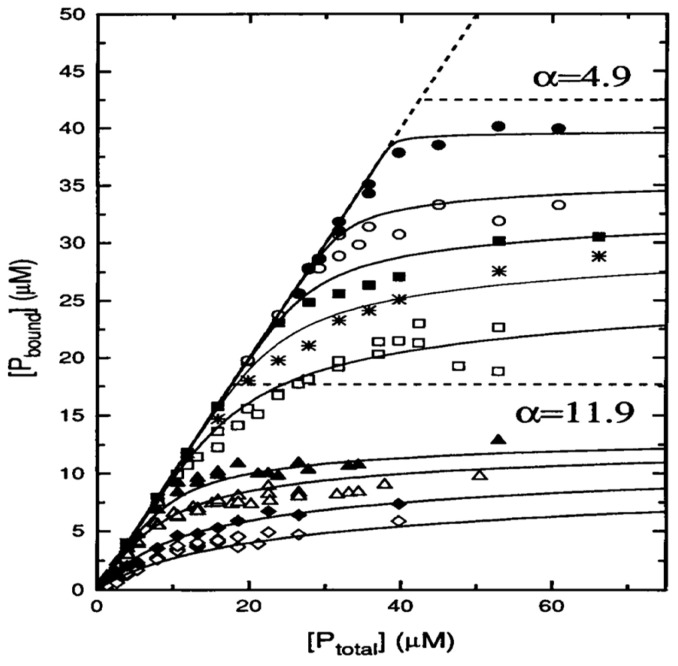
Binding isotherms of oxidized horse heart cytochrome *c* for 210 μM DOPG dispersion at different concentration of NaCl. From top to bottom: 0.21 (filled circles), 4.35 (open circles), 10.0 (filled rectangles), 16.9 (asterisks), 29.4 mM (open rectangles) (low-ionic-strength regime), 42.0 (filled triangles), 54.4 (open triangles), 79.4 (filled diamond) and 104.4 mM (open diamond) (high-ionic-strength regime). The solid lines result from global fits of the Heimburg–Marsh model to the data sets of these two ionic strength intervals. Taken from ref. [27] with permission. Elsevier 1995.

If the above binding process were governed by electrostatic interactions, one would expect a massive decrease in binding affinity in the presence of centimolar NaCl. This is nicely demonstrated by the isotherms that Heimburg and Marsh [27] obtained for cytochrome *c* binding to DOPG (dioleoyl-phosphatidylglycerol)-containing vesicles. The polar head group of this lipid carries a single negative charge at neutral pH. Figure 6 shows how the addition of NaCl (0.21 mM up to 104.4 mM) reduced the maximal number of bound proteins and the effective binding affinity. The solid curves in the figure resulted from fits with the Heimburg–Marsh theory outlined in Section 2. The fitting required the use of two stoichiometric factors α, i.e., 4.9 at low and 11.9 at high ionic strength. Let us now compare these findings with corresponding experimental results of Rytömaa et al. [37]. As shown in Figure 5, their data indicate rather complete dissociation of lipid-bound proteins at ca. 80 mM, in very good qualitative agreement with the results obtained with DOPG and predicted by theory. However, this inhibition effect of NaCl decreases with decreasing pH. At pH 4, the presence of NaCl seems to further increase the number of bound proteins. Similar inhibition effects were obtained for the addition of ATP, ADP, and AMP, and even more efficiently for the addition of MgCl_2_. Surprisingly, the Kinnunen group found in a subsequent study that the inhibition by both NaCl and trinucleotides disappears if the CL or alternatively the PG lipid fraction of the membrane increases [38,39]. For electrostatic binding, these observations seem to be counterintuitive.

In this and subsequent papers the authors interpret this finding as indicating a switch from an electrostatic binding via a lysine-rich site A on the protein to another mode of binding to a site C (Figure 1), which involves the formation of hydrogen bonding between N52 and protonated phosphate moieties. In order for this process to occur, they had to propose that the pK for the first protonation step of the CL-phosphate groups shift significantly to higher values due to repulsive electrostatic interactions between different negatively charged groups. Obviously, the shift could be expected to become more pronounced with increasing mole fractions of cardiolipin, which could lead to the observed reduction in NaCl inhibition. In addition to proposing A- and C-site binding, Kinnunen et al. in conjunction with Wallace and coworkers invoked the so-called lipid anchorage model, where one of the four lipid chains would be inserted into the cavity of bound cytochrome *c* (*vide infra*) [39,40,41].

**Figure 7:**
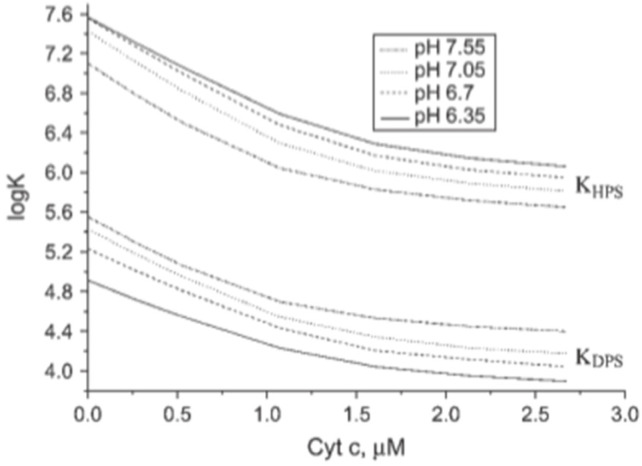
Dependence of the effective binding constant of oxidized cytochrome *c* to cardiolipin vesicles at the indicated pH calculated with the theoretical approach of Gorbenko et al. [32]. The lipid concentration was set to 60 μM. The assumed ionic strength was 5 mM. The fraction of acidic lipids in the vesicles was set to 0.4. The figure was taken from ref. [32] Elsevier, 2006.

None of the above works contains any theoretical analysis. This gap was filled by Grobenko et al. in a series of papers that combined theoretical analysis with FRET-based binding experiments. Details can be inferred from their papers, while a more detailed summary is given in ref. [32,42]. Their theory combined the scaled particle theory of Minton [30] with the electrostatic theory of Heimburg and coworkers (cf. Section 2 [29]. Contrary to Minton, the authors considered only a single protein conformation. In order to account for the pH dependence observed by Rytömaa et al. [37], they explicitly considered the influence of phosphate group protonation. Figure 7 depicts a plot of the calculated effective binding constant *K*_0_ as a function of cytochrome *c* concentration for single- (HPS) and double-deprotonated (DPS) CL head groups at the indicated pH values. Three aspects of the displayed results are noteworthy. Firstly, *K*_0_ decreases with increasing protein concentrations (decreasing lipid-to-protein ratio), which follows directly from Minton’s theory. Second, the binding affinity is higher at more acidic pH for both protonation states. Third, the binding affinity to the HPS (10^6^–10^7^ M range) is nearly two orders of magnitude higher than it is for DPS binding (10^4^–10^5^ M). This result seems to be counterintuitive, and makes sense only if one assumes a non-electrostatic component to be present in HPS.

#### 3.1.2. Variation of Lipid Concentration

**Figure 8:**
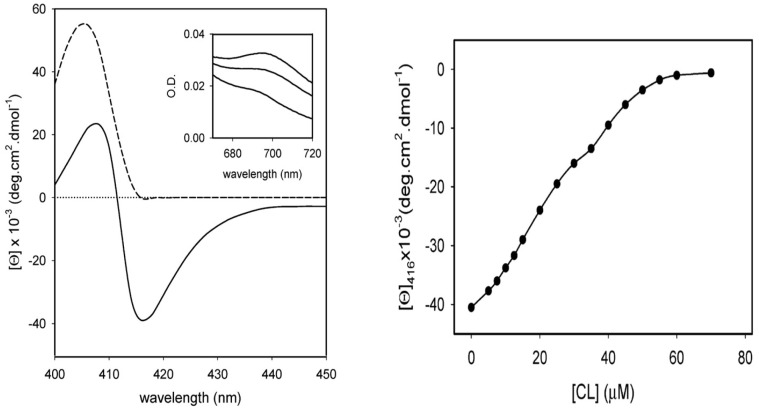
Left: Soret band circular dichroism spectrum of oxidized horse heart cytochrome *c* in the absence (solid line) and presence (dashed line) of vesicles with 60 μM cardiolipin. The protein concentration was 10 μM. The spectra were recorded at pH 7 and room temperature. Right: Molar ellipticity at 416 nm plotted as function of cardiolipin concentration. Taken from ref. [47] with permission. American Chemical Society, 2008.

Several research groups have conducted studies of cytochrome *c* binding to cardiolipin by varying the concentration of the latter. They have been described, compared, and discussed in detail in an earlier review [6]. Here, I summarize solely the main aspects. I start by briefly discussing binding studies at neutral or slightly alkaline pH. They have in common that they all used visible circular dichroism and tryptophan fluorescence as (indirect) markers. Regarding the former, experiments monitor the changes in the dichroism assignable to the Soret (B) band of the heme chromophore. The CD spectrum of the native unbound ferric state exhibits a very pronounced negative couplet assignable to the electronic transitions into the B_x_ and B_y_ states of the heme (Figure 8) [43,44]. The dichroism is mostly induced by electronic heme–protein interactions, peripheral substituents, and non-planar deformations of the heme group that eliminate the inversion center of the heme macrocycle [45]. If the protein undergoes partial unfolding upon its binding to the membrane surface, the couplet converts into a positive Cotton band, mostly due to the reduction in rotational strength of one of the B-state transitions caused by the removal of the M80 ligand and its second neighbor F82 (in horse heart cytochrome *c*), the interaction of which with the heme is the major source of the negative component of the native state couplet [46]. Another indicator of partial cytochrome *c* unfolding is the intensity of W59 fluorescence. It is mostly quenched in the native state due to FRET to the adjacent heme group. It increases if structural changes move W59 away from the active site, hence both Soret band CD and W59 fluorescence can be used to probe cytochrome *c* binding to surfaces if accompanied by structural changes.

I start with experiments carried out at neutral pH (7–7.5). Sinibaldi et al. added vesicles (size not provided in the paper) with 100% bovine heart cardiolipin to a fixed concentration of oxidized horse heart cytochrome *c* (10 μM) at pH 7 [47]. If one assumes a 50:50 partition between the inner and outer leaflets of their vesicles, the lipid-to-protein ratio varied between 0 and 3.5. As shown in Figure 8, a plot of the Soret band dichroism at 416 nm as a function of the (total) cardiolipin concentration seems to indicate two phases, which the authors analyzed with a Langmuir–Hill-like model based on the assumed existence of two different binding sites on the membrane surface. The authors did not identify these binding sites on the rather homogeneous surface of the unilamellar vesicles that they used for their experiments. However, the coexistence of two phosphate protonation states could offer an explanation (*vide supra*). If one corrects their binding constants for the above partition of lipids, one obtains *K*_1_ = 9.8 × 10^4^ M^−1^ and *K*_2_ = 4.8 × 10^4^ M^−1^. The corresponding Hill coefficients are 2 and 4, respectively. The fractions of the respective binding sites were found to be 0.22 and 0.78. In the presence of NaCl, the fraction of bound cytochrome *c* was substantially reduced, but a complete inhibition of binding was obtained only for 200 mM NaCl. In the presence of 100 mM, 20% of the proteins were reported to still interact with cardiolipin at high lipid concentrations. Thus, their results are at variance with what Rytömaa et al. observed at pH 7 (i.e., total inhibition above (NaCl) = 80 mM) [37]. Moreover, as shown below, they are not consistent with theoretical predictions based on the Heimburg–Marsh theory [27]. The binding affinities reported by Sinibaldi et al. are significantly lower than the ones obtained from the analysis of Grobenko et al. [32].

**Figure 9:**
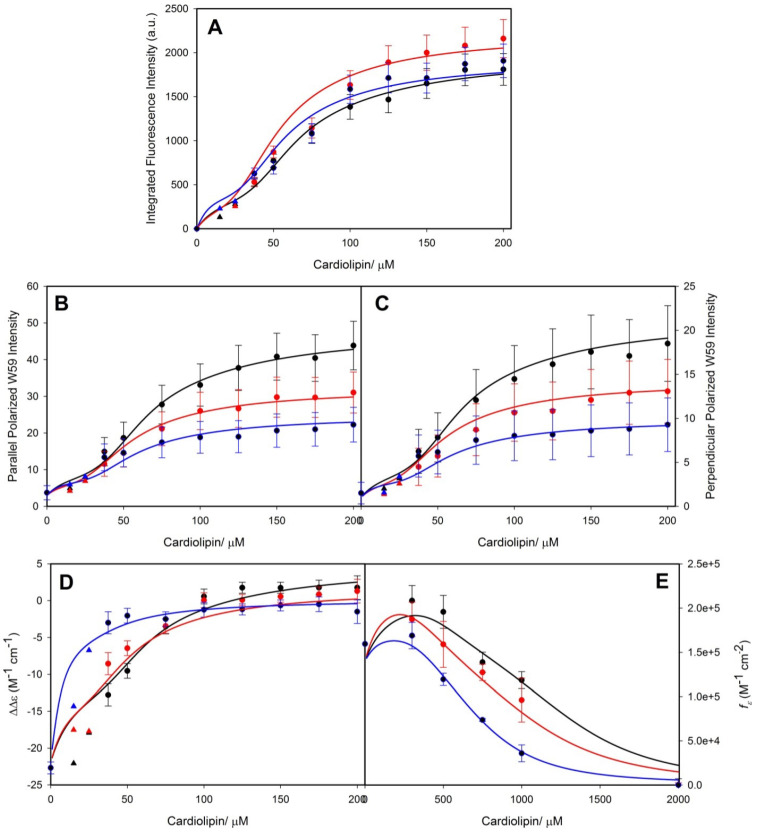
Binding of oxidized horse heart cytochrome *c* to TOCL/DOPC liposomes with 20% (black), 50% (red), and 100% (blue) CL content. (**A**) Plot of the integrated W fluorescence intensity, (**B**) the parallel and (**C**) perpendicularly polarized fluorescence intensity at 340 nm, (**D**) the ΔΔε of the couplet peak of the Soret band CD spectra, and (**E**) the oscillator strength of the 695 nm absorbance band as a function of the CL concentration. The protein concentration was 5 μM for CD and fluorescence measurements. The absorption spectra were recorded with 50 μM cytochrome *c*. The solid lines result from a global fitting procedure are described in detail in ref. [48], from where the figure was taken with permission. American Chemical Society 2015.

Very similar experiments were performed and analyzed by Pandiscia and Schweitzer-Stenner [48]. However, they used a broader set of experiments that encompassed W59 fluorescence, fluorescence anisotropy, fluorescence quenching, and the absorptivity of the charge transfer band at 695 nm, which is an indicator of M80 ligation. They used TOCL as the cardiolipin derivative and oxidized horse heart cytochrome *c*. Their binding isotherms cover a lipid-to-protein ratio regime between 2 and 40. SUVs with 20%, 50%, and 100% TOCL were used in their experiments. Figure 9 shows the titration curves obtained with the above spectroscopies. In an earlier paper, the authors analyzed data obtained with 25% CL SUVs in terms of a two-binding-site model, which they assigned to the protein rather than to the vesicle surface (note that the respective mathematical formalisms for these cases are different—for details, check ref. [49]). Here, I briefly discuss their second, much more realistic model. In order to explain the different phases indicated by the data, they invoked the following reaction scheme:

cyt + mCL ⇆ [cyt-mCL]_nf_ ⇆[cyt-mCL]_f_ (R1)

**Figure 10:**
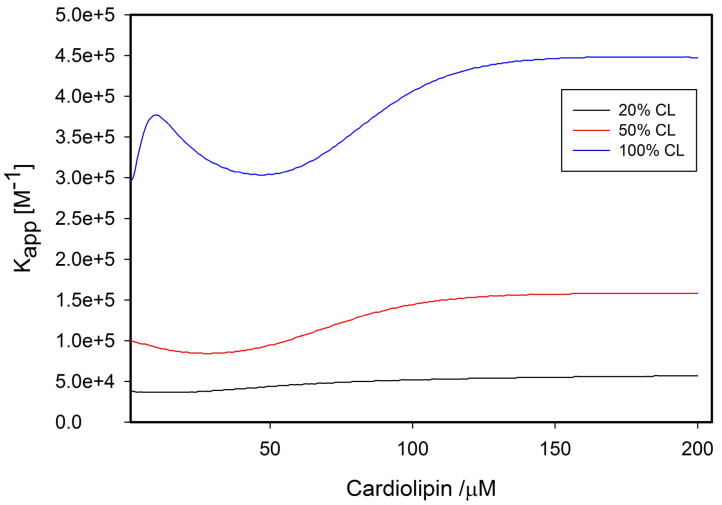
Plots of the effective equilibrium constant of cytochrome *c* binding to TOCL/DOPC vesicles as a function of cardiolipin concentration for the indicated fractions of TOCL in the employed SUVs. The underlying theory is sketched in the main text and described in detail in ref. [48], from where the figure was taken with permission. American Chemical Society, 2015.

The first step is just the binding of the protein to m cardiolipins. The structure changes modestly, but remains native-like with minimal W59 fluorescence (nf: non-fluorescing). On the surface, the protein undergoes a conformational change into a partially unfolded state that is fluorescing and in which M80 is replaced by another ligand. The respective equilibrium constants are *K* and *K_c_.* The binding process was modeled with the van der Waals gas model as utilized by Heimburg and Marsh (*vide supra*) [27]. *K_c_* was assumed to depend on the lipid/protein ratio. The respective relationship reads as:

(17) $$K_{c}=K_{c,low}f\left(\left[CL\right]\right)+K_{C,high}\left(1-f\left(\left[CL\right]\right)\right)$$

where *K_c,low_* and *K_c_,_high_* denote the *K_c_* values for the limits of very low and very high lipid concentration. The function in the second term is of the Hill type:

(18) $$f\left(\left[CL\right]\right)=\frac{{\left(\chi_{b}/K_{mod}\right)}^{m}}{\left(1+{\left(\chi_{b}/K_{mod}\right)}^{m}\right)}$$

where *χ_b_* is the mole fraction of bound ligands and *K*_mod_ is some type of equilibrium constant that determines the midpoint of the transition between the two *K_c_* values. The Hill coefficient is denoted *m.* The *K_c_* value is low at low cardiolipin concentrations where the molecular crowding inhibits protein unfolding, which becomes possible at high lipid concentrations, where the protein surface density is lower. This model was used for a global analysis of the data in Figure 9. Figure 10 shows the apparent equilibrium constant *K_app_ = K · K_c_* as a function of cardiolipin concentrations for 20%, 50%, and 100% CL content of the utilized SUVs. The differences between the respective curves predominantly reflect the CL density and only to a limited extend different equilibrium constants (*K_o_* = 20, 20 and 35 M^−1^ for 20%, 50%, and 100% TOCL). A detailed listing of all thermodynamic and spectroscopic parameters determined from the above fitting is given in ref. [48]. The increase in the binding affinity at high lipid-to-protein ratios results from the equilibrium shift towards the partially unfolded *f* state.

Another outcome of the above analysis has to be emphasized. The authors found that the addition of NaCl reduces the amplitude of binding isotherms rather than the equilibrium constant *K.* A similar observation was more recently reported for the binding of human cytochrome *c* to cardiolipin-containing nanodiscs at pH 7.4 [50]. These observations are at odds with predictions from the electrostatic theory in Section 2. They are also at variance with the data of the Kinnunen group [37], but in qualitative agreement with the observations of Sinibaldi et al. [47]. Pandiscia and Schweitzer-Stenner explained these results with a model that invokes an equilibrium shift from *f* to *nf* conformations with increasing NaCl concentrations due to sodium binding to lipid surface groups [49].

A significant difference between the binding experiments of the above groups should not go unnoted. While the binding data of Pandiscia and Schweitzer-Stenner cover a broad range of lipid-to-protein ratios, the data of Sinibaldi et al. were all measured in the low lipid-to-protein regime (*vide supra*). Hence, the two phases indicated by the binding isotherm in Figure 8 are not identical with the ones inferred from the spectroscopic data for 100% TOCL vesicles in Figure 9. The latter do not provide sufficient resolution to resolve the two phases. Hence, a more complete model should different populations of native like states at low lipid-to-protein ratios.

The results of experiments carried out by varying the lipid concentration all suggest that the protein undergoes conformational changes on the surface of anionic lipid vesicles. The behavior of the systems in the presence of NaCl is inconsistent with what one would expect for a purely electrostatic A-site binding, but it can be explained by a model where the binding of sodium atoms to the membrane changes the conformational equilibrium on the surface of the used vesicles [48]. The latter offers several targets for the sodium cation, namely, the negatively charged phosphate groups and the C=O groups of ester linkage between the head group and the fatty acid chain [51].

**Figure 11:**
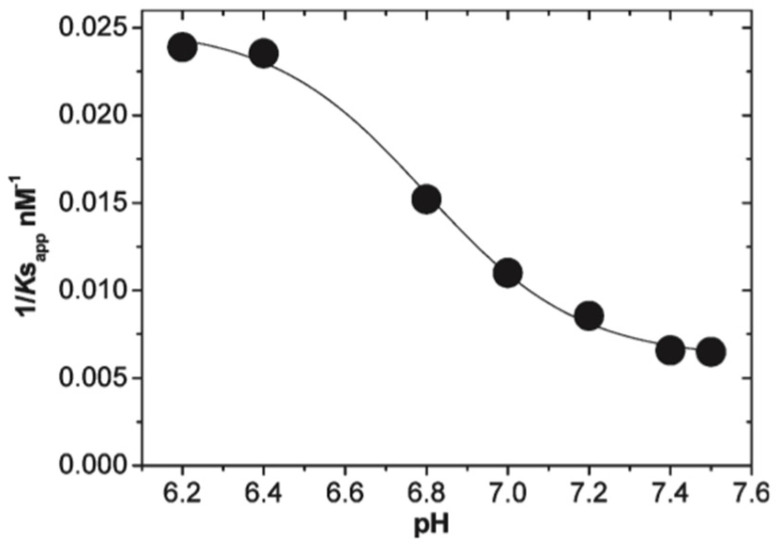
Effective binding affinity of cytochrome *c* binding to the inner mitochondrial membrane plotted as a function of pH. The solid line is the result of a fit of a Henderson–Hasselbach-type equation to the experimental data. Experimental details can be obtained from ref. [51,52], from where this figure has been taken with permission. American Chemical Society 2009.

The results discussed above regarding binding studies of Kinnunen and coworkers suggest that the apparent affinity for cytochrome *c* binding to cardiolipin-containing membranes increases if the pH of the solution is lowered below 7.0 (Figure 5) [37,39]. They attributed this observation to a switch from A-side to C-side binding (Figure 1). However, more recent experiments by Nantes and coworkers led to a rather different picture [52,53]. First, they observed that the addition of horse heart cytochrome *c* to vesicles with 20% bovine heart cardiolipin produced significant turbidity in a pH range between 3 and 7. They interpreted their finding as reflecting vesicle fusion due to interactions of cardiolipin with two different binding sites. The first one was the aforementioned site A, the other one was a novel site L. Mapping via site- specific acetylation and carbethoxylation led them to suggest that residues K22, K27, K87, and H33 are all involved in what they called L-site binding (cf. Figure 1). In a subsequent paper, Kawai et al. examined the apparent affinity of cytochrome *c* binding to inner mitochondrial membranes as a function of pH [52]. The authors determined the binding affinity indirectly from double reciprocal plots of respiration rates as a function of cytochrome *c* concentrations. Figure 11 shows that the binding affinity increases with decreasing pH between 7.5 and 6.1, in qualitative agreement with the results of Rytömaa et al. The pH dependence looks like a titration curve, with an effective pK value of ca. 6.8 [37].

**Figure 12:**
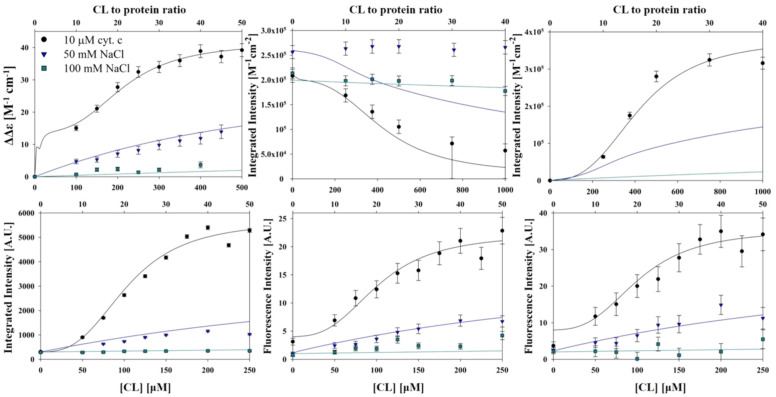
Ferricytochrome *c* binding to 20 mol% TOCL/80 mol% DOPG SUVs at pH 6.5 probed by different spectroscopies in the absence and presence of NaCl: Soret band circular dichroism (top left) and integrated intensities of a charge transfer band at 695 nm (top middle) and 625 nm (top right); integrated W59 fluorescence intensity (bottom left), polarized fluorescence I_vv_ (bottom middle), and I_vh_ (bottom right) (v: vertical, h: horizontal polarization). Note that the corresponding data points in the different figures were obtained over the same range of cardiolipin-to-protein ratios. The error bars of the CT1 data points at the two highest cardiolipin concentrations are asymmetric and indicate rather large errors due to uncertainties with regard to the choice of the baseline. The solid lines result from fits and simulations described in the main text and in more detail in ref. [54], from where this figure was taken (open source).

If the increase in binding affinity at mildly acidic pH is indeed assignable to the proposed L site, the binding process should be electrostatic in nature, contrary to what one would expect from C-site binding. This issue was addressed by Milorey et al., who combined Soret band CD, W59 fluorescence (polarized and unpolarized), 695 nm absorption, and resonance Raman spectroscopy to examine the binding of oxidized cytochrome *c* to SUVs with 20% TOCL and 80% DOPC in the absence and presence of NaCl at pH 6.5 [54,55]. As observed for the binding at pH 7.4, the data indicated a structural conversion into a partially unfolded state at high lipid-to-protein ratios (low protein surface density). However, contrary to results obtained at pH 7.4, the spectral changes were found to be significantly reduced at 50 mM and practically absent at 100 mM NaCl. In line with this result, W59 fluorescence was found to decrease significantly with increasing salt concentration (Figure 12). Milorey et al. analyzed their data by combining the NaCl binding model invoked to simulate binding data at pH 7.4 with the electrostatic model of Heimburg and Marsh (Section 2). The authors did not consider the influence of lipid demixing. Their analysis clearly revealed that L-site binding is predominantly electrostatic in nature, very much in agreement with Nantes and coworkers. In agreement with their work, the effective affinity was found to increase with decreasing pH at high lipid concentrations. While the partially denatured state populated at pH 7.4 still contained hexacoordinate low-spin heme iron absorption (increase from the 625 nm band documented in Figure 12) and resonance Raman data recorded at pH 6.5 indicate a mixture of low-spin and high-spin hemes (hexa- and pentacoordinate).

The already rather complex picture of cytochrome *c*–cardiolipin interactions has been further enriched by an NMR study of O’Brien et al., who used two-dimensional NMR to determine contact points between cardiolipin-containing reverse micelles and cytochrome *c* [56]. Besides identifying amino acid residue in the A- and L-site regions, they found contacts in what they called an N-site region composed of F36, G37, T58, W59, and K60. There is no information about whether N-site binding is electrostatic in character. Since it contains only a single lysine residue, this seems to be unlikely. T85, W59, and K60 all have hydrogen bonding capability, which would make the N site more C site-like.

**Figure 13:**
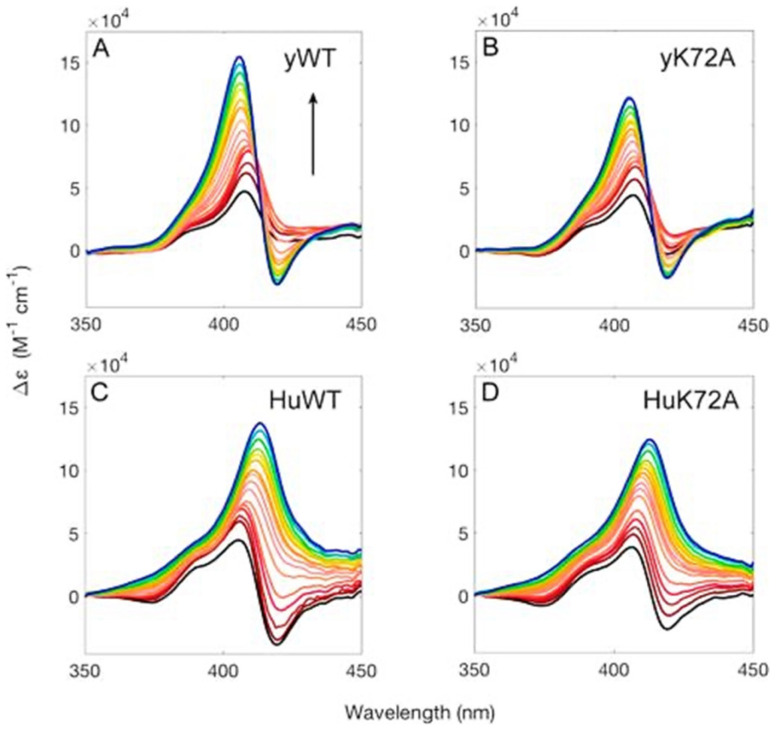
Soret band CD spectra of 10 μM (**A**) wild-type yeast-iso-cytochrome *c*, (**B**) its K72A mutant, (**C**) human wild-type cytochrome *c*, and (**D**) its K72A mutant measured at different concentration of 100% TOCL lipids forming 100 nm LUVs. The black arrow indicates the direction of the changes of the positive Cotton band with increasing lipid concentration. The respective black spectra represent the spectrum in the absence of lipids. The maximum concentration of lipids in the outer leaflet was 100 μM (blue spectrum). Taken from ref. [57] with permission. American Chemical Society 2017.

I finish this section by briefly discussing similar binding experiments carried out by Elmer-Dixon et al., who compared the binding of different yeast and human cytochrome *c* mutants to cardiolipin membranes [57]. At pH 7.0, where L- and A-site binding (or whatever is involved at pH higher than 7) might be mixed [52,55], the apparent binding affinity of yeast-*iso 1*-cytochrome *c* is higher than the one of the horse heart derivative. Normally, the ε-NH_2_ group of K72 is methylated in the former, but this modification is missing if the protein is expressed in *Escherichia coli.* Interestingly, the binding of yeast cytochrome *c* to cardiolipin vesicles is not inhibited by the presence of NaCl. This observation is very surprising in that it rules out the involvement of A- and L-site binding at neutral pH. Surprisingly, this phenomenon has not really been appreciated in the literature. Elmer-Dixon et al. used Soret CD and W59 fluorescence as indicators of cytochrome *c* binding to cardiolipin-containing vesicles. For their experiments, they selected a pH value of 8 in order to ensure the absence of L-site binding and a dominance of electrostatic binding [50]. However, this pH value moves the system into a regime where the alkaline state IV becomes populated [58,59]. Figure 12 compares the Soret CD spectra of wild-type yeast and human cytochrome *c* (yWT and HuWT) and of their respective K72A mutants in the presence of an increasing amount of cardiolipin-containing LUVs (100% TOCL). While the CD signal of HuWT is reminiscent of the one reported for horse heart cytochrome *c* (i.e., a couplet converts into a positive Cotton band with increasing cardiolipin concentration), the CD spectrum of yWT has only a positive component that seems to represent just one of the two B-state transitions with a vibronic structure at shorter wavelengths [44]. As indicated by the authors, this spectrum could be indicative of a significant state IV population [60]. Interestingly, a couplet emerges at higher cardiolipin concentrations. Differences between the CD spectra of wild type and mutants are quantitative (slightly reduced amplitudes) rather than quantitative. The authors used simple Hill functions (*vide supra*) to fit their CD- and fluorescence-based binding isotherms. As one can infer from the thermodynamic parameters in Table 1, the influence of the K72A mutation is moderate for yeast and practically absent for human cytochrome *c*. Interestingly, however, the dissociation constants inferred from the fluorescence isotherms are substantially larger than the one obtained from the corresponding CD data. This discrepancy clearly shows that a single-step model cannot reproduce these data consistently. This notion is further corroborated by the absence of isodichroic points in the series of spectra shown in Figure 13.

In a follow-up study, Elmer-Dixon et al. [61] used their spectroscopic tools to investigate the binding of several yeast cytochrome *c* mutants to 100% TOCL LUVs (K72A, K73A, K72AK73A, K86A, K87A, K86/87A). The respective dissociation constants obtained from the CD-based isotherms varied to a very limited extent (slightly decrease for K72A, slight increase for K73A and K72AK73A). For the fluorescence data, the increase induced by the K72 and K73 mutations was more pronounced. The authors qualitatively explained the differences between CD and fluorescence titration with the two-step binding model introduced above. If the latter applies, it is obvious that the mutation-induced changes in the fluorescence-based equilibrium constants affect solely the fluorescing partially unfolded state. This view would be fully consistent with the findings of Pandiscia and Schweitzer-Stenner [48], but it would be at variance with a classical understanding of A-site binding, which is purely electrostatic in nature.

#### 3.1.3. Summary

The binding experiments described in Section 3.1.1 and Section 3.1.2 have not yet produced a consistent understanding of cytochrome *c* binding to cardiolipin-containing membranes. The early experiments by Kinnunen and coworkers seem to suggest a very clear picture, i.e., electrostatic A-site binding at neutral and C-site binding (including lipid insertion) at acidic pH. However, binding experiments conducted at nearly neutral (pH 7.4) and mildly acidic pH (6.5) are at variance with such a model. At pH 7.4, only the structural conversion that follows the initial binding step seems to be electrostatic in nature. Experiments at pH 7.4 with yeast cytochrome *c* confirm this view. However, at pH 8, the binding of yeast cytochrome *c* is entirely electrostatic in nature [50]. At mildly acidic pH, binding becomes predominantly electrostatic (L-site binding). None of the experiments that involved the change in cardiolipin rather than in protein concentration are indicative of the proposed C-site binding. The N-site binding inferred from NMR data could constitute the initial binding step above pH 7.0, which would explain its insensitivity to the presence of NaCl (for horse heart) and A-site mutations (yeast and human cytochrome *c*).

My discussion of the experiments in this section did just touch the possibility that cytochrome *c* could penetrate the lipid membrane, which would add a strong hydrophobic component to the binding free energy. I will discuss this issue in the following Section 3.2. and Section 3.3. Obviously, all the CD and fluorescence experiments reported above clearly indicate structural changes on the surface. The model of Pandiscia and Schweitzer-Stenner involves two sequential steps, i.e., the binding to the surface followed by a conformational change, the probability of which increases with increasing lipid-to-protein ratios. I will return to this model in Section 3.2, where I outline different types of experiments that lead to somewhat different views of whether such conformational changes occur nor not.

**Figure 14:**
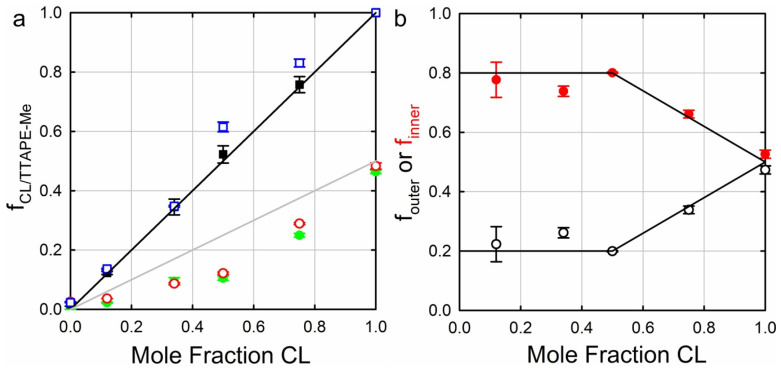
Partitioning of TOCL between inner and outer leaflets in mixed TOCL–DOPC lipid vesicles. (**a**) TOCL fraction in leaflets plotted as a function of TOCL concentration (peak intensities of fluorescence indicator: solid green circles, outer leaflet; solid black squares, both leaflets; integrated emission: open red circles, outer leaflet; open blue squares, both leaflets). Pure CL vesicles exposed to TTAPE-Me on both leaflets are used as the reference state for f CL/TTAPE-Me = 1. The expected linear dependence of the measured fluorescence on the TOCL mole fraction is shown for homogeneously mixed TOCL/DOPC vesicles for the outer leaflet (gray line) and for both leaflets (black line). (**b**) Fraction of total TOCL detected on the outer (open black circles) and the inner leaflet (inner leaflet, solid red circles) plotted as a function of CL mole fraction for CL/DOPC vesicles. The black lines are only meant to guide the eye. Taken from ref. [62] with permission. American Chemical Society 2019.

The studies thus far described in this and the preceding section were carried out with vesicle models of the inner mitochondrial membrane. However, these might be oversimplifying model systems for a variety of reasons. One reason is that the mitochondrial membrane exhibits cristae with a concave surface, while the surface of model vesicles is of course convex. In order to arrive at a more biologically realistic picture of cytochrome *c* binding to cardiolipin, Elmer-Dixon et al. investigated the interaction of the protein with the inner leaflet of cardiolipin-containing vesicles [62,63]. To accomplish this task, the authors had to form the vesicles in the presence of the protein and take the non-incorporated ones out once the liposomes were formed. In a first study, the above authors labeled cardiolipin and DOPC with a fluorophore (1,1,2,2-tetrakis [4-(2-trimethylammonioethoxy)phenyl]ethene) to determine the partition of the lipids in the outer and inner leaflets of the membrane. The result of this investigation is visualized in Figure 14. The data plotted therein reveal a 4:1 distribution of cardiolipin (TOCL) between the inner and outer leaflets up to a cardiolipin fraction of 50%. Of course, the ratio approaches 1:1 if the cardiolipin content is increased further. On the contrary, the number of DOPCs was found to be the same in both leaflets. Until this paper was published, a 1:1 ratio was generally assumed for cardiolipin. As a consequence, the equilibrium constants of the binding reactions were generally underestimated by a factor 4, at least for DOPC–TOCL mixtures. I illustrate the significance of the apparent binding affinities reported by Pandiscia and Schweitzer-Stenner at high lipid/protein ratios. They reported 5 × 10^4^ M^−1^, ca. 10^5^ M^−1^, and 4.5 × 10^5^ for TOCL fractions of 20, 50, and 100 mol%. Implementing the above correction leads to 2 × 10^5^ M^−1^, ca. 4 × 10^5^ M^−1^, and 4.5 × 10^5^, respectively. The question arises as to what extent the observed asymmetry depends on the choice of the co-lipids of TOCL and the choice of the cardiolipin species itself.

Based on the above exploration of the TOCL partition over the inner and outer leaflets, Elmer-Dixon et al. explored the binding of cytochrome *c* in the interior TOCL/DOPC LUVs. Since this work puts an emphasis on structural changes, it is discussed in the subsequent section.

### 3.2. Conformational Changes

As already delineated above, the switch of cytochrome *c* function from electron transfer protein to peroxidase involves the protein’s binding to cardiolipin. In vivo, this rather special lipid constitutes a substantial fraction of the inner membrane of mitochondria. In its native state, the protein is a very poor peroxidase owing to the presence of two axial ligands and their stabilization of a low-spin iron state. Hence, one would naturally expect the need for the protein to undergo a conformational transition, in line with results from Soret band CD and W59 fluorescence measurements already outlined in the last section. However, a survey of the literature does not provide an unambiguous picture in this regard. The most relevant experiments are briefly described in this section. An important related issue is how the protein manages the performance of both functions in its biological environment. If the conformational changes described in Section 3.1.3 really occur, the protein will not be able to function as an electron transfer system.

**Figure 15:**
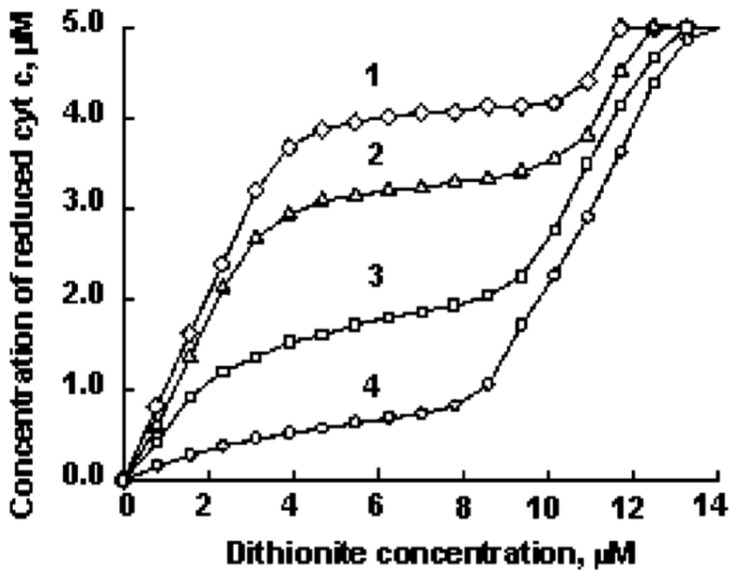
Concentration of reduced cytochrome *c* in the presence of TOCL vesicles and 8 μM gallocyanine plotted as a function of sodium of dithionate for different lipid-to-protein ratios: 25 (data set 1), 50 (data set 2), 100 (data set 3), and 200 (data set 4). Taken with permission from ref. [64] with permission. American Chemical Society 2007.

I start with a brief account of the work of Basova et al. [64]. These authors measured cyclic voltammograms of cytochrome *c* covalently attached to carboxylic acid-terminated monolayers covering gold electrodes with and without the presence of vesicles formed with 50 mol% mixtures of TOCL and DOPC. In the presence of the latter, the redox potential moved from very positive to very negative values, which was clearly indicative of a change in axial heme ligation. In a second series of experiments, Basova et al. carried out a titration with sodium dithionite in the presence of gallocyanine, which has an *E*^0^ value of 20 mV. Dithionite is a strong reducing agent owing to its negative *E*^0^ value of −564 mV. Figure 15 shows a plot of the concentration of ferrocytochrome *c* as a function of dithionite concentrations for different cardiolipin/protein ratios. At low ratios, the protein becomes reduced by dithionite, while the dye remains in the oxidized state. However, at high cardiolipin-to-protein ratios, a much larger amount of dithionite had to be added to achieve protein reduction. Concomitantly, the dye switched to its reduced state. These results strongly suggest that the redox potential of the protein changes from very positive (>100 mV) to much lower positive or even negative values at high lipid concentrations. In other words, the protein can function as a normal electron transfer protein at high surface occupation, but loses this capability if the surface density is low. These results are very much in line with the model of Pandiscia and Schweitzer-Stenner in that their *f* state is likely to exhibit a negative redox potential owing to the absence of the methionine ligand [48].

**Figure 16:**
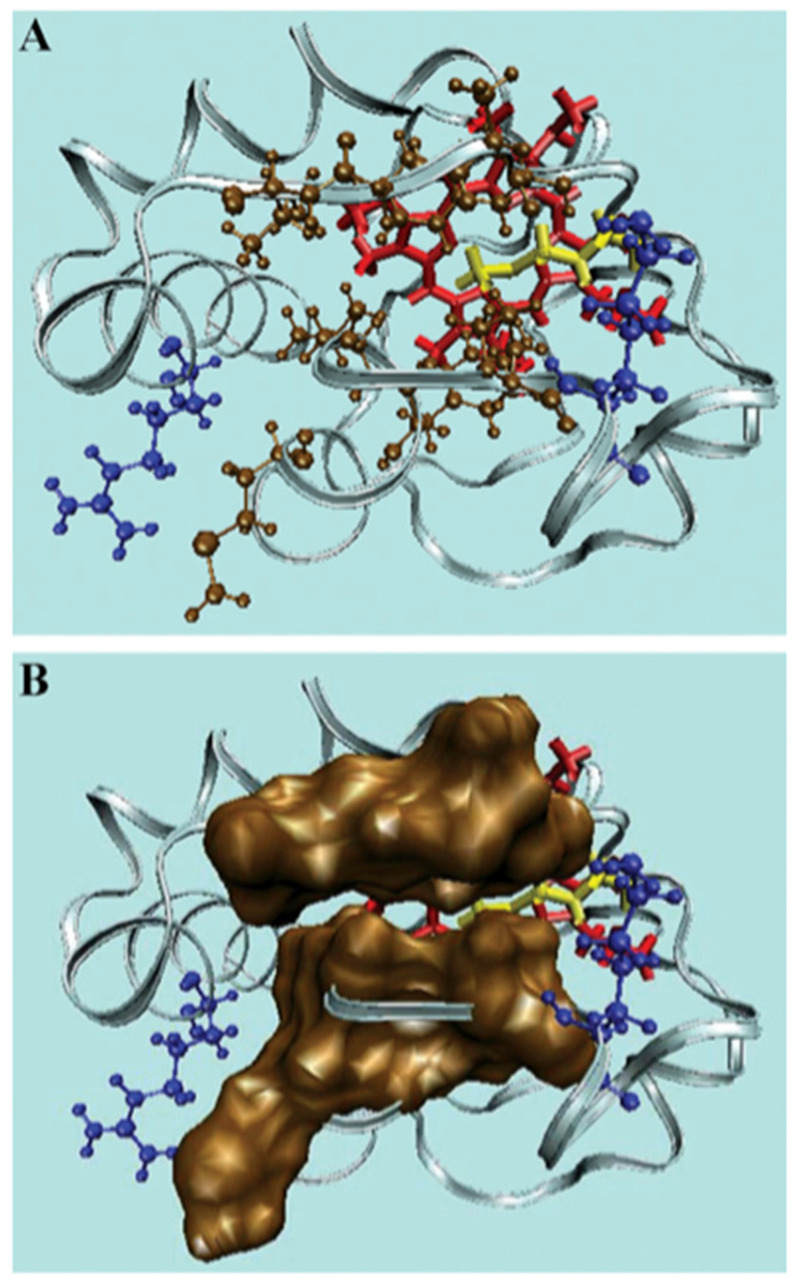
Illustration of the hydrophobic crevice into which a cardiolipin acyl chain could fit. It is formed by the segments 67–71 and 82–85. (**B**) is a space-filling representation of (**A**). Taken form ref. [41] with permission. Portland Press 2007.

As indicated above, Kinnunen, Wallace, and their respective coworkers proposed the so-called lipid anchorage model that proposed the insertion of a single-cardiolipin chain into the interior of the protein [40,41]. The hydrophobic channel that could allow this process to occur is shown in Figure 16. A summary of the experimental evidence for this process is given in an earlier review [6]. Here, I just focus on the work of McClelland et al., who showed by means of X-ray crystallography that a well-defined binding pocket for various hydrocarbons is formed in domain-swapped dimers of yeast *iso*-1-cytochrome *c*. In their experiments, three different detergents (***5***-cyclohexyl-1-pentyl-ß-D-maltoside, ***6***-cyclohexyl-hexyl-ß-D-maltoside, ω-undecylenyl-β-D-maltopyranoside) were allowed to react with the formed dimer and were found to penetrate the above binding pocket. In all three cases, the respective hydrocarbon chains are in close proximity to the heme group and the aromatic residues Y48, Y67, and W59. Y67 has been implicated as playing a major role in cardiolipin oxidation. Results of additional docking studies by the authors suggest that the C11 site of incorporated linoleic acid, the most common cardiolipin fatty acid chain, is close to Y67. In line with the above observation of a hydrophobic binding pocket, Steele et al. found that domain-swapped dimers of human cytochrome *c* bound to cardiolipin-containing nanodiscs at pH 8 are a more efficient peroxidase than the corresponding monomer [50,65,66]. Apparently, such a process would require close proximity of the lipid to the active center of the protein. However, there are unresolved issues regarding this model. First, it is not clear whether or not lipid insertion is only involved in C-site binding, as assumed by Rytömaa et al. [37,39] and McClelland et al. [67], or whether it requires a preceding electrostatic binding, as suggested by Kalanxhi and Wallace [41]. Second, it is obvious that a lipid insertion process would encounter a significant entropic barrier, which should slow down the binding process considerably. Third, one could ask to what extent cytochrome *c* binding would be reversible on a time scale probed by the binding experiments described above. Fourth, if A- and C-state binding both involve lipid insertion, A-state binding should not be inhibited by the late (after binding) addition of NaCl, contrary to experimental findings [49]. None of the results from binding experiments discussed thus far are indicative of irreversible binding that would lead to a linear binding isotherm up to a concentration where target vesicles are saturated. Fifth, one has to ask to what extent the obtained insertion of hydrocarbon chains into the binding pocket in domain-swapped dimers is representative of any process on cardiolipin-containing membranes.

**Figure 17:**
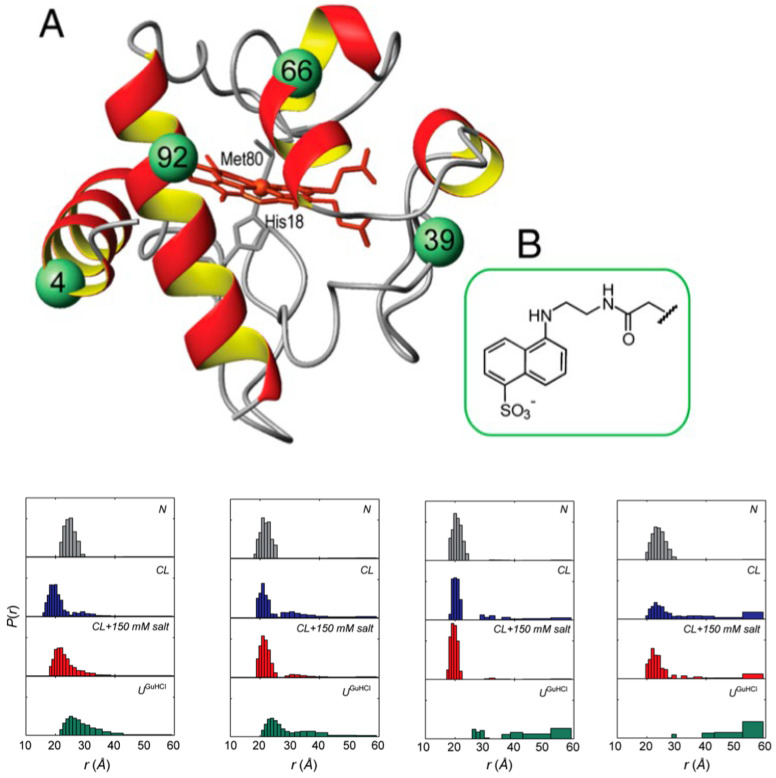
Upper: Structure of cytochrome *c* (**A**) with the positions of the individual dansyl labels (**B**) utilized for the FRET experiments of Hanske et al. [70], from where the figure was taken with permission. National Academy of Science, 2012. Lower: Distributions of donor (fluorophores in Figure 16)–acceptor (heme) distances for the four dansyl-labeled variants of oxidized cytochrome *c* (from left to right: Dns4, Dns39, Dns66, and Dns92) obtained from FRET experiments at pH 7.4 (native (N), gray); with 50 mol% TOCL–50 mol% DOPC liposomes and 660 μM total lipid concentration (CL-bound (CL), blue); with 50 mol% TOCL–50 mol% liposomes and 660 μM total lipid and 150 mM NaCl (TOCL + NaCl, red); and in 5.8 M GuHCl solution at pH 7.4 (GuHCl-unfolded (U GuHCl), green). The figure was taken from ref. [70] with permission. National Academy of Science, 2012.

The most detailed and direct experimental evidence for a conformational change in cardiolipin-bound cytochrome *c* has come from the time-resolved FRET experiments of Pletneva and coworkers. Their experimental concept and some of their results are shown in Figure 17. They replaced four amino acid residues of the native protein (indicated by green spheres) by cysteine and labeled the latter with dansyl. The emission of this fluorophore overlaps with the Soret band absorption of the heme, thus enabling fluorescence energy transfer from the former to the latter. Any structural change varies the efficiency of energy transfer. By performing single-molecule experiments, the authors obtained distance distributions for each of the four donor–acceptor pairs. The corresponding histograms are shown in Figure 17. Note that the experiments were carried out with 50 mol% mixtures of TOCL and DOPC LUVs at a rather high lipid/protein ratio of 500. At such a concentration, one can expect that all proteins are actually bound to the LUVs’ surface. All experiments visualized in Figure 17 were carried out at pH 7.4. Only the reference experiment carried out with 0.2 M guanidinium hydrochloride was performed at pH 5.8 to ensure unfolding of the protein. In the presence of LUVs, the peak of the distributions shifts to lower distance values, but this change is accompanied by a significant broadening towards larger distances, which is particularly pronounced for the dansyl attached to position 92, which resides in the helical high-stability C-terminal foldon of the protein, which contains also the M80 ligand of the heme group [68]. In the presence of 150 mM NaCl, the data are indicative of a redistribution towards shorter distances. Hanske et al. interpreted their results as reflecting the coexistence of two states, a compact and very native-like one (termed C in their paper) and an extended one (termed E). The distributions depicted in Figure 16 suggest that the free energy minimum associated with the latter is rather shallow and allows for rapid fluctuations (compared with the fluorescence lifetime of dansyl). Hong et al. investigated the dependence of the E population on the cardiolipin concentration and found that the increase in the former with the latter can be described by a sigmoidal function, which reaches 60% at a mole fraction of 50% TOCL [69]. They also investigated how the distribution changes with increasing ionic strength (i.e., NaCl concentration) and confirmed the observation of redistribution of conformations from the E towards the C ensemble.

**Figure 18:**
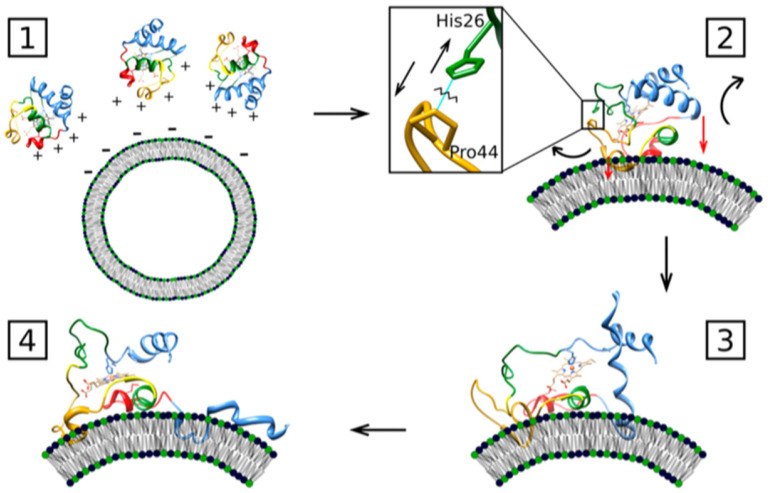
Schematic representation of a proposed mechanism of oxidized cytochrome *c* unfolding upon contact with cardiolipin-containing liposomes. (1) Positively charged cytochrome *c* and negatively charged liposomes are attracted electrostatically, thus promoting the binding of the protein to the membrane within milliseconds. (2) A protein surface region near K72, K73, K86, and K87, as well as D50, E66, and E92, are proposed contact sites for binding to the membrane. The H26−P44 hydrogen bond breaks upon binding of cytochrome *c* to the surface. (3) The low-stability Ω loop containing M80 rearranges, which leads to a rupture in the M80−Fe^3+^ coordination. In addition, unfolding of the green and yellow foldon units leads to a loss of tertiary structure. (4) The 50 s helix and the beginning of the C-terminal helix partially penetrate into the membrane, thus anchoring the protein, while the 60 s helix lies rather flat on the membrane surface. The 40 s Ω loop is close to the membrane while still staying in relatively close proximity to the heme group. The end of the C-terminal helix unfolds and N- and C-terminal helix contacts are broken. These structural changes lead to an extended protein conformer E with an easily accessible heme group. Taken from ref. [71].

A follow-up paper from Muenzner et al. [71] provided some details about the E state based on kinetic and spectroscopic data. Details of the experiments can be inferred from their paper. Their binding model is visualized in Figure 18. They propose that the C→E conversion involves a breaking of the H26–P44 hydrogen bond, which to a significant extent stabilizes the native tertiary structure. Patches containing K72, K73, K86, and K87 as well as N50, Q66, and E92, are contact sites for the protein’s interaction with cardiolipin. The former are classical A-site residues, while two of the latter are in the less well characterized C-site region indicated in Figure 1. They belong to the so-called green foldons, which together with the yellow foldons are unfolded in state E. This involves a rupture of the M80 coordination with the heme. Part of the still-existing helical segments (C-terminal helix) insert partially into the membrane, thus anchoring the protein. The 60 s helix in the green foldon and the so-called Ω loop lie close to the surface, but do not penetrate. Muenzner et al. proposed that the E state should have peroxidase activity, in line with the considerable peroxidase activity that Hanske et al. observed at a very high cardiolipin (total concentration of 660 μM)-to-cytochrome *c* (5 μM) ratio [70]. The reduction and the decrease in the rate of the peroxidase reaction in the presence of NaCl clearly indicates the involvement of state E.

Qualitatively, the results of the Pletneva group and of earlier binding studies by Pandiscia and Schweitzer-Stenner seem to corroborate each other. With regard to the influence of NaCl, the results from both groups suggest a redistribution of conformations from E to C (*f* to *nf*) rather than the inhibition of binding observed for L-state binding. Differences, however, are noteworthy. While Hong et al. reported a very pronounced dependence of E-state occupation on the LUV’s cardiolipin content (0.19 at 20 mol% TOCL up to 0.6 at 50 mol% TOCL) [69], the corresponding change reported by Pandisica and Schweitzer-Stenner is much more modest (0.5 to 0.58). There also differences between their understanding of the C state. While Pletneva and coworkers considered the observed distribution as a mixture of unbound native and bound native-like proteins, the *nf* state proposed by Pandiscia and Schweitzer-Stenner is membrane-bound [48]. However, the binding data of both groups suggest that the fraction of bound proteins should be close to 1 at the lipid/protein ratio used for the FRET experiments.

**Figure 19:**
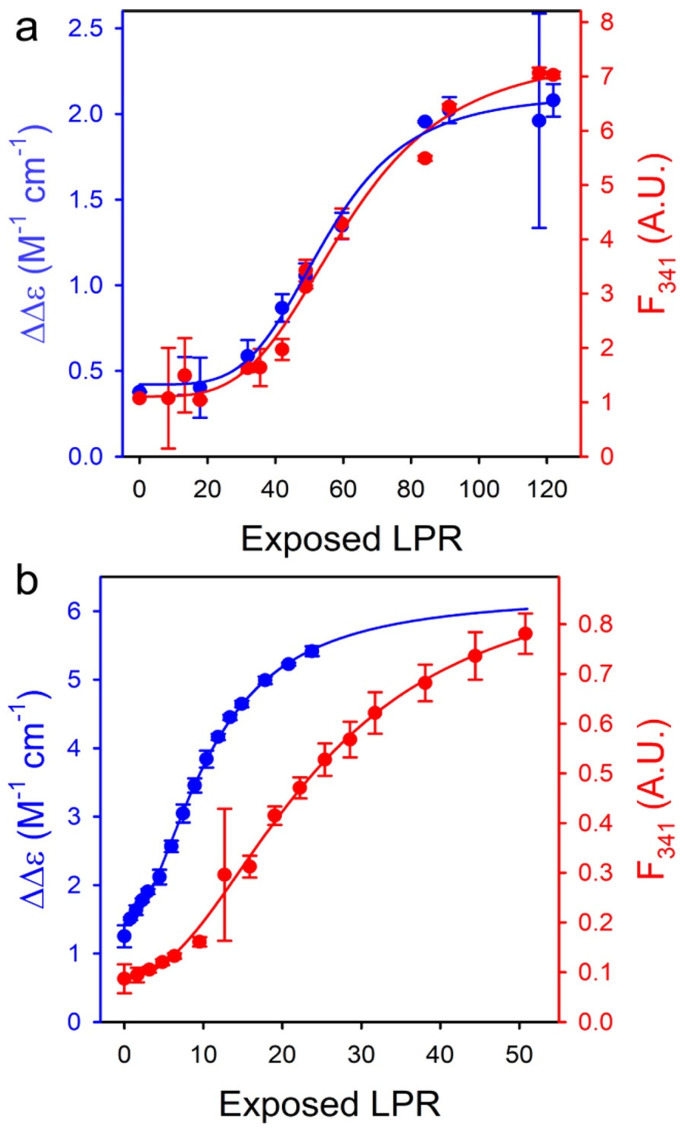
Binding isotherms for oxidized yeast iso-1-cytochrome *c* interacting with the (**a**) inner concave surface and (**b**) the outer convex surface of 100% CL LUVs. Average Soret band CD amplitude (ΔΔε = Δε _406_ − Δε_420_, solid blue circles) and average W59 fluorescence emission at 341 nm (F341, solid red circles) are plotted as a function of the respective exposed lipid-to-protein ratios. Solid curves resulting from a fit of Equation (1). Taken from ref. [63] with permission. American Chemical Society, 2020.

Elmer-Dixon et al. used Soret band CD and W59 fluorescence to explore differences between the binding of oxidized cytochrome *c* to the inner and outer leaflets of 100% TOCL LUVs [63]. The experiments were performed at pH 8 to minimize the influence of L-site binding. As it has been common practice in this research group, they used yeast-*iso1*-cytochrome *c* for their experiment. Let us look at Figure 19, which shows the change in dichroism and fluorescence as a function of exposed lipid-to-protein ratio. As already reported in an earlier study from this group (*vide supra*), the apparent dissociation constants derived from the two data sets are different for the binding to the outer convex surface, but they are very similar (though not identical) for the binding to the inner concave surface. The results of the fits of the Hill model to these sigmoidal curves are listed in Table 2. The readers should be reminded once again that the recorded spectral changes report conformational changes caused by the binding process, not binding itself. The apparent affinity (inverse of the listed *K_d_* values) for the CD-monitored binding to the inner leaflet is nearly fivefold that of the one to the outer surface. For the fluorescence change, the effective binding is only enhanced 2.6-fold. If one interprets these results in the framework of the two-step model of Pandiscia and Schweitzer-Stenner [48], the data indicate a significantly increased affinity for the first binding step as well a decrease in the critical lipid-to-protein ratio for the *nf→f* transition on the concave inner surface. A more detailed and specific assessment would require a direct application of the two-step model in fits to the data of Elmer-Dixon et al. This task is outstanding.

The disappearance of the 695 nm charge transfer band at high cardiolipin-to-protein ratios (Figure 9 and Figure 11) clearly shows that the M80 is no longer an axial ligand of the heme iron. Since resonance Raman and optical spectroscopy data clearly reveal a hexacoordinate low-spin state [54,72], only lysine or histidine appear as proper M80 replacement. Resonance Raman work on cardiolipin-bound cytochrome *c* has provided considerable evidence for the notion that M80 is replaced by a histidine residue [73]. This notion is consistent with the population of high-spin states at pH 6.5, which require a dissociation of a histidine caused by the protonation of the coordinated imidazole group.

Finally, I briefly discuss the results of a rather different conformational study of cardiolipin-bound cytochrome *c* by two-dimensional NMR. Mandal et al. used solid-state NMR to study ^13^C- and ^15^N-labeled oxidized and reduced cytochrome *c* bound to LUVs formed with a mixture of 25 mol% TOCl and 75 mol% DOPC [74]. Technical details of this study can be inferred from their paper, and a summary is given in an earlier review [6]. Here, I focus on the obtained results and the conclusions drawn by the authors. Their data reveal that membrane-bound cytochrome *c* is less mobile than the alkyl chains of TOCL, which suggests a rather compact and inflexible protein structure. The bound proteins do not impede the mobility of the lipid chains. Magic angle spinning NMR spectroscopy did not reveal any major structural disorder. The authors concluded that cytochrome *c* binding to TOCL does not involve any major structural changes and that the observed peroxidase should be due to local dynamics of the active site. Such a result would be at variance with nearly all the binding and structural studies thus far discussed in this and preceding sections. However, a closer look at the experimental conditions of the Mandal et al. study resolves some of the contradictions. The chosen lipid/protein ratio of 10 would correspond to a total TOCL/protein ratio of 5 if TOCL were equally partitioned between the inner and outer leaflets of the formed vesicles. In view of the above results of Elmer-Dixon et al. [63], it is more likely that the actual ratio is 2 for the outer membrane. That is a very low value. If cytochrome *c* binding to TOCL causes lipid–lipid demixing of TOCL and DOPC (*vide infra*), molecular crowding can be expected to keep the protein in the native-like state, as observed by Mandal et al. However, the obtained peroxidase activity of the protein remains somewhat mysterious and requires further studies.

**Figure 20:**
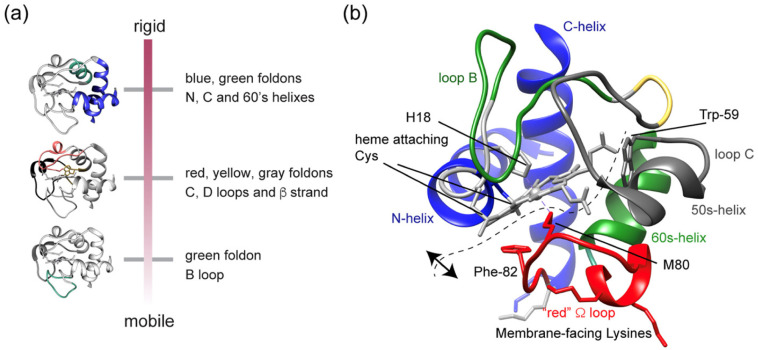
(**a**) Representation of hierarchical dynamics of cytochrome *c* bound to cardiolipin-containing membranes as deduced from solid-state NMR spectra. (**b**) Molecular structure of cytochrome *c* highlighting key residues and the so-called foldons proposed by Englander and coworkers [68,76]. The dashed line indicates the hypothesized opening of the CL-bound protein fold (indicated by the arrows) such that it would preserve the stable blue foldons with associated heme (top/left), while increasing the spacing between heme and residues M80, F82, and W59 (right/bottom), as detected spectroscopically. The range of motion depends on the experimental conditions (e.g., lipid composition). Taken from ref. [75] (open access).

In a more recent, more extended study, the same research group reported NMR and fluorescence studies that explored the structure and dynamics of TOCL-bound cytochrome *c* as a function of TOCL-to-lipid ratio [75]. The authors found that the dynamic flexibility of certain segments of the protein increased when this ratio was increased. They interpreted their results in terms of the foldon model of Englander and coworkers [68,76]. This model subdivides the protein into different segments called foldons that differ in terms of thermodynamic stability. These foldons are labeled with different colors (Figure 20). The blue foldon that encompasses the N- and C-terminal helices is the most stable one. The red Ω loop that contains the distal M80 ligand is more likely subject to unfolding in the presence of denaturing agents. Li et al. characterized the foldons of TOCL-bound cytochrome *c* in terms of flexibility. Their result is visualized in Figure 20. Contrary to the situation in the native cytochrome *c*, parts of the green foldon, namely, the loop labeled B and the C and D loops in the gray foldon, are the most flexible, thus exceeding the dynamics of the Ω loop in the red foldon. The relative rigidity of this foldon can be related to the fact that it encompasses the A-binding site of the protein. An increasing flexibility of the C loop could also involve W59. According to this model, it would stay just farther away from the heme for a certain period of time, which could reduce the average quenching of the fluorescence. Unfortunately, the authors did not make an attempt to use their very intriguing data set for a comparison with the results of the Pletneva, Bowler, and Schweitzer-Stenner groups. Just an inspection of Figure 20 seems to suggest that even the most flexible version of the protein at high lipid concentrations is clearly more compact than the one Pletneva and coworkers derived from their site-specific FRET data. It is unclear whether or not the Fe^3+^-M80 is broken at any point in the protein dynamics. The rupture of this coordination has clearly been documented by the loss of the 695 nm charge transfer band [48]. It is also unclear to which extent the model of Li et al. would encompass the replacement of M80 by a histidine ligand.

Two more recent studies that both corroborate partial unfolding of oxidized cytochrome *c* on anionic membranes should be mentioned in this context. A time-dependent NMR study by Zhan et al. [77] explored cytochrome *c* located in the inner membrane space and on the inner mitochondrial membrane. They used chemical shift changes as indicators of structural changes. Their results strongly suggest the structure of the protein bound to the inner membrane surface is non-native, thus corroborating the above solution studies with model liposomes. Paul et al. measured excited state (W59) fluorescence and femtosecond transient absorption spectra of oxidized cytochrome *c* in the presence of cardiolipins with different numbers of double bonds in their alkyl chains [78]. They observed enhanced fluorescence intensity and an increase in the respective excited-state lifetime with increasing numbers of double bonds. One might wonder whether this result is actually supporting the lipid anchorage model, since the X-ray structure of domain-swapped dimers suggests that W59 could be in close proximity to any inserted hydrocarbon chain [50].

Taken together, most of the spectroscopic studies reviewed in this section show that oxidized cytochrome *c* undergoes a conformational change on the surface of cardiolipin-containing vesicles. This is in line with multiple studies by Hildebrand and coworkers in the nineties, who investigated the binding of cytochrome *c* to solid anionic surfaces with resonance Raman spectroscopy [79,80,81,82]. The thus-produced partial unfolding of the protein enhanced the contact between the protein and the vesicle surface. It was in thermodynamic equilibrium, with a more compact and native-like state that still exhibited M80 as axial ligands. Several lines of evidence suggest that this equilibrium depends on the surface density of bound proteins. The partially unfolded and more extended structure becomes more populated at high cardiolipin-to-protein ratios, where the protein density is lower and molecular crowding effects inoperative. A similar model emerged from the resonance Raman study of Oellerich et al., who explored the binding of cytochrome *c* to DOPG phospholipid membranes (Figure 6 of their paper) [82]. While the fluorescence and Soret band CD data discussed above were interpreted in terms of a two-state model, the data of Li et al. seem to suggest a single state, the potential minimum of which broadens and shifts with increasing lipid concentrations [75]. None of the work referenced in this section suggests massive lipid insertion. As a matter of fact, the high degree of cardiolipin flexibility reported by Mandal et al. rules out this possibility [74].

### 3.3. Membrane Lipid–Lipid Demixing and Protein Penetration

Thus far, the interactions between cytochrome *c* and cardiolipin-containing vesicles have been treated as a binding to surfaces. The analysis of Muenzner et al. [71] and Grobenko et al. [32] yielded some membrane penetration of segments of the partially unfolded protein into the membrane, but they did not indicate any major change in the surface shape, which could encompass the local formation of concave shapes or even the formation of holes that would allow the protein to move into the interior of vesicles. Hints in this direction had emerged from the early work of De Kruijff and Cullis [83], who reported the formation of a hexagonal membrane phase upon cytochrome *c* binding. Heimburg et al. reported a similar observation for the binding of oxidized cytochrome *c* to vesicles formed with DOG and DOPG [28]. Results from molecular dynamic simulations suggest that binding-induced cardiolipin clustering induces a negative curvature on the membrane surface [84].

On the contrary, the paper of Mandal et al. cited above states that they found no evidence for a membrane penetration by cytochrome *c* [74].

**Figure 21:**
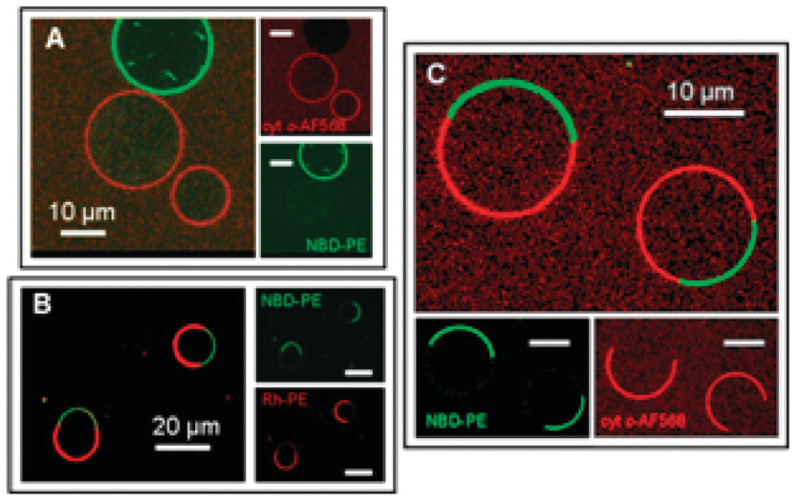
Fluorescence microscopy images of vesicles with and without cytochrome *c*. (**A**) DOPC vesicles (green) coexisting with 80 mol% DOPC/20 mol% bovine heart CL vesicles (unlabeled) and Alexa Fluor 568 labeled ferricytochrome *c* (red). The protein binds solely to the CL-containing vesicles. (**B**) Phase-separated vesicles containing 10 mol% bovine heart CL, 25 mol% cholesterol, 27.5 mol% DOPC, and 37.5% mol% DPPC (0.5 mol% Rh-PE, 0.5 mol% NBD-PE). The Rh-PE (red) visualizes the L_d_ phase, and the NBD-PE (green) labels the L_o_ phase. (**C**) Phase-separated CL-containing vesicles (green) in solution with cytochrome *c* (red). The protein binds only to the CL-containing L_d_ domains. The insets represent the separate red and green fluorescence channels that were collected and superimposed to create the composite image. Taken from ref. [85] with permission. American Chemical Society, 2011.

The most convincing evidence for cytochrome *c* penetration came from microscopic studies of Groves and coworkers, who explored the binding of oxidized cytochrome to so-called gigantic unilamellar lipid vesicles (GUVs). They compared images of fluorescence-labeled cytochrome *c* (Alex Fluor 568) in the presence of DOPC vesicles (labeled with NBD-PE, i.e., N(7-nitrobenzene-2-oxa-1,3-diazol-4-yl-1,2-dihexadecanoyl-*sn*-glycero-3-phosphothanolamine, trimethylammonium salt) and of unlabeled 75 mol% DOPC/25 mol% TOCL. As shown in Figure 21A, the labeled cytochrome *c* molecules bound solely to the bovine heart cardiolipin-containing vesicles, as expected. The images in Figure 21B,C reveal how changes in the membrane phase are involved in cytochrome *c* binding. The authors used the well-known fact that the phase diagram of ternary mixtures of DOPC–DPPC–cholesterol have a large region with coexisting liquid-ordered (L_o_) and lipid-disordered domains (L_d_). In Figure 21B, the two phases are labeled differently: NBD-PE (green) was used for the L_o_ phase, while Rh-RE (red, rhodamine B 1,2-dihexadecanoyl-*sn*-glycer-3-phosphoethanolamine, and trimethylammonium salt) was used to label the L_d_. Figure 21C shows the result of a replacement of 10 mol% DOPC by cardiolipin. The addition of cytochrome *c* clearly shows that it binds only to targets in the L_d_ domain, to which all the cardiolipins have migrated.

**Figure 22:**
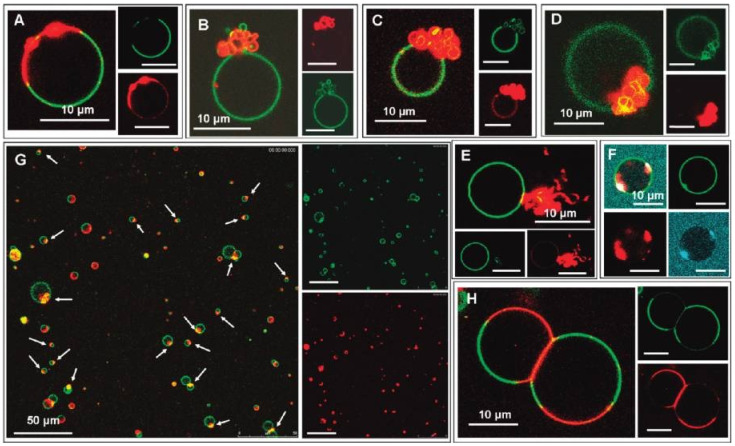
Fluorescence microscopy images of phase-separated CL-containing GUVs labeled by NBD-PE (green) and Rh-PE (red) probes. Images (**A**–**E**,**G**,**H**) depict GUVs interacting with cytochrome *c* (red); image (**F**) shows a GUV in the presence of cytochrome *c* (blue). (**A**) Beading of the membrane in the L_d_ domain. (**B**–**D**) The L_d_ domains on the vesicles have collapsed into a folded structure. The folds of the membrane are resolvable under confocal microscopy. (**E**) The morphology of the L_d_ domain after cytochrome *c*-induced collapse exhibits a “wispy” morphology. (**F**) Collapse of two separate L_d_ domains coexisting in the same vesicle. (**G**) Lower-magnification image of multiple GUVs in the sample. White arrows indicate L_d_ domains that have collapsed to a folded structure after protein addition. (**H**) Strong adhesion between the negatively charged, CL-containing L_d_ domains induced by the polycationic cytochrome *c* is shown to result in membrane deformation and a large osculating area between the two GUVs. The insets depict separate fluorescence channels that are collected and superimposed to create the composite image. Taken from ref. [86] with permission. American Chemical Society 2010.

While the above experiments reveal the influence of cytochrome *c* binding on lipid–lipid demixing, Figure 22 shows images that reveal the dynamic behavior of the vesicle-protein complexes subsequent to protein binding. The images in Figure 22A–E reveal the transformation of so-called buds comprised of cytochrome *c*–cardiolipin complexes into a single, rather gigantic bud. Figure 22F shows that two L_d_ domains on the same vesicle collapse into what the authors called tight-folded structures (compared with the “wispy” structure of the gigantic bud in Figure 22E). Figure 22H shows the fusion of the two vesicles. Note here the different labels of cytochrome *c* with Alexa Fluor 568 (red) and Alexa Fluor 633 (green).

It should be noted that Beales et al. found that other cationic proteins (lysozyme, horse heart cytochrome *c*, α-synuclein (*vide infra*), and different types of dendrimers) have a similar impact to yeast cytochrome *c* on the employed GUVs. This and the observed inhibition of the process at comparatively high NaCl concentrations (390 and 780 mM) suggest that the involved binding of the proteins is entirely electrostatic in nature.

**Figure 23:**
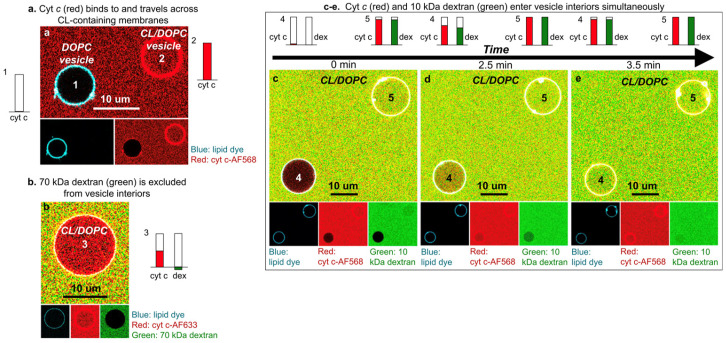
Microscopy images depicting the cytochrome *c*-induced permeabilization of CL-containing membranes of GUVs. (**a**) DOPC with NBD-PE (left vesicle) and 80 mol% DOPC/20 mol% CL (right vesicle) treated with Alexa Fluor 568–cytochrome *c* in solution (red). (**b**) The above GUV with NBD-PE, mixed with fluorescent 70 kDa dextran (green) and Alexa Fluor 633–cytochrome *c* in solution (red). (**c**–**e**) Time series of above GUVs with NBD-PE treated with fluorescent 10 kDa dextran (green) and Alexa Fluor 633–cytochrome *c* in solution (red). In all images, the cyan lipid dye is 0.5 mol% NBD-PE. Average normalized protein and dextran concentrations for each vesicle interior vs. background are plotted on the top of the right figure. Taken from ref. [86] with permission. National Academy of the Sciences USA 2013.

I now move to another study of this research group, where Bergstrom et al. [86] took images of cardiolipin-containing GUVs mixed with fluorescently labeled neutrally charged 10 kDa dextran in the presence and absence of cytochrome *c*. Dextran was used as an indicator of membrane pores. The images in Figure 23 show that dextran penetrates the investigated vesicle solely in the presence of cytochrome *c*. In addition, cytochrome *c* itself penetrates the membrane (cf. Figure 23a,c–e).

While the results reported in these studies, which were later confirmed by other experiments (cf. the combined use of optical trapping and resonance Raman spectroscopy by Kitt et al.) [87], are outright beautiful and convincing owing to their direct character, they leave a lot of questions open. It is difficult to imagine that the observed budding of domains and the subsequent penetration through formed pores of the membrane is reversible on any experimentally accessible time scale. Thus, these results appear to be at variance with all the binding and structural studies outlined above. Even those studies from which authors deduced some membrane penetration of the protein cannot be brought in line with the coagulation and vesicle insertion described above. The pH of the imaging studies was 7.4. Hence, binding experiments of the Kinnunen, Bowler, and Schweitzer-Stenner groups can be directly related to these studies. One might argue that the use of GUVs facilitates pore formation and the observed phase separation. However, the authors report that similar effects occur with smaller vesicles. A closer look at the time scale of the budding and penetration process might point us to a resolution of the conflict. The kinetic data of Beales et al. [85] and Bergstrom et al. [86] suggest that their onset occurs only after a significant waiting time that lasts at least 10 min. That exceeds the time it normally takes to measure a fluorescence or CD spectrum after the addition to cardiolipin-containing vesicles. Since data points taken with different lipid/protein ratios are generally taken with different samples, the incubation time remains more or less constant. This argument is of course only valid for experiments where binding is probed with varying lipid concentrations. However, if one titrates cytochrome *c* to a fixed amount of lipids, the experiment might eventually move into a time regime where the vesicle morphology changes, as revealed by the above images. The lack of protein dissociation of proteins from fluorescently labeled cardiolipin-containing vesicles in the presence of unlabeled vesicles might point in this direction. However, if incubation time is a factor, one wonders why the NMR studies of Mandal et al. [74] did not provide any hint towards protein penetration or lipid phase transition. The answer might lie in the low-temperature regime in which these studies were conducted (236–271 K). It is certainly conceivable that the phase-separation and pore-forming processes would not occur in such conditions.

### 3.4. Conformational Change and Peroxidase Activity

In its native state, cytochrome *c* is not a peroxidase. If one takes horseradish peroxidase as a representative heme protein for this type of enzymatic activity, one expects a resting state with a pentacoordinate high-spin state Fe^3+^ state [88]. A water molecule resides close to the heme iron without providing coordination. Such a state enables the formation of the ferryl compound I and II intermediates in which Fe^4+^ is coordinated with an oxygen [89]. Based on this information, one is tempted to expect that only the high-spin state of membrane-bound cytochrome *c* observed at acidic pH would be a suitable candidate. However, the literature presented below reveals a significantly different picture.

**Figure 24:**
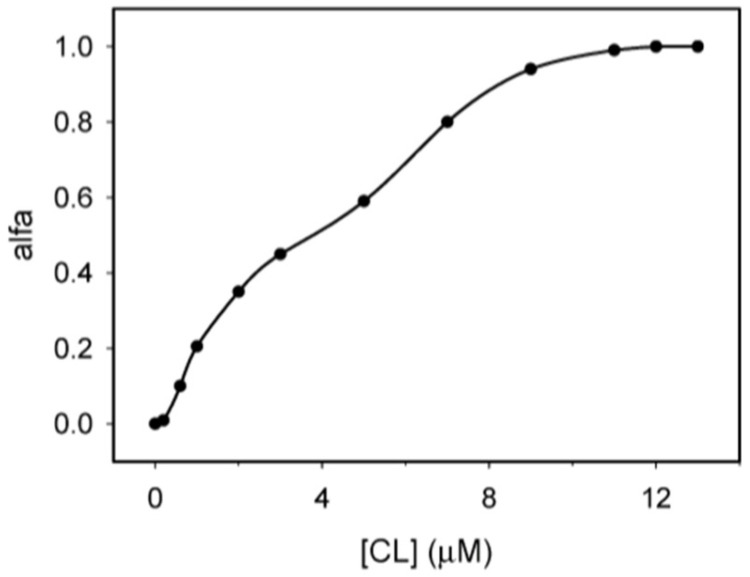
Peroxidase activity of oxidized cytochrome *c* as a function of bovine heart CL concentration (100 mol% CL liposomes) probed by the formation of tetra-guaiacol. The protein concentration was 1 µM. Note that the peroxidation data are plotted as a fraction of the activity plateau value.

A comparison of the above two-state models with the early work of Belikova et al. [90] suggests that the peroxidase activity of horse heart cytochrome *c* should be associated more with the extended state, while the electron transfer capability requires a folded protein with the M80 ligation intact. The thus proposed structure–function relationship is in line with the obtained correlation between the population of the E state at different NaCl concentrations and initial rate of the peroxidase reaction (cf. Figure 4 in ref. [70]). However, available experimental data defy the expectation of an unambiguous relationship between structure and function of membrane-bound cytochrome *c*. Figure 24 shows the (relative) peroxidase activity of oxidized (horse heart) cytochrome *c* bound to 100% beef cardiolipin membranes reported by Sinibaldi et al. [47]. The data cover a broader region of lipid/protein ratios than their binding studies (note that they used 10 μM cytochrome for the latter and 1 μM for the activity measurement). However, the data are still mostly in a region with high protein density. Nevertheless, the authors obtained two phases of increasing peroxidase activity that seem to correspond to the ones observed for the binding isotherms (Figure 10). The logical conclusion from these data would be that the C/*nf*-state already has peroxidase activity.

**Figure 25:**
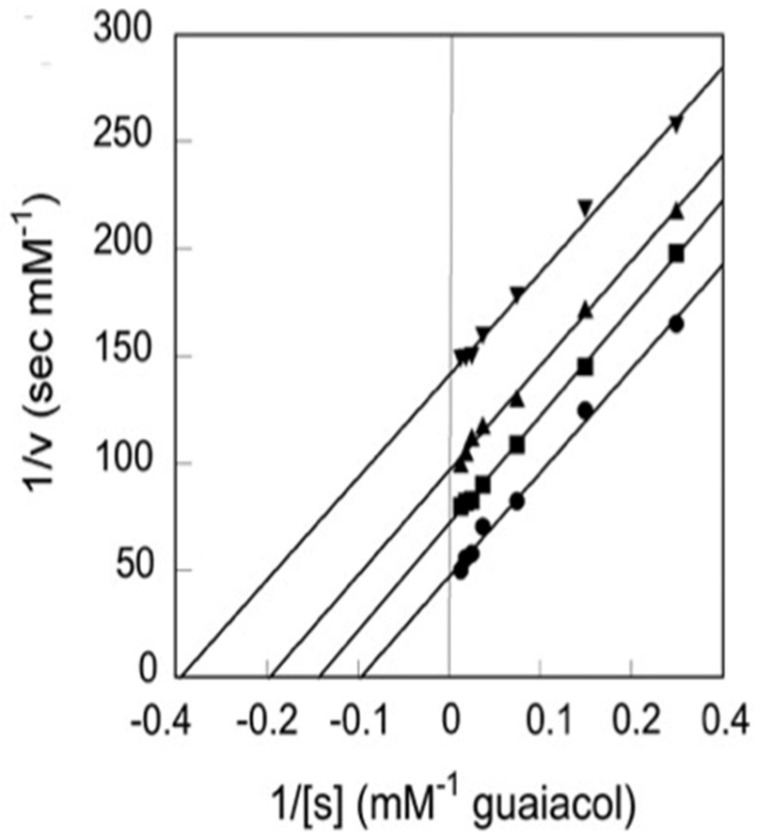
Representation of the inhibitor effect of minocycline on peroxidase activity of different forms of cytochrome *c* in the presence of 50 mol% POPC–50 mol% bovine heart CL determined by probing the oxidation of guaiacol at pH 7 and room temperature. Minocycline concentrations of 0 (filled circle), 5 (filled quadrants), 10 (triangles pointing downwards), and 20 μM (triangles pointing upwards) were added to the sample. Lineweaver–Burk plots show the uncompetitive inhibition of the peroxidase activity for the cytochrome *c*–CL complex. Reaction conditions can be inferred from ref. [91], from where the figure was taken with permission. Elsevier 2012.

A more quantitative and systematic assessment of the peroxidase activity of cardiolipin-bound horse heart cytochrome *c* was carried out by Patriarca et al. [91]. Their work focused on the inhibitory mechanism by minocycline. The protein was allowed to react with liposomes formed with 100% bovine heart cardiolipin. The peroxidation activity was probed by the oxidation of guaiacol in the presence of H_2_O. The formed liposomes lay in a region between SUVs and LUVs (50 nm). Figure 25 shows Lineweaver–Burk plots of the protein measured without and with different concentrations of the inhibitor. The data suggest uncompetitive inhibition. In the present context, I note that the experiment was carried out at pH 7.0 with protein and outer leaflet lipid concentration of 1 and 40 μM, respectively. Based on the data of Pandiscia and Schweitzer-Stenner [48], this puts the system into the realm of E/f—state population. The authors obtained Michaelis Menton parameters of *K_m_* = 23 μM and *k_cat_* = 0.014 s^−1^. Hence, the protein is by orders of magnitude less effective compared with horseradish peroxidase (3300 μM and 562 s^−1^). This observation is consistent with the suboptimal conditions of heme ligation.

**Figure 26:**
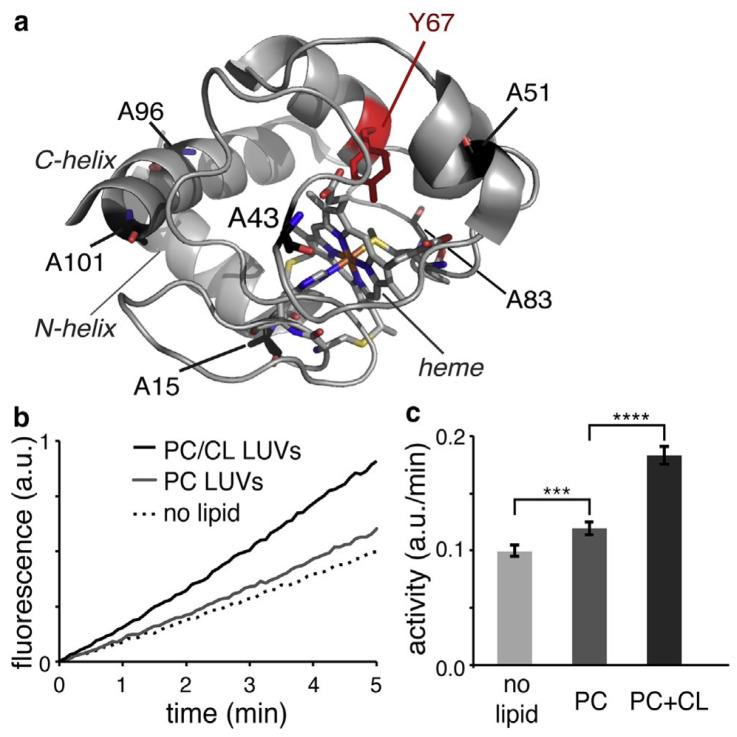
Demonstration of cytochrome *c* structure and peroxidase activity. (**a**) X-ray structure of oxidized cytochrome *c*. Labels mark the catalytic sites of peroxidase–active protein (heme and Tyr-67) and six alanine residues which Mandel et al. used as site-specific reporters for cytochrome *c* binding. (**b**) Fluorescence emission intensity probing the oxidation product resorufin in the presence of 0.5 mM cytochrome *c* andf the indicated liposomes. Measurements were performed at room temperature in the absence (dashed line) and presence (solid gray line) of DOPC LUVs or 80 mol% DOPC/20 mol% TOCL LUVs (black solid line). (**c**) Relative peroxidation rates obtained from linear fits to the data in (**b**). *** *p* < 0.001; **** *p* < 0.0001. Taken from ref. [74] with permission. Elsevier 2015.

Mandal et al. reported relative peroxidase rates probed by the fluorescing oxidation product resorufin. They compared the activity of horse heart cytochrome *c* in the absence and presence of DOPC and vesicles with 20 mol% TOCL/80 mol% DOPG (Figure 26). Note that the experiments of these researchers were carried out at low lipid-to-protein ratios. Nevertheless, the addition of cardiolipin was found to increase peroxidase activity. Mohammadyani et al. combined NMR spectroscopy with measurements of the initial rate of peroxidase reactions of cardiolipin-bound horse heart cytochrome *c* at different pH (7.4 and 6.0) [84]. From their NMR data, they identified three different binding sites that overlapped with the canonical A, C, and L sites, but involved more residues than originally suggested. They found that the cardiolipin-to-cytochrome *c* ratio has to exceed a threshold of 6 to facilitate peroxidase activity. This number is still very low compared with cardiolipin-to-protein ratios required for a substantial *E*- and/or *f*-state populations. The authors proposed a scenario in which A- and L-state binding (called distal and proximal sites in their paper) act simultaneously to facilitate peroxidase activity by opening the heme crevice for substrates. Two problems with their approach and the proposed model should be mentioned. First, some of their studies were performed in 50 mM phosphate buffer. HEPES buffer was only used for the pH 7.4 peroxidase measurements. This seems to be problematic in view of the observation that phosphate binding to cytochrome *c* could compete with cardiolipin binding and modify the structure of the heme environment of the proteins in solution [92]. Second, the binding data observed at pH 7.4 [48,50] described above are not consistent with pure electrostatic binding, as opposed to results obtained at pH 6.5 [54].

Very recent preliminary investigations in our own laboratory were aimed at probing the peroxidase activity of oxidized horse heart cytochrome *c* bound to 20 mol% TOCL/80 mol% DOPC with the above guaiacol substrate at (nearly neutral) pH 7.4 and 6.5 [93]. Based on the results of Milorey et al. [54] one could expect that the population of the penta/hexacoordinate high-spin state at acidic pH would produce higher peroxidase activity. However, the obtained Michaelis–Menton plots were nearly identical. At that point, we investigated whether or not the addition of the substrate might affect the structure of the protein at pH 6.5. Obtained Soret band CD spectra suggest that this indeed happens at intermediate lipid-to-protein ratios.

In lieu of a clear picture of the peroxidase-related structure–function relationship, attempts have been made to produce peroxidase-active mutants of cytochrome *c* in solution. McClelland et al. reported the X-ray structure and the peroxidase activity of a yeast *iso-1*-cytochrome *c* mutant in which trimethyllysine 72 was replaced by an alanine [94]. In this protein, the M80 ligand is replaced by a water molecule. The structure exhibits a water channel that can be expected to facilitate the access of H_2_O_2_ to the active site. Interestingly, they found that an increase in the *k_cat_* value (relative to the trimethylated wild type) occurred only at pH 7 and higher. The highest value was obtained at pH 7.5 (ca. 3.5 s^−1^). Since the authors used yeast rather than horse heart cytochrome *c*, this value is significantly higher than those reported by Patriarca et al., but still significantly below the horseradish peroxidase value [91]. As already mentioned above, the work of Steele et al. [50] suggests domain-swapped dimers as the possible source of peroxidase activity, which could include the insertion of a cardiolipin chain into the formed channel [67].

### 3.5. Summary

In spite of more than thirty years of research, an absolutely clear and consistent picture of cytochrome *c* binding to cardiolipin-containing membranes has not yet emerged. Let us start with what seems to be clear. Upon binding of cytochrome *c* to cardiolipin, the latter demixes from the rest of the membrane. The bound protein fluctuates between extended and compact conformations. Whether or not the two represent two minima in the Gibbs energy landscape or just the occupation of different energies in a flexible potential that broadens with increasing lipid-to-protein ratios might need further clarification. However, kinetic data reported by Muenzner et al. indicate that the transition from C to E proceeds more quickly than 5 ms [71]. This might be too fast for NMR to isolate the contributions from these states [95]. Obviously, the extended form has the M80 ligand replaced by the imidazole group of a histidine at neutral and very mildly acidic pH (<6.5). The extended form must be the prime candidate for peroxidase activities. Moreover, it is obvious that the protein possesses different binding sites, but the picture derived from the canonical Kinnunen model (A and C sites) seem to be incomplete [39]. The C site was proposed as becoming relevant at pH values that permit one of the phosphate groups of the cardiolipin head group to be protonated. However, several lines of experimental evidence suggest that this does not occur at neutral or even mildly acidic pH, irrespective of the CL-content of the vesicles used [72,96,97]. A-site binding was suggested to be electrostatic in nature owing to the presence of several protonated lysine residues. However, fluorescence and optical spectroscopy experiments suggest that only the second binding step, namely, the conversion of a compact into an extended structure of the protein, is governed by electrostatics. At neutral pH, the first step is not affected by the presence of, e.g., NaCl. This observation is at variance with predictions derived from the electrostatic theory of Heimburg and Marsh [27]. Interestingly, the latter seems to work for cytochrome *c* binding to DOPG-containing membranes. However, at acidic pH, where the L-site binding proposed by Nantes and coworkers is operative [52,53], this theory nicely explains the observed inhibition of cytochrome *c* binding. The results of the imaging studies described in Section 3.3 suggest the onset of significant morphological changes cardiolipin-containing vesicles after an incubation time of at least 10 min. Are these changes biologically relevant for mitochondrial membranes, or would one expect that the presence of membrane proteins would prevent this from occurring? If it occurs, does it happen prior to or after the peroxidase activity of the protein? With regard to the binding sites, the question arises as to which of them is actually physiologically relevant. The mitochondrial pH is generally around 6.7, which would clearly favor L-site binding. The peroxidase activity associated with it has still to be determined. Preliminary results have been inconclusive. Finally, I wonder whether the preference of cytochrome *c* binding to convex surfaces is indeed an indication of a preferred binding to cardiolipin in *cristae*. In consideration of all of this, the story of the multifunctional protein named cytochrome *c* has not yet come to an end.

## 4. α-Synuclein–Phospholipid Binding

### 4.1. α-Synuclein in Solution

With a few exceptions, experimental evidence suggests that α-synuclein is monomeric and intrinsically disordered in solution. Its state is often characterized as a random coil. While Kratky plots obtained from small-angle X-ray scattering data indicate that this notion may be true globally (if the term random coil means a self-avoiding coil), the site-specific secondary chemical shifts of ^13^C, ^15^N, and ^1^H signals reveal that it does not apply locally [98]. An analysis of the chemical shift data revealed that the population of polyproline II conformations is elevated over what the authors considered random coil values in various regions of the protein, particularly in the 76–81 region. Similar observations were reported for tau, another intrinsically disordered protein involved in amylogenesis. The authors used chemical shift values of amino acid residues in short peptides as reference for local random coil distributions. However, several lines of experimental and computational evidence suggest that most of these residues exhibit structural preferences for either polyproline II or extended β-strand conformations, which deviates from the classical views of local random coil behavior [99]. Overall, it is more appropriate to use the term “statistical coil” for the disordered state of α-synuclein [100,101].

The amino acid sequence of α-synuclein is generally subdivided into the three domains exhibited in Figure 2. While the N-terminal has a positive net charge, which makes it suitable for electrostatic interactions with anionic phospholipid membranes, the C-terminal segment carries a negative charge, which should in principle oppose such interactions. The NAC domain is hydrophobic and could add van der Waals interactions with lipid chains to the binding energy. In the two subsequent sections, I first sketch some aspects of α-synuclein binding to different types of phospholipid membranes. In the second section, I briefly delineate the conditions that must be met for the protein to self-assemble into oligomers and fibrils on the surface of anionic lipid membranes. Differences and similarities between cytochrome *c* and α-synuclein binding are described. I would like to emphasize that the following sections are not designed as a comprehensive review of all aspects of α-synuclein–membrane interactions. Interested readers are referred to already existing reviews [8,102].

### 4.2. Binding and Structural Changes

**Figure 27:**
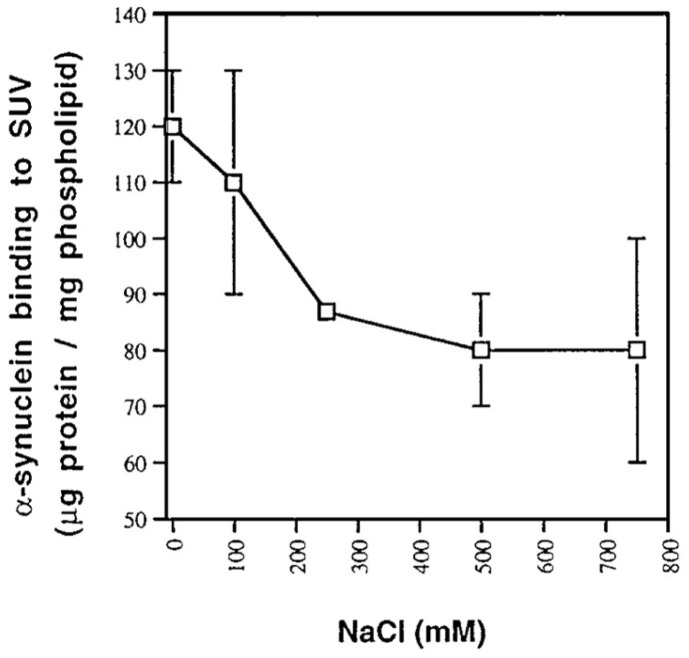
Ionic strength dependence of α-synuclein binding to SUVs composed of 50 mol% POPC–50 mol% POPA plotted as a function of NaCl. Technical details can be obtained from ref. [103], from where the figure was taken (open access).

I start this section by a brief description of the earlier binding study of Davidson et al. [103]. They used gel filtration to probe the binding of α-synuclein to SUVs and LUVs with palmitoleic and oleic fatty alkyl chains attached to the already introduced head groups PC and PS and also to phosphatidylethanolamine (PE) and phosphatic acid (PA). Experiments were carried out at pH 4. Table 3 lists the number of bound proteins obtained for SUVs and LUVs composed of 100% PC as well as of a 1:1 weight mixture of PC and PA. All these values were obtained with the same lipid-to-protein ratio. As one would expect, the admixture of PA increased the binding affinity significantly, which suggests the involvement of electrostatic interactions. The second remarkable observation was that SUVs bind the protein more efficiently than LUVs. However, the addition of NaCl causes only a partial inhibition of protein binding (Figure 27). This observation resembles the above findings for cytochrome *c* binding to cardiolipin membranes at pH 7.4. The authors measured the UVCD spectra of α-synuclein in solution and after incubation with various SUVs. If the latter was composed of a POPC and POPA mixture, the spectra indicated a switch from a statistical coil (with significant polyproline II content) mixture to α-helical conformation. The authors emphasized the significance of the differences between the binding to SUVs and LUVs by pointing out that with respect to size, the former are better model systems for vesicle function at the presynaptic terminal. It is noteworthy in this context that studies on quite a large number of intrinsically disordered proteins suggest an interplay between curvature and binding, in that the latter can actually induce (additional) curvature of the membrane in the vicinity of the respective binding site [104,105].

**Figure 28:**
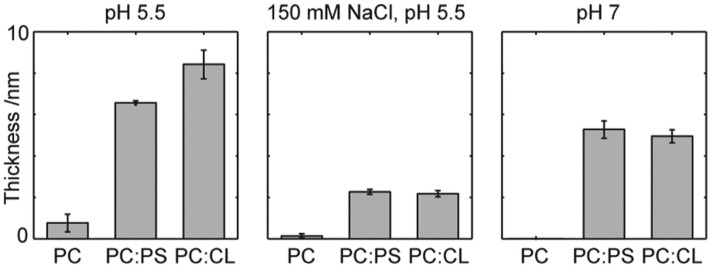
Thickness of α-synuclein film on the indicated lipid bilayers obtained from QCM-D data. Experimental conditions are indicated on top of the displayed graphs. Taken from ref. [106] with permission. American Chemical Society 2013.

Now, I move to a more recent work of Hellstrand et al. [106], who used quartz crystal microbalance (QCM) and neutron reflectivity to study the binding of α-synuclein to lipid bilayers. Such a setup differs from the experiments of Davidson et al. in that the formed membrane does not exhibit any curvature. The preferred binding to SUVs seems to indicate that curvature facilitates α-synuclein binding. The authors used 70 mol%/30 mol% POPC–DOPS and 85 mol%/15 mol% POPC–cardiolipin to examine protein binding at two pH values (5.5, 7.0). The results of their QCM studies are summarized in Figure 28, which depicts the thickness of the protein film for the indicated lipid membrane mixture. For pure PC membranes, the thickness is negligible, which suggests very limited binding. The much greater thickness of protein films on PC–PS and PC–CL membranes suggests again the involvement of electrostatic binding. Thickness values are a little bit reduced at pH 7 compared with the situation at pH 5. The addition of 150 mM NaCl reduced, but did not eliminate the protein film. In view of the fact that the net charge if α-synuclein was less than the one of cytochrome *c* at the investigated pH, the salt concentration used should have totally inhibited α-synuclein binding, if only electrostatic effects were operative (*vide supra*).

**Figure 29:**
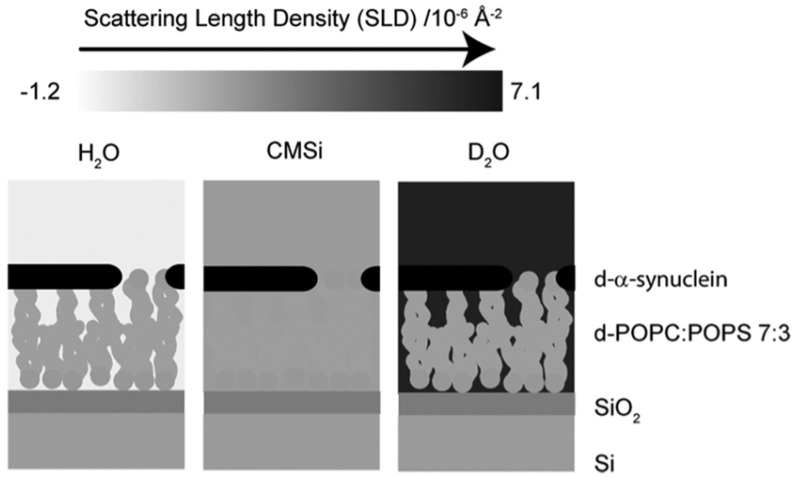
Illustration of the resulting SLD distribution in a 70 mol% d-POPC–30 mol% POPS bilayer with d-α-synuclein. Details are discussed in the text. Taken from ref. [106] with permission. American Chemical Society 2013.

The neutron reflectivity measurements aimed at exploring the penetration of the protein into the lipid membrane. To this end, they replaced all hydrogens of POPC and the protein with deuterium, since hydrogen and deuterium have different scattering length densities (SLDs). Figure 29 illustrates the obtained SLD distribution in a 7:3 d-POPC–POPS bilayer. The figure demonstrates how the different scattering lengths of deuterium and water produce a contrast in the respective images and illustrates the scenario that emerged from a very detailed analysis of the neutron reflectivity data, namely, a rather modest penetration into the head-group area of the membrane.

**Figure 30:**
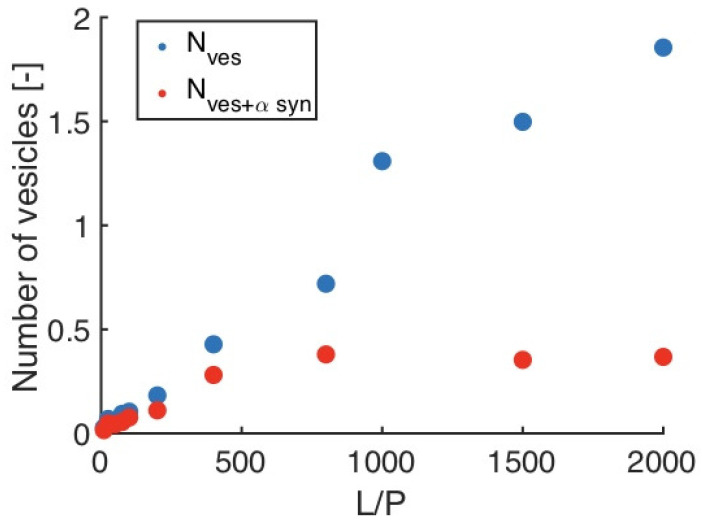
Distribution of *α*-synuclein in a population of small unilamellar vesicles (70 mol% DOPC–30 mol% DOPS) obtained with fluorescence correlation spectroscopy plotted as a function of the lipid-to-protein ration. Blue dots represent the total number of vesicles in the sample, while red dots represent the number of vesicles that are *α*-synuclein-bound. Taken from ref. [22] (open source).

**Figure 31:**
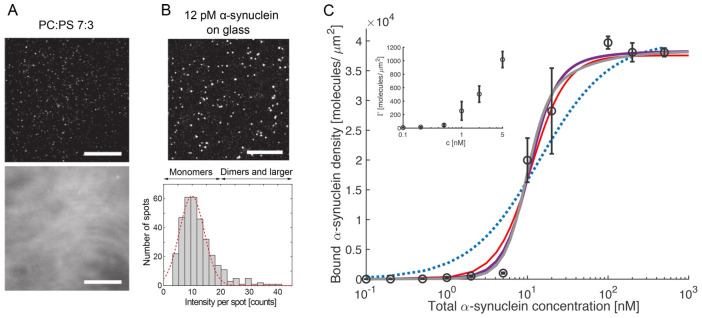
α-Synuclein binding to a flat supported lipid bilayer probed by fluorescence spectroscopy. (**A**) Fluorescence signal from a 70 mol% POPS–30 mol% DOPS bilayer (top). Protein concentrations were 0.1 nM α-synuclein and (bottom) 10 nM α-synuclein. (**B**) (Top) Single-molecule fluorescence image of 12 pM α-synuclein adsorbed on a bare glass slide. The scale bar is 20 μm for all images. (Bottom) Histogram depicting the total intensity per detected fluorescence “spot” in the single-molecule fluorescence image. The dashed red line is a Gaussian fit to the main peak, showing that 90% of the detected spots exhibit intensity of less than 20 units, corresponding to a monomeric form of the protein (*n* = 335). (**C**) Density of α-synuclein bound to the above bilayer plotted as a function of α-synuclein concentration as determined from the fluorescence signal. The dotted blue line shows a fit of the Adair equation for one binding site and corresponds to independent binding. The red, blue, and black lines represent fits of the Adair equation with two, three, and four coupled binding sites, respectively. Details of the analysis can be found in ref. [107], from where this figure has been taken (open source).

I now move to a very detailed study of α-synuclein binding to anionic lipid membranes by Makasewicz et al. [22,107]. These authors used fluorescence microscopy and fluorescence cross-correlation spectroscopy to investigate the binding of the protein to 70 mol% DOPC–30 mol% DOPS GUVs and SUVs, respectively. For the experiments with GUVs, the authors used an N122C mutant of the protein and attached Alex Fluor 647 maleimide as fluorescence label to the inserted cysteine residue. The trick of their investigation was that they used the brightfield mode of their microscope to observe GUVs without bound proteins and the fluorescence mode to identify vesicles with bound proteins. The result of their measurements is shown in Figure 30, where the number of vesicles with and without α-synuclein is plotted as a function of the lipid-to-protein ratio. While the respective numbers are nearly identical at very low ratios (high protein density on membranes), they diverge at high values in that only a very limited number of vesicles are occupied by proteins. To rule out the possibility that this discrepancy is related to the mixture of anionic and zwitterionic lipids employed, the authors repeated the experiment with 100% DOPS GUVs and observed the same effect. In order to rule out that the obtained effect was specific for GUVs, the authors performed fluorescence cross-correlation studies, for which they used SUVs with 0.5% green fluorescent lipid analogues added to the above lipid mixture. Technical details about their data analysis can be found in their paper. Here, I just present the obtained binding isotherms shown in Figure 31.

The authors interpreted all their data in terms of a cooperative model [22]. Positive cooperativity in the context of their system means that the first binding of a protein to a membrane surface increases the affinity of the second binding, which might increase the affinity of the third binding and so on and so forth. If the cooperativity is strong, the vesicles to which proteins are bound have a competitive advantage such that at the end, only a limited number of them become occupied by proteins. The authors analyzed their data with an Adair-type model [108], which has historically been used to explain the cooperativity of oxygen binding to hemoglobin prior to the use of the Monod, Wyman, and Changeaux model [109]. Fits with models that assumed independent binding and the existence of two, three, and four coupled binding sites are depicted in Figure 31 Apparently, the data cannot be used to distinguish between the last three options, but it is obvious that the independent site model does not work. The authors could not provide a mechanistic explanation of their findings. They speculated that initial binding might involve the creation of new hydrophobic interfaces.

**Figure 32:**
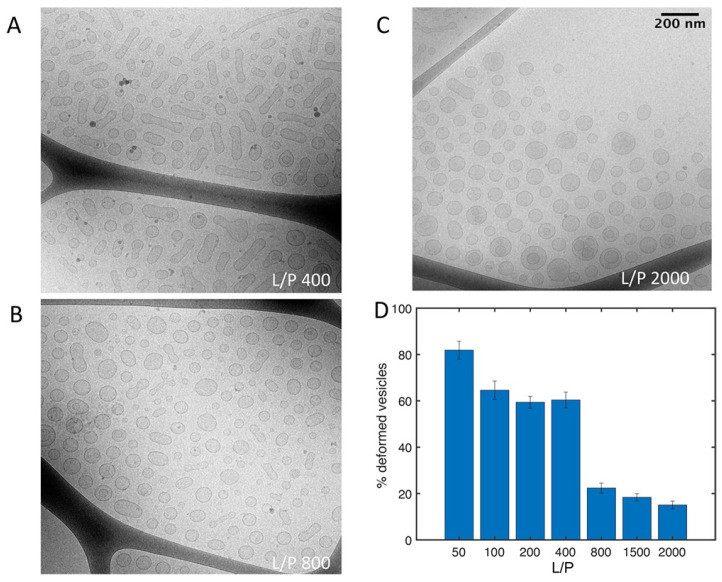
α-Synuclein binding to 70 mol% POPS–30 mol% DOPS SUVs studied with cryo-TEM. (**A**−**C**) Examples of cryo-TEM images of small unilamellar vesicles (SUVs) with α-synuclein at the indicated lipid/protein ratios. The scale bar is the same as in (**C**) for all images. (**D**) Percentage of deformed vesicles (means ± SD) calculated from six different images of each sample from one experiment assuming no fusion of vesicles. The number of analyzed vesicles was 5330. The figure was taken with permission from ref. [107] (open source).

Obviously, the results of the above study suggest that lipid binding of α-synuclein and cytochrome *c* are clearly different. While binding curves for the latter could be fitted with Hill equations, to the best of my knowledge of the literature, there are no experiments reported that suggest selective binding to just a few vesicles. Makasewicz et al. did not quantitatively compare binding to SUVs and GUVs, and hence the influence of curvature remained unaddressed in the above study. However, they discussed the issue in a later review, where they referred to a work by Braun et al., who used fluorescence correlation spectroscopy to probe the binding of a truncated α-synuclein (α-syn_100_, without the C-terminal) to 50 mol% POPG–50 mol% POPC and 100% POPG LUVs [110]. Their experimental work was combined with coarse-grain molecular dynamic simulations. The experimental results reveal that α-syn_100_ binding to the 100% POPG membranes caused turbulation. Binding to the surface of the PG–PC membrane had significantly lower affinity and did not cause any turbulation. The results of MD calculations suggested that the obtained discrepancies might reflect differences between the protein’s membrane penetration and its hydrophobic thickness. With regard to curvature, their MD analysis revealed an anisotropy of the induced local curvature, which leads to an ordering of proteins on the membrane. In another paper, Makasewicz et al. used cryo-transmission electron microscopy (TEM) to investigate SUVs with 70 mol% DOPC–30 mol% DOPS in the absence and presence of α-synuclein [107]. Figure 32 shows TEM images of the respective SUVs and a schematic drawing that visualizes the induced anisotropic curvature of the SUVs.

**Figure 33:**
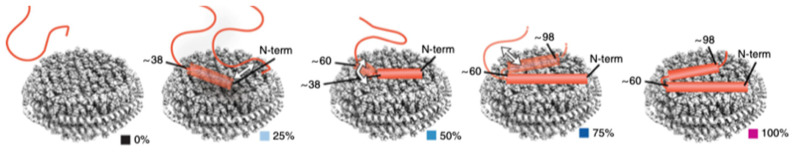
Representation of α-synuclein binding to anionic membranes fixed in a nanodisk based on the results of NMR experiments described in detail in ref. [111], from where the figure has been taken (open source). White arrows and transparent coloring indicate αS regions that fluctuate between multiple (dynamic or static) states.

A rather different binding study of Viennet et al. deserves consideration [111,112]. The authors used solid-state NMR to pinpoint the site specificity of α-synuclein binding to lipid-bilayer nanodisks. A nanodisk is a circular lipid membrane system surrounded and kept in place by amphipathic proteins. Its application in biophysical studies in particular heme proteins has been pioneered by Sligar and coworkers [112]. Its advantage is that it provides very well-defined conditions. The thus-produced membranes are very stable, and in the case of α-synuclein binding, not subject to disruption or curvature formation. The apparent disadvantage is that this is not an ideal model for biological membranes. Nevertheless, the results of Viennet et al. are likely to be very useful for an understanding of the binding of α-synuclein to biological membranes. Their results are nicely summarized in Figure 33, which has been taken from their paper. It visualizes how the binding of the 1–100 segment of α-synuclein binds to a lipid membrane as a function of POPG content in a POPG–DMPC mixture. In the absence of the ionic lipid, the data are not indicative of any binding. The N-terminal 1–30 segment is weakly bound at 25 mol% POPG and strongly at 50 mol%. The 38–60 segment starts to interact at 50 mol% POPG, and the NAC sequence becomes involved at 75%. For the C-terminal segment, it was found that it did not interact with the membrane. At high fractions of POPG (high charge density), two helices were formed that became nearly parallel-oriented on membranes composed of 100% POPG.

When I discussed the binding of cytochrome c to cardiolipin-containing membranes, I was not very much concerned about membrane phases owing to the low melting temperature of such membranes. However, in the case of PG and PC membranes, frequently used for α-synuclein-binding studies owing to their biological relevance, the melting temperature depends very much on the choice of the fatty acid chain. POPC and POPG have a subzero transition temperature, and hence they are in the liquid-crystalline phase at room temperature. Multilamellar vesicles composed of DPPC and DPPG, however, have a very sharp phase transition between 41 and 42 °C. For SUVs, the transition is broader and thus less cooperative. Pirc et al. reported significant changes in the UVCD spectrum of α-synuclein in the presence of an equimolar mixture of POPC and POPG, indicating a coil-to-helix transition [113]. On the contrary, similar measurements with DPPC and DPPG right below the transition temperature did not reveal any significant protein binding. This result suggests that a liquid-crystalline phase promotes α-synuclein binding.

The selected binding studies discussed thus far seem to convey a very clear picture of α-synuclein–lipid interactions. The binding process is predominantly electrostatic in nature and involves helix formation in the amphiphilic 1–100 region, with the hydrophobic site interacting with the interior of the lipid membrane. SUVs favor binding over LUVs and GUVs because of their larger curvature. Binding itself induces (anisotropic) curvature. The binding process is cooperative, which leads to a situation where only a fraction of vesicles is actually occupied by proteins. Effective binding seems to require a crystalline gel phase. Unfortunately, a closer look at a broader scope of literature clouds this picture over. As a matter of fact, to any of the findings listed above, there seems to exist results arguing to the contrary. There is no way to have all this literature covered in this article. To provide a representative example, I focus on an earlier study of Middleton and Rhoades, who used fluorescence correlation spectroscopy to probe the binding of wild-type α-synuclein and of three-point mutants, namely, A30P, E46K, and A53T [114]. The first mutation inserts a helix-breaking residue, the second one increases the positive net charge, and the third one replaces alanine with a bulkier, though more polar residue with hydrogen-bonding capability. In addition to the effective dissociation constant the authors determined, the so-called partition coefficient defined as:

(19) $$K_{p}=\frac{\left[\alpha {syn}_{lipid}\right]}{\left[\alpha {syn}_{free}\right]}$$

**Figure 34:**
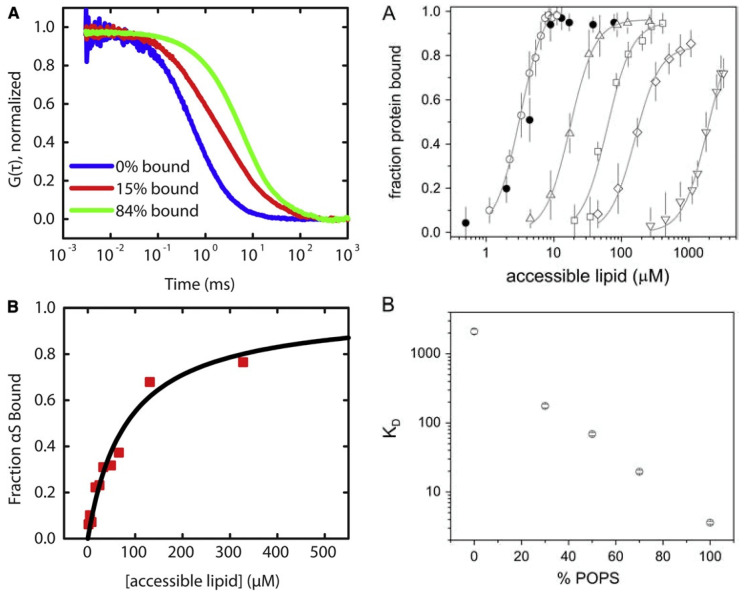
Left: Binding of α-synuclein to 50 mol% POPS–50 mol% POPC vesicles. (**A**) Normalized autocorrelation curves of α-synuclein display a shift to longer times in the presence of vesicles (left to right, increasing lipid concentration). (**B**) α-Synuclein-binding isotherm derived from FCS measurements. The solid line results from a fit of a Langmuir-type function to the depicted data. Taken from ref. [114] with permission. Elsevier, 2010. Right: (**A**) Fraction of α-synuclein bound to POPS- and POPC-containing liposomes plotted as a function of lipid concentration for five different vesicle compositions. The concentration of the protein was maintained at 200 nM, whereas the concentration of lipid was increased: 100% POPS (circles), 70% POPS–30% POPC (triangles), 50% POPS–50% POPC (squares), 30% POPS–70% POPC (diamonds), and 100% POPC (inverted triangles). Also shown is the binding isotherm for 60 nm-diameter 100% POPS vesicles (solid circles). The fits shown in shaded representation are to the Hill equation (Equation (1)) (**B**) The log of *K_D_* values as determined from the fits of the Hill equation to binding curves shown in panel A, plotted as a function of molar percentage POPS. Taken from ref. [115] with permission. Elsevier 2006.

The first surprising result of their data analysis is shown in Figure 34. It depicts the binding isotherm apparently obtained with vesicles with 50 mol% POPS–50 mol% POPC. Unfortunately, it is unclear whether the authors used LUVs or SUVs for obtaining this data set. Obviously, however, the binding curve is hyperbolic, thus suggesting the absence of cooperativity. The authors explain the discrepancy between their results and the ones of other studies (reporting cooperativity) with different unspecified experimental conditions. A closer look at the cited literature reveals that most of these studies focus on α-synuclein aggregation. Only one of the studies cited by Rhoades et al. reports binding isotherms, again based on fluorescence correlation measurements and performed with different mixtures of POPS and POPC. As one can clearly infer from Figure 34, the binding curves are sigmoidal. Since the accessible lipid concentrations are plotted on a logarithmic scale, this does not per se suggest cooperativity. However, the authors report a Hill coefficient of 2 for all these plots, so some cooperativity seems to be present. A close look at the experimental conditions reveal that these experiments were performed at neutral pH, while those of Middelton and Rhoades were carried out at pH 5. Needless to say, this difference alone is unlikely to be the reason for the qualitative discrepancy obtained.

A comparison of dissociation constants obtained for different POPS–POPC mixtures suggests a linear increase in effective binding affinity with increasing membrane charge. This observation is in line with expectations for electrostatic binding. The addition of salt reduces the fraction of bound proteins. While Rhoades et al. interpret this as indicative of binding inhibition [115], the presence of a still-substantial fraction of bound proteins at 100 mM NaCl (50% of what was observed in the absence of salt) does not seem to reflect electrostatic screening (cf. the work of Davidson et al. [103], who reported a similar result). The above studies of cytochrome *c* binding to cardiolipin have clearly demonstrated that an assessment of the influence of salt should be conducted based on a thorough analysis of binding isotherms, rather than on the behavior of a single data point.

**Figure 35:**
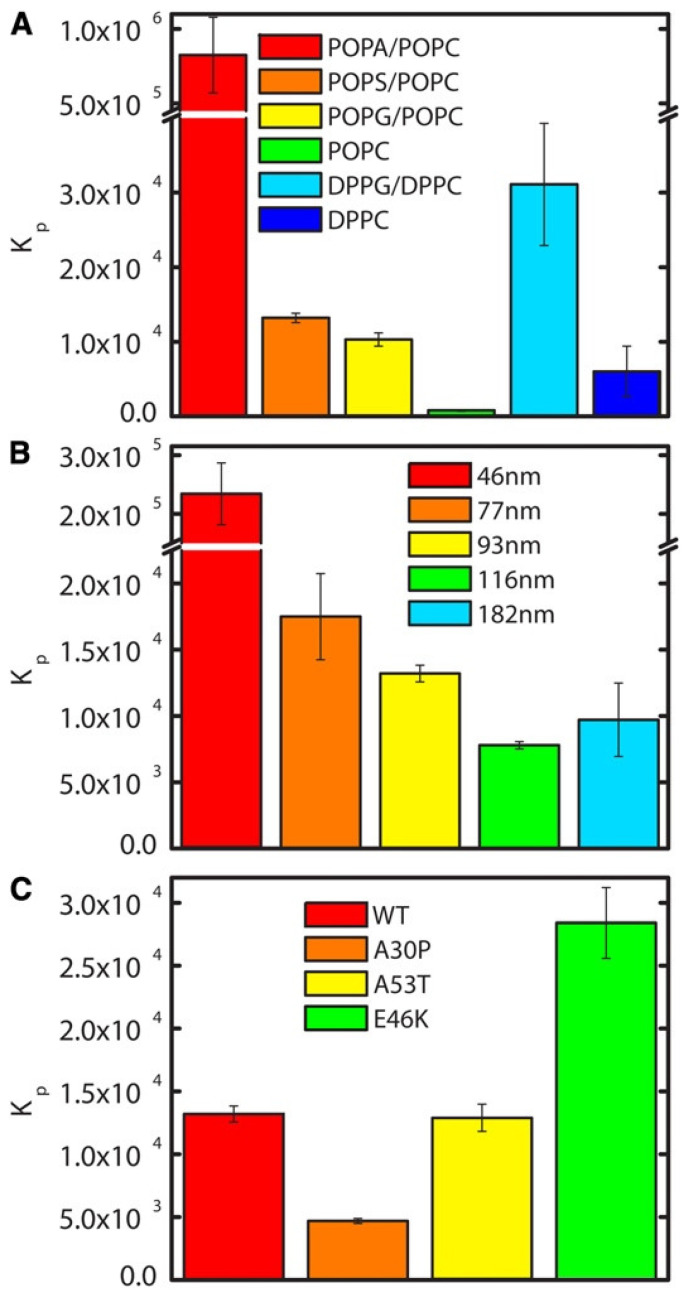
Molar partition coefficients (*K_p_*) obtained for α-synuclein binding to liposomes containing the indicated lipid compositions. Comparison of *K_p_* values measured for (**A**) α-synuclein with 93 nm LUVs of six different lipid compositions, (**B**) α-synuclein with 1:1 POPS–POPC vesicles covering a range of diameters, and (**C**) wild-type α-synuclein and Parkinson’s disease-associated mutants. Taken from ref. [114].

The binding affinities reported by Middeton and Rhoades range between 6.25 × 10^5^ M^−1^ for a 50:50 mol % mixture of POPA and POPC and 5.92 × 10^2^ M^−1^ for 100% POPC. Mixtures of POPC with POPS and POPG lie somewhere in between these extremes (note that I am preferring to use association rather than dissociation constants to document binding affinity). In view of the different effective charges of the corresponding head groups, such a result is somewhat expected. However, it is interesting to note that the *K_p_* values visualized in Figure 35A tell a slightly different story. POPA–POPC still dominates, but the value for 100% POPC is still significant. Thus, POPC can still bind a significant number of proteins, just with lower affinity. Figure 35B shows how *K_p_* depends on the size of the vesicles (for POPA–POPC). The depicted numbers seem to be in line with the notion that more curvature (SUVs versus LUVs) means more binding. As one would expect, the replacement of A30 by P decreases the binding because it affects helical formation, while the increase in the net charge (E46K) increases the partition coefficient. Surprisingly, the same was observed for the gel phase of the membrane for which a higher *K_p_* value was obtained than for the corresponding liquid phase. If one relates this finding to the results of Pirc et al. [113], one wonders about the exact relationship between the binding affinity *1/K_D_* and the partition value *K_p_.*

I finish this section by very briefly outlining the results of a linear dichroism study by Rocha et al. [116]. Linear dichroism can be used to probe the orientation of molecules if the direction of their electronic transition dipole moment is known. Rather than using lipid mixtures, the authors used homogeneous membranes with 100% DOPS, POPS, and DOPG. They utilized the direction of the 208 nm π→π* transition of the N-terminal helix of membrane-bound α-synuclein. The analysis of the linear dichroism spectra of proteins on flow-aligned vesicles revealed the following picture. At low lipid-to-protein ratios (<100), the helices are uniformly distributed on the membrane surface. In other words, they are isotopically oriented on the surface. At high lipid-to-protein ratios, where the protein density decreases, the proteins are preferentially oriented perpendicularly to the surface normal and along the direction of the least curvature with respect to the surface. While the different behavior in the two regimes can be easily understood by the presence and absence of random molecular crowding effects, the orientation along the direction of minimal curvature comes as a surprise, owing to the many results mentioned above that suggest the binding-supporting function of membrane curvature [103,105,114,115].

As for cytochrome *c*, the current picture of monomeric α-synuclein binding to anionic lipid membranes is still somewhat muddy. It is clear that binding involves a structural change involving the residues 1–100. Depending on conditions, the formed secondary structure might be a single helix or several helical segments. This segment of the protein is amphiphilic: one site is hydrophilic with a positive net charge, while the other one is hydrophobic. Binding models suggest that while the electrostatic contribution to the binding free energy must be significant, the hydrophobic interactions involving membrane lipid chains cannot be disregarded. To the best of my knowledge, the two steps of binding, i.e., the binding of the disordered protein and the subsequent disorder-to-order transitions, have not yet been disentangled. The exact partition between the binding energy contributions of electrostatic and hydrophobic binding is outstanding. The influence of the salt (NaCl) on α-synuclein binding is obvious, but not well understood from a theoretical point of view. Attempts to rationalize in terms of sodium ion binding to the membrane surface and electrostatic screening, as done for cytochrome *c*–anionic lipid interactions, have not been undertaken. This might be a difficult task, since the disordered protein, which actually has a negative net charge at physiological pH, cannot be approximated by a sphere, as has been done for the folded oxidized cytochrome *c* [27]. The influence of vesicle curvature on α-synuclein binding is obvious, but one has to take into account that even flat bilayers are susceptible to binding. The role of and the underlying forces governing cooperativity remain uncertain. Here, more systematic investigation of the pH and possibly of the salt dependence of binding isotherms needs to be carried out. Finally, contradictions between the influence of the bilayer phase on the apparent binding isotherm need to be resolved.

### 4.3. α-Synuclein Aggregation and Self-Assembly

There are several reasons for researchers to study α-synuclein. First, it is a prominent representative of the family of intrinsically disordered proteins that adopt a statistical coil structure in solution [98,117]. Second, investigations are targeted to understand its positive biological role. Third, and these studies dominate the field, researchers are interested in delineating the role that α-synuclein self-assembly plays in the development of Parkinson’s disease [102].

**Figure 36:**
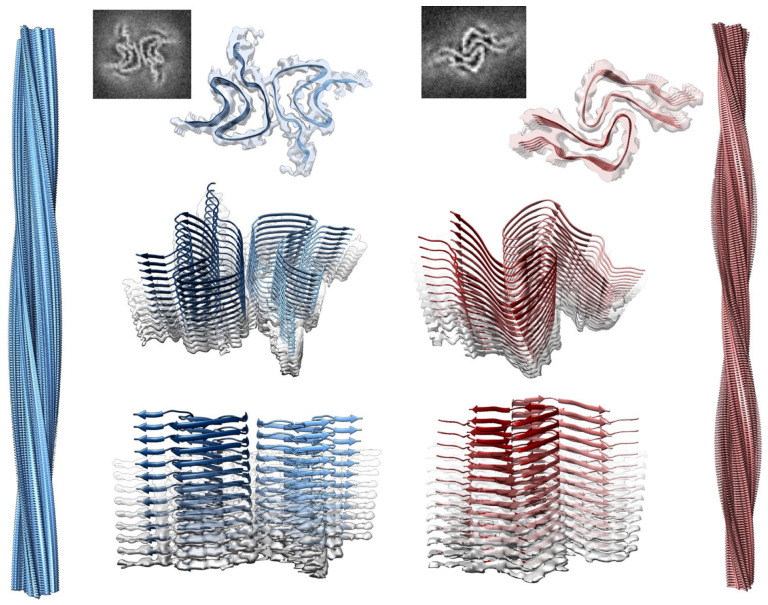
Cryo-EM structures of α-synuclein rod and twister polymorphs. The structures of the rod (left) and twister (right) polymorphs of the full-length protein fibrils, shown as density slices (top inlet), and as semitransparent surfaces overlaid with their atomic models viewed from two different angles (lower panels). The rod (blue) and twister (red) polymorphs contain two protofilaments composed of stacked β-sheets and packed by an approximate 2_1_ screw axis of symmetry. Shown on the left and right are 3D models of the rod and twister fibril polymorphs, respectively. Taken from ref. [118] (open access).

A lot of studies targeting the latter issue have been carried out in solution. This notion applies in particular to structural studies. However, this article focuses on the protein interactions with membranes. Therefore, I predominantly present and discuss some selected studies of the conditions that facilitate the formation of α-synuclein oligomers and fibrils on the surface of anionic lipid membranes. However, for orientational purposes, I start with the results of a structural study of α-synuclein in solution by Li et al. conducted with cryo-electron microscopy [118]. I focus on the results; technical details can be inferred from their paper. The authors identified two polymorphs, termed rod and twister, that are shown in Figure 36 from various perspectives. In both cases, the fibrils are helically twisted. show The bent β-strand arches of the two polymorphs. The corresponding cores of tightly packed zippers resemble to some extent the structure of crystalline fibrils formed by the GNNQQNY fragment of the yeast prion protein SUP35p [119]. The obtained structure itself shows a lot of elements observed for other amyloid peptide and protein fibrils. An overview can be found in a recent monograph [120].

**Figure 37:**
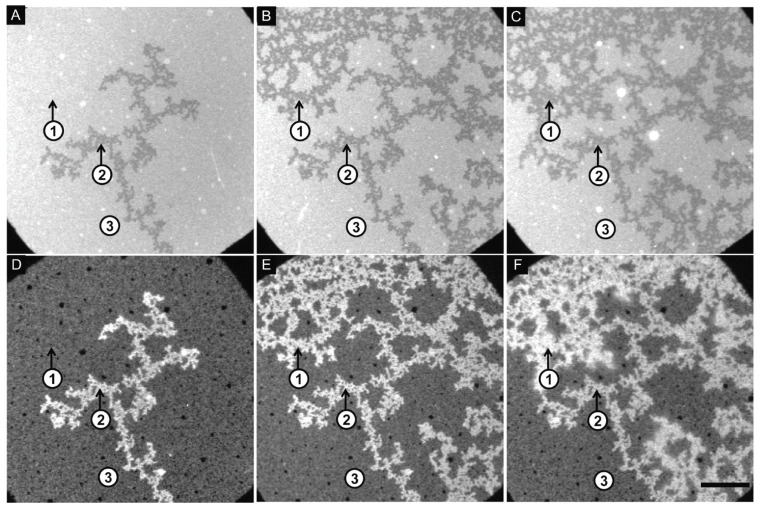
Microscopic images depicting the sequential addition of α-synuclein to a PA–PC bilayer at pH 5.0. Epifluorescence images of a 30 mol % DOPA–69 mol % DOPC–1 mol % NBD–PC bilayer at pH 5.0, 100 mM NaCl. Lipid bilayers (**A**–**C**) and corresponding protein images (**D**–**F**) are shown after successive additions of protein at 0.26 µm (**A**,**D**), 1.3 µm (**B**,**E**), and 1.3 µm (**C**,**F**) of α-synuclein. Three regions are highlighted by numbers and arrows: region 1 (initially PC-rich, then PA-rich); region 2 (PA-rich); and region 3 (PC-rich). The scale bar represents 40 µm. Taken from ref. [121] with permission. American Chemical Society 2010.

I start with a paper by Haque et al., who used fluorescence imaging to probe the interaction between α-synuclein and vesicles composed of 30 mol% DOPA–69 mol% DOPC–1 mol% NBD-PC [121]. The latter is a lipid fluorophore. Figure 37 shows images of lipid bilayers to which α-synuclein in different concentrations had been added at pH 5 and 100 mM NaCl. The dark areas in A–C indicate the clustering of DOPA due to lipid–lipid demixing. D–F depict the corresponding binding of α-synuclein. Interestingly, the clustering of DOPA produces fractal structures. The images clearly show that only DOPA is able to bind the protein. The authors carried out a similar investigation at pH 7.4, where the lipid–lipid phase separation led to a stronger demixing. Since the head group DOPA has two protonable sites with rather different pK values (3.2 and 7.9), the charge density of the membrane is higher, at pH 7.4. The authors did not obtain any structural data. Just based on their images and the results of a switch experiment (from pH 5 to 7.4), they proposed a model that hypothesized the formation of two helical segments upon protein binding at pH 5, while they suggested a single long helix at 7.4. While the authors talked a lot about protein aggregation on the utilized membrane surface, it is not clear at all whether it actually happens. Images like the one in Figure 37 in fact only prove α-synuclein binding directly while providing indirect evidence of protein self-assembly.

**Figure 38:**
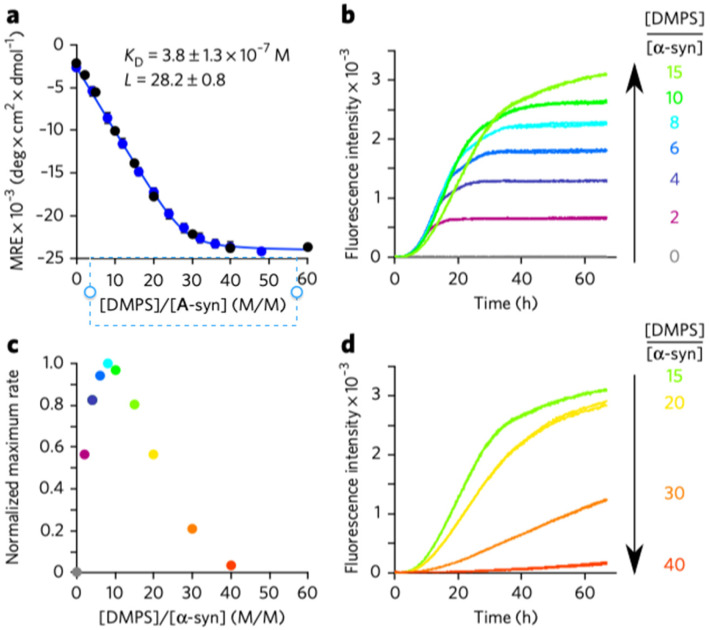
(**a**) Change in the UVCD signal of α-synuclein (blue, 25 MM; black, 50 μM) measured at 222 nm as a function of DMPS/α-synuclein (M/M) ratios. The data (blue dots, 25 μM) fit well to a single-step Langmuir-type binding model (blue line; *K_D_*= 3.8. 1.3 × 10^−7^ M). (**b**,**d**) Duplicates of fluorescence measurements monitored as a function of time when α-synuclein (50 mM) was incubated in the absence (gray) and presence of increasing concentrations of DMPS: 100 μM (purple), 200 μM (dark blue), 300 μM (blue), 400 μM (cyan), 500 μM (green), 750 μM (light green), 1000 μM (yellow), 1500 μM (orange), and 2000 μM (red). (**c**) Variation in the maximum rate of aggregation of α-synuclein with changes in the DMPS/α-synuclein ratio. Taken from ref. [122] with permission. Springer 2015.

Galvagnian et al. offered a very thorough investigation of α-synuclein binding to anionic lipids and requirements for subsequent oligomerization and fibril formation [122]. The authors used SUVs exclusively formed with 1,2 dimystriol-*sn*-glycerol-3-phosphoserine (DMPS). The binding of the protein to these vesicles was monitored by the change in UVCD. Protein self-assembly in the presence of DMPs was probed by thioflavine fluorescence. Measurements were made at 30 °C. The main results can all be found in Figure 38. The binding isotherm (change in ellipticity as a function of the lipid-to-protein ratio) suggests non-cooperative binding with a *K_D_*-value of 3.8 × 10^−7^ M (equilibrium constant of 2.6 × 10^6^ M^−1^) and a surprisingly large stoichiometry of 28.2. The authors did not find any indication that the size of their vesicles mattered for the results between 20 to 100 nm. They did not observe any thioflavin fluorescence for lipid/protein ratios above 40. When the authors added an excess of DMPS SUVs to preformed fibrils, the latter dissociated into monomers. Contrary to these scenarios, rapid fibrilization was observed for lipid-to-protein ratios between 1 and 15, accompanied by a depletion in monomeric proteins in the bulk. The maximal rate was found at a lipid/protein fraction of 8 (Figure 38c).

**Figure 39:**
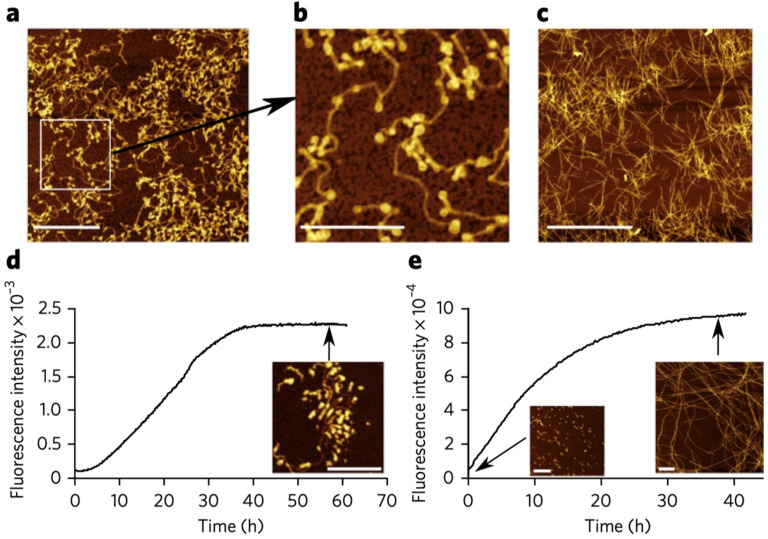
(**a**,**b**) Atomic force microscopy images of α-synuclein aggregates formed after incubation of 200 μM monomeric protein in the presence of 600 μM DMPS SUVs. (**b**) Expanded region of the image in (**a**). (**c**) AFM image of aggregates of α-synuclein formed in the presence of preformed seed fibrils. (**d**,**e**) Changes in the thioflavin fluorescence signal observed when the solution of the remaining free monomers was incubated in the presence of fresh DMPS SUVs (300 μM) and (**d**) after sonication of the reaction mixture for 10 s at the end of the process of amyloid formation. (**e**) The concentrations of α-synuclein converted into fibrils were found to be 20 μM and 50 μM in (**d**,**e**), respectively. The scale bars of the AFM images correspond to 1 μm in (**a**,**e**), 500 nm in (**b**,**d**), and 4 μm in (**c**). Taken from ref. [122] with permission. Springer 2015.

**Figure 40:**
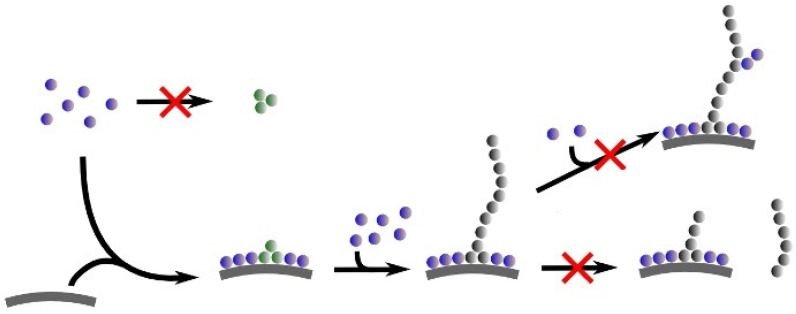
Reaction scheme proposed for the fibril formation of α-synuclein in the presence of DMPS SUVs. The red x signs indicate forbidden pathways. Taken from ref. [122] with permission. Springer 2015.

AFM images taken in the plateau region of the kinetic traces are shown in Figure 39. They reveal SUVs coated with α-synuclein and filaments attached to the vesicle surface. The authors found no hint of the occurrence of fragmentation or thus secondary nucleation under their experimental conditions. They employed a kinetic model, visualized in Figure 40, to fit to their data. Details can be inferred from the Supporting Information of their paper. In their model, only chain extension and branching occur.

The ionic strength dependence of α-synuclein self-assembly in the presence of SUVs was examined by Gaspar et al. in conjunction with investigation of this protein’s aggregation in solution [123]. They used 90 mol% DOPC–10 mol% DOPS at pH 5.5. Thus, these vesicles carried a very low charge density. They did not observe any thioflavin fluorescence in the absence of vesicles over a period of 100 h. The presence of SUVs led to protein self-assembly after ca. 50 h for lipid/protein ratios up to 20. No self-assembly was observed in the presence of 140 mM NaCl. A less pronounced effect was observed for self-assembly processes in solution that were facilitated by seeds. The authors discussed the observation solely in terms of protein–protein interactions, for which they invoked a decrease in attracting electrostatic forces over the screening of repulsive interactions. However, an understanding of their observation would require an assessment of the influence of NaCl on the density of bound proteins. Owing to the low charge density of the employed vesicles, 140 mM NaCl might be sufficient to reduce the concentration of bound proteins below the threshold for self-assembly.

Kiskis et al. investigated DOPG and DOPS interactions with α-synuclein with UVCD spectroscopy and atomic force microscopy [124]. Protein self-assembly was investigated with thioflavin fluorescence. The binding isotherms inferred from the changes in the CD spectrum did not indicate any cooperativity. Surprisingly, they suggest that the binding affinity for DOPG is lower by a factor of four than the one obtained with DOPS. Since the final spectra are indicative of helical conformations, self-assembly did not start during the binding experiment. For DOPG, thioflavin kinetics suggested a maximum of self-assembly when 20% of α-synuclein was found to bind to the vesicle surface, but it was still significant with 10% and 55% of bound protein. Surprisingly, only 10% binding to DOPS produced protein self-assembly. The lag time was found to be longer by a factor of 2 for DOPS. The authors explained their results qualitatively by stronger electrostatic interactions between the head groups of PS, which they expected to lead to a higher order (compared with PG) and thus to a higher exposure of hydrophobic parts of DOPG lipids.

## 5. Cytochrome *c* and α-Synuclein

**Figure 41:**
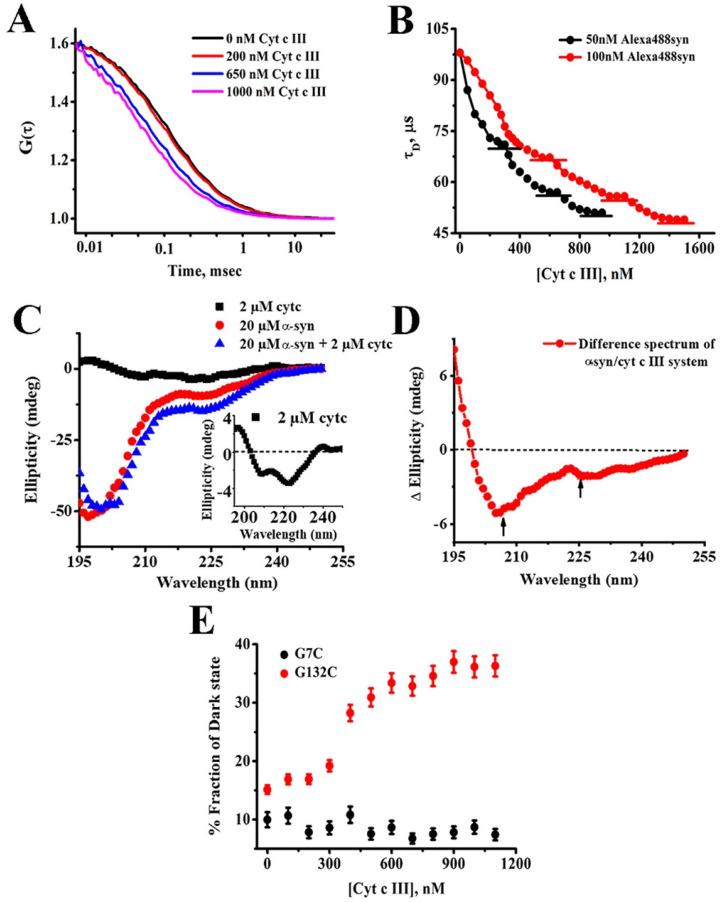
(**A**) Variation in correlation functions of fluorescence-labeled α-synuclein obtained in the absence and presence of varying concentrations of cytochrome *c*. (**B**) Variation in diffusion time of Alexa488-α-synuclein with varying concentrations of cytochrome *c*. (**C**) UVCD profile of natively unfolded α-synuclein in the absence and presence of ferricytochrome *c*; inset shows CD profile of the heme protein. (**D**) Difference spectrum of α-synuclein–ferricytochrome *c*. (**E**) Contribution of dark state of Alexa488syn (G7C and G132C) on addition of cytochrome *c*, indicating a C-terminal-specific binding. Taken from ref. [125] with permission. American Chemical Society 2019.

I would like to round up this article by briefly mentioning the interplay between cytochrome *c* and α-synuclein. The involvement of both proteins in the development of Parkinson’s disease has been suggested by their co-localization in Lewy bodies of Parkinson’s patients. Kumar et al. showed that peroxidase activity of cytochrome *c* contributes to the formation of α-synuclein radicals and its subsequent assembly into oligomers [11,125]. They concluded that α-synuclein, by virtue of its co-localization with cytochrome *c*, affects biological pathways that contribute to increased neuronal death in Parkinson’s disease caused by pesticides. The role of cytochrome *c*-induced oxidative stress was also demonstrated by Hashimoto et al. [10]. Ghosh et al. went into more detail by exploring the interactions between both proteins by spectroscopic means [125]. In doing so, they focused on the possibility of redox reactions that could involve the tyrosine residues in both proteins. They used fluorescence-labeled α-synuclein (Alexa-488 tagged to G132C) in fluorescence correlation and CD spectroscopy experiments to probe the interaction between cytochrome *c* and α-synuclein. For oxidized cytochrome *c*, the results are summarized in Figure 41. Figure 41A shows the change in the correlation function of α-synuclein as a function of cytochrome *c* concentration. The dependence of the diffusion time constant on the lipid concentration is shown in Figure 41B for two α-synuclein concentrations. The data reflect a decrease in the radius of hydration due to cytochrome *c* binding. The plot in Figure 41B exhibits a rather low slope that could be indicative of intermediates. Figure 41C compares the UVCD spectra of α-synuclein in the absence and presence of cytochrome *c*. Both spectra reflect the predominance of a statistical coil conformation. However, the difference spectrum in Figure 41D seems to suggest the existence of a helical fraction. The authors reported that the helical fraction increased with cytochrome *c* concentration. However, it is somewhat unclear to what extent this solely reflects the presence of the folded heme protein. Figure 41E is interesting, because it shows the population of the dark state for G7C- and G132 C-labeled α-synuclein. The dark state is produced by fluorescence energy transfer from the Alexa-488 fluorophore to the heme group of cytochrome *c*. The different response of the two labels clearly shows that the C- rather than the N-terminal segment of α-synuclein is involved in its interaction with oxidized cytochrome *c*. This is not surprising, owing to the abovementioned large positive net charge of the latter. Binding was also indicated by a change in fluorescence anisotropy. Experiments with reduced cytochrome *c* led to rather different observations. The radius of hydration was found to increase with increasing cytochrome c concentrations, while the fluorescence anisotropy remained unaffected.

**Figure 42:**
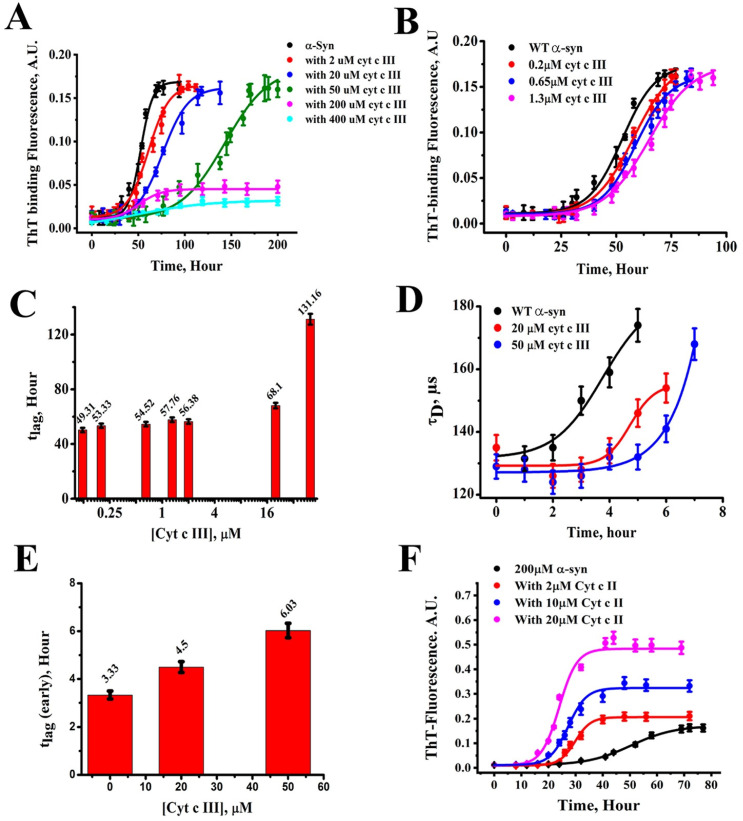
(**A**) Late-stage α-synuclein aggregation kinetics in the presence of the indicated concentrations of ferricytochrome *c*. (**B**) Late-stage aggregation of α-synuclein in the presence of the indicated concentrations of ferricytochrome *c* corresponding to midpoints of the transitions indicated subtle changes in lag time. (**C**) Plot of lag time (*t_lag_*) versus concentration of ferricytochrome *c*. (**D**) Early-stage aggregation kinetics of α-synuclein with 20 and 50 μM of ferricytochrome *c*. (**E**) *t_lag_* value for early aggregation of α-synuclein without and with ferricytochrome *c* (20 and 50 μM). (**F**) 200 μM α-synuclein aggregation kinetics probed by thioflavine fluorescence without (black) and with 2, 10, and 20 μM ferrocytochrome *c*. Taken from ref. [125] with permission. American Chemical Society 2019.

The self-assembly of α-synuclein in the absence and presence of oxidized and reduced cytochrome *c* was probed by thioflavin fluorescence (Figure 42). The data indicate that the oxidized heme protein delays fibrilization at low and medium concentrations and reduces the number of fibrils at very high concentrations. The effect of reduced cytochrome *c* goes in the opposite direction, i.e., self-assembly becomes accelerated and enhanced. Qualitatively, these results were corroborated by atomic force microscopy. Images of α-synuclein in the presence of 20 μM cytochrome *c* revealed only oligomers after 40 h incubation. For the protofibrils or fibrils to show up, the researchers waited for 96 h. In the absence of the heme protein, α-synuclein formed fibrils after 40 h. On the contrary, reduced cytochrome *c* produced oligomers after 24 h and protofibrils after 72 h.

The results of the above experiments and further experiments with free radical quenchers and the measurement of dityrosine fluorescence suggest that self-assembly is influenced by some type of redox process involving the formation of tyrosinate ions. The authors suggested that the binding of cytochrome *c* to α-synuclein facilitates heme reduction, the formation of tyrosine radicals, and subsequently the formation of dityrosine adducts and hetero-oligomers that prevent aggregation. For reduced cytochrome *c*, the authors hypothesized that the increased hydration of K39, K53, and K55 prevents its binding to α-synuclein, which prevents cytochrome *c*–α-synuclein binding and thus promotes dityrosine formation between cytochromes and α-synucleins, which promotes fibril formation down the road. While the reported data are clearly indicative of dityrosine formation, the electrochemistry of the proposed scheme remains somewhat mysterious in that the oxidant source of tyrosinate ions remains unclear. Dityrosine formation requires the presence of radicals. Their formation seems to be plausible in the reaction with oxidized cytochrome *c*, but not with the reduced form of this protein. However, in spite of these unresolved issues, the work of Ghosh et al. clearly documents the interactions between cytochrome *c* and α-synuclein and their influence on the self-assembly propensity of the latter.

Taken together, the above work and further studies provide compelling evidence of the notion that cytochrome *c* can become involved in α-synuclein aggregation and that this process involves the functioning of the heme protein as peroxidase. The molecular details of the process remain to be determined. In this context, it must be determined which sites of α-synuclein bind (at least temporarily) to cytochrome *c* and how the underlying protein–protein interactions affect the structure at the active site of the heme protein. If α-synuclein operates as a substrate of cytochrome *c* in the mitochondria, it can function as an inhibitor of the normal apoptosis cascade, which is initiated by the interaction between cytochrome *c* and apaf1. Generally, the extent to which cytochrome *c* contributes to Parkinson’s disease-relevant self-assembly of α-synuclein still needs to be determined. Aggregation of the latter via dityrosine formation would add a covalent component to the formed fibrils, which should make them much more stable compared with supramolecular structures solely formed by non-covalent interactions.

## 6. Conclusions

This Perspectives article focuses on delineating the complexity of the interactions between peripheral binding proteins and biological membranes. In order to avoid just a lexicographic presentation of all types of these proteins, I focused on two representative and to some extent complementary examples, namely, cytochrome *c* and α-synuclein. These two proteins represent two types of membrane-binding proteins, which both predominantly bind to anionic lipids. Cardiolipin is a preferred target of cytochrome *c* binding to the inner membrane of mitochondria. While this protein is in a folded state in solution, a fraction of the bound protein undergoes a conformational transition into a partially unfolded state, in which the axial ligand M80 is replaced by a histidine residue (at neutral pH) [73,126]. At acidic pH, upon the protonation of the imidazole side chain, the protein switches to a penta- or hexacoordinate high-spin state [54]. Generally, the conformational transition is inhibited if the protein density on the membrane surface is high. It is likely that the peroxidase activity of cytochrome *c* is associated with the partially unfolded state, but experimental results do not provide a clear picture in this regard. The folded membrane-bound state is likely to be the one that functions as an electron transfer protein. Reduced cytochrome is destabilized at high lipid/protein ratios where a fraction of the protein is in the partially unfolded state. The lipid-to-protein density also plays a role in regulating the binding of α-synuclein to anionic lipid membranes. It regulates the concomitant coil → helix transitions and the propensity of the protein for self-assembly on the membrane surface (high at low and low at low lipid-to-protein ratios). For both proteins, electrostatic binding seems to play a decisive role. While the entire protein is involved in cytochrome *c* binding, even with different binding sites at different pH, α-synuclein binding solely involves the first hundred amino acid residues. Electrostatic binding has generally been deduced from experiments in which salt (NaCl) was added to investigated protein–vesicle samples. However, salt can affect protein binding by binding to oppositely charged groups and by screening the electric charges on both the membrane surface and the protein. The strength of any salt bridges formed during the binding process depends on the exact position of the charged protein residue with respect to the lipid head groups. For neither of the two proteins considered in this article was structural site-specific mapping undertaken (certainly a difficult task).

Very limited efforts have been made to disentangle the contributions of both to the binding of the two proteins. It is obvious from available experimental data that hydrophobic interaction contributes to the binding of both proteins, but efforts to quantitatively determine its contribution to overall binding affinity are rather limited. For cytochrome *c* binding, the lipid anchorage model has been invoked as a mechanism for hydrophobic binding, but the jury is still out regarding its validity. With regard to α-synuclein binding to anionic vesicles, binding isotherms are conflicting, in that some suggest cooperativity while others do not. While a significant number of experiments with cytochrome *c* shed some light on the structural conversion of the protein on membrane surfaces, similar efforts regarding α-synuclein binding are in very short supply. Another unresolved issue is membrane penetration. For both proteins, experimental evidence suggests the formation of membrane pores, but it is unclear to which extent these findings are consistent with the results of binding studies. Altogether, more work has been invested in the physical chemistry of cytochrome *c*–lipid interactions. Generally, more systematic investigations of the binding and in the case of α-synuclein of the self-assembly process on lipid and solution parameters are still desirable. The same can be said about physical modeling. Physicochemical studies on α-synuclein are all relatively recent, while work on cytochrome *c*–lipid interactions has a long history. It is obvious that the latter provides some insights for the understanding of the latter, in spite of the opposite structural processes involved. At the end of this article, I briefly described literature that revealed that both proteins are likely to be involved in Parkinson’s disease. These findings underscore the notion that cytochrome *c* is a multifunctional protein and by no means solely the subject of past research.

Table 4 summarizes and compares the main aspects of the binding of cytochrome *c* and α-synuclein to anionic lipid membranes. Apparently, there are actually a lot of similarities in spite of the respective structural changes moving in opposite directions.

## Figures and Tables

**Table 1:** Thermodynamic parameters obtained from fits of Hill functions to binding isotherms of cytochrome *c* binding to 100 mol% TOCL. Taken from ref. [57].

Variant	*K_d,app_* ([Cl]/[cyt]) Soret Band CD	*n*	*K_d,app_* ([Cl]/[cyt]) Fluorescence	*n*
Yeast wild type	10.2	2.2	23.4	2.3
Yeast K72A	9.8	2.0	26.2	2.3
Human wild type	8.6	1.3	36.0	2.2
Huan K72A	8.4	1.1	40.5	1.9

**Table 2:** Apparent dissociation constants inferred from CD- and W59-based fluorescence binding isotherms probing the interaction of oxidized yeast iso-1 cytochrome *c* to the inner and outer leaflet of 100% TOCL vesicles. Taken from ref. [63].

Membrane Curvature	*K_d_* [Exposed Lipid/Protein] Soret Band CD	*K_d_* [Exposed Lipid/Protein] Fluorescence
concave	55	61
convex	10.2	23.4

**Table 3:** Comparison of α-synuclein binding to unilamellar phospholipid liposomes of different sizes. Taken from ref. [103].

Vesicle	Binding to SUV [μg Protein/mg Lipid]	Binding to LUV [μg Protein/mg Lipid])
1:1 (*w*/*w*) PC/PA	120	40
PC only	2	>1

**Table 4:** Comparison of physicochemical aspects governing the binding of cytochrome c and α-synuclein binding to anionic lipid membranes.

Mechanisms and Processes	Cytochrome *c*	α-Synuclein
**Structural changes**	Partial population of a partially unfolded state, increasing towards higher lipid/protein ratios.	Disorder to order (helical) transitions of the N-terminal segment of the protein (residues 1–100). Specifics depend on the lipid-to-protein ratio.
**Binding sites**	Four different binding sites have been identified. The relative contributions depend on solution conditions (pH an ionic strength).	Only the N-terminal segment is involved in the binding to anionic surfaces.
**Changes to the membrane**	Conflicting results ranging from slight membrane penetration to induced membrane curvature and even pore formation.	A high degree of curvature facilitates protein binding to anionic surfaces.
**Electrostatic contributions**	Electrostatic interactions are involved in cytochrome *c* binding via its A- and L-sites, but their contribution depends on the pH of the solution.	Electrostatic interactions (with anionic lipids) significantly increase the binding affinity.
**Hydrophobic contributions**	Hydrophobic interactions and H-bonding has been proposed for C-site binding and the corresponding involvement of lipid insertion. It is unclear to what extent it happens solely for C-site binding, which was suggested to occur at acidic pH.	While electrostatic interactions dominate, N-terminal binding also encompasses hydrophobic binding owing to its amphiphilic character. The addition of NaCl causes only a partial inhibition of protein binding.
**Protein aggregation**	Involvement of protein aggregation is unclear. It might occur at low lipid-to-protein ratios. Whether or not domain-swapped dimers are formed remains to be determined.	Protein aggregation and fibril formation is facilitated at low lipid/protein ratios. In the presence of cytochrome c and H_2_O_2_, dityrosine formation might add a covalent component.
**Functional changes**	Cytochrome becomes a peroxidase. The balancing between electron transfer and peroxidase activity is still not fully understood.	The normal function of the protein is not entirely clear, nor is its modification by membrane binding.

## Data Availability

No new data were created or analyzed in this study.

## References

[1] Lewis R.N.A.H., McElhaney R.N. (2009). The Physicochemical Properties of Cardiolipin Bilayers and Cardiolipin-Containing Lipid Membranes. Biochim. Biophys. Acta Biomembr..

[2] Ryan T., Bamm V.V., Stykel M.G., Coackley C.L., Humphries K.M., Jamieson-Williams R., Ambasudhan R., Mosser D.D., Lipton S.A., Harauz G. (2018). Cardiolipin Exposure on the Outer Mitochondrial Membrane Modulates α-Synuclein. Nat. Commun..

[3] Monje-Galvan V., Klauda J.B. (2016). Peripheral Membrane Proteins: Tying the Knot between Experiment and Computation. Biochim. Biophys. Acta (BBA)—Biomembr..

[4] Hannibal L., Tomasina F., Capdevila D.A., Demicheli V., Tórtora V., Alvarez-Paggi D., Jemmerson R., Murgida D.H., Radi R. (2016). Alternative Conformations of Cytochrome *c*: Structure, Function, and Detection. Biochemistry.

[5] Alvarez-Paggi D., Hannibal L., Castro M.A., Oviedo-Rouco S., Demicheli V., Tórtora V., Tomasina F., Radi R., Murgida D.H. (2017). Multifunctional Cytochrome *c*: Learning New Tricks from an Old Dog. Chem. Rev..

[6] Schweitzer-Stenner R. (2018). Relating the Multi-Functionality of Cytochrome *c* to Membrane Binding and Structural Conversion. Biophys. Rev..

[7] Vincent J.S., Kon H., Levin I.W. (1987). Low-Temperature Electron Paramagnetic Resonance Study of the Ferricytochrome *c*-Cardiolipin Complex. Biochemistry.

[8] Das T., Eliezer D. (2019). Membrane Interactions of Intrinsically Disordered Proteins: The Example of Alpha-Synuclein. Biochim. Biophys. Acta (BBA)—Proteins Proteom..

[9] Fauvet B., Mbefo M.K., Fares M.B., Desobry C., Michael S., Ardah M.T., Tsika E., Coune P., Prudent M., Lion N. (2012). α-Synuclein in Central Nervous System and from Erythrocytes, Mammalian Cells, and Escherichia Coli Exists Predominantly as Disordered Monomer. J. Biol. Chem..

[10] Hashimoto M., Takeda A., Hsu L.J., Takenouchi T., Masliah E. (1999). Role of Cytochrome *c* as a Stimulator of α-Synuclein Aggregation in Lewy Body Disease. J. Biol. Chem..

[11] Kumar A., Ganini D., Mason R.P. (2016). Role of Cytochrome *c* in α-Synuclein Radical Formation: Implications of α-Synuclein in Neuronal Death in Maneb- and Paraquat-Induced Model of Parkinson’s Disease. Mol. Neurodegener..

[12] Battistuzzi G., Borsari M., Cowan J.A., Ranieri A., Sola M. (2002). Control of Cytochrome *c* Redox Potential: Axial Ligation and Protein Environment Effects. J. Am. Chem. Soc..

[13] Stryer L. (2002). Biochemistry.

[14] Purring-Koch C., McLendon G. (2000). Cytochrome *c* Binding to Apaf-1: The Effects of DATP and Ionic Strength. Proc. Natl. Acad. Sci. USA.

[15] Atlante A., Calissano P., Bobba A., Azzariti A., Marra E., Passarella S. (2000). Cytochrome *c* Is Released from Mitochondria in a Reactive Oxygen Species (ROS)-Dependent Fashion and Can Operate as a ROS Scavenger and as a Respiratory Substrate in Cerebellar Neurons Undergoing Excitotoxic Death. J. Biol. Chem..

[16] Paradies G., Petrosillo G., Pistolese M., Ruggiero F.M. (2000). The Effect of Reactive Oxygen Species Generated from the Mitochondrial Electron Transport Chain on the Cytochrome *c* Oxidase Activity and on the Cardiolipin Content in Bovine Heart Submitochondrial Particles. FEBS Lett..

[17] Banci L., Bertini I., Reddig T., Turano P. (1998). Monitoring the Conformational Flexibility of Cytochrome *c* at Low Ionic Strength by 1H-NMR Spectroscopy. Eur. J. Biochem..

[18] Soffer J.B. (2013). The Folded, Partially Folded, Misfolded, and Unfolded Conformations of Cytochrome *c* Probed by Optical Spectroscopy. Ph.D. Thesis.

[19] Uversky V.N. (2016). Dancing Protein Clouds: The Strange Biology and Chaotic Physics of Intrinsically Disordered Proteins. J. Biol. Chem..

[20] Uversky V.N. (2002). Natively Unfolded Proteins: A Point Where Biology Waits for Physics. Protein Sci..

[21] Uversky V.N., Li J., Fink A.L. (2001). Evidence for a Partially Folded Intermediate in α-Synuclein Fibril Formation. J. Biol. Chem..

[22] Makasewicz K., Linse S., Sparr E. (2024). Interplay of α-Synuclein with Lipid Membranes: Cooperative Adsorption, Membrane Remodeling and Coaggregation. JACS Au.

[23] Sharma M., Burré J. (2023). α-Synuclein in Synaptic Function and Dysfunction. Trends Neurosci..

[24] Burré J., Sharma M., Tsetsenis T., Buchman V., Etherton M.R., Südhof T.C. (2010). α-Synuclein Promotes SNARE-Complex Assembly in Vivo and in Vitro. Science (1979).

[25] Vicario M., Cieri D., Brini M., Calì T. (2018). The Close Encounter Between Alpha-Synuclein and Mitochondria. Front. Neurosci..

[26] Dill K.A., Bromberg S. (2002). Molecular Driving Forces. Statistical Thermodynamics in Chemistry and Biology.

[27] Heimburg T., Marsh D. (1995). Protein Surface-Distribution and Protein-Protein Interactions in the Binding of Peripheral Proteins to Charged Lipid Membranes. Biophys. J..

[28] Zuckermann M.J., Heimburg T. (2001). Insertion and Pore Formation Driven by Adsorption of Proteins onto Lipid Bilayer Membrane-Water Interfaces. Biophys. J..

[29] Heimburg T., Angerstein B., Marsh D. (1999). Binding of Peripheral Proteins to Mixed Lipid Membranes: Effect of Lipid Demixing upon Binding. Biophys. J..

[30] Minton A.P. (1999). Adsorption of Globular Proteins on Locally Planar Surfaces. II. Models for the Effect of Multiple Adsorbate Conformations on Adsorption Equilibria and Kinetics. Biophys. J..

[31] Chatelier R.C., Minton A.P. (1996). Adsorption of Globular Proteins on Locally Planar Surfaces: Models for the Effect of Excluded Surface Area and Aggregation of Adsorbed Protein on Adsorption Equilibria. Biophys. J..

[32] Gorbenko G.P., Molotkovsky J.G., Kinnunen P.K.J. (2006). Cytochrome *c* Interaction with Cardiolipin/Phosphatidylcholine Model Membranes: Effect of Cardiolipin Protonation. Biophys. J..

[33] Heimburg T. (2007). Thermal Biophysics of Membranes.

[34] Helfand E., Frisch H.L., Lebowitz J.L. (1961). Theory of the Two- and One-Dimensional Rigid Sphere Fluids. J. Chem. Phys..

[35] Jähnig F. (1976). Electrostatic Free Energy and Shift of the Phase Transition for Charged Lipid Membranes. Biophys. Chem..

[36] Talbot J. (1997). Molecular Thermodynamics of Binary Mixture Adsorption: A Scaled Particle Theory Approach. J. Chem. Phys..

[37] Rytömaa M., Mustonen P., Kinnunen P.K.J. (1992). Reversible, Nonionic, and PH-Dependent Association of Cytochrome *c* with Cardiolipin-Phosphatidylcholine Liposomes. J. Biol. Chem..

[38] Rytömaa M., Kinnunen P.K.J. (1995). Reversibility of the Binding of Cytochrome *c* to Liposomes. Implications for Lipid-Protein Interactions. J. Biol. Chem..

[39] Rytömaa M., Kinnunen P.K.J. (1994). Evidence for Two Distinct Acidic Phospholipid-Binding Sites in Cytochrome *c*. J. Biol. Chem..

[40] Tuominen E.K.J., Wallace C.J.A., Kinnunen P.K.J. (2002). Phospholipid-Cytochrome *c* Interaction. J. Biol. Chem..

[41] Kalanxhi E., Wallace C.J.A. (2007). Cytochrome *c* Impaled: Investigation of the Extended Lipid Anchorage of a Soluble Protein to Mitochondrial Membrane Models. Biochem. J..

[42] Trusova V.M., Gorbenko G.P., Molotkovsky J.G., Kinnunen P.K.J. (2010). Cytochrome C-Lipid Interactions: New Insights from Resonance Energy Transfer. Biophys. J..

[43] Dragomir I., Hagarman A., Wallace C., Schweitzer-Stenner R. (2007). Optical Band Splitting and Electronic Perturbations of the Heme Chromophore in Cytochrome *c* at Room Temperature Probed by Visible Electronic Circular Dichroism Spectroscopy. Biophys. J..

[44] Schweitzer-Stenner R. (2008). Internal Electric Field in Cytochrome C Explored by Visible Electronic Circular Dichroism Spectroscopy. J. Phys. Chem. B.

[45] Schweitzer-Stenner R. (2024). Probing the Versatility of Cytochrome *c* by Spectroscopic Means: A Laudatio on Resonance Raman Spectroscopy. J. Inorg. Biochem..

[46] Pielak G.J., Oikawa K., Kay C.M., Mauk A.G., Smith M. (1986). Elimination of the Negative Soret Cotton Effect of Cytochrome c by Replacement of the Invariant Phenylalanine Using Site-Directed Mutagenesis. J. Am. Chem. Soc..

[47] Sinibaldi F., Fiorucci L., Patriarca A., Lauceri R., Ferri T., Coletta M., Santucci R. (2008). Insights into Cytochrome C-Cardiolipin Interaction. Role Played by Ionic Strength. Biochemistry.

[48] Pandiscia L.A., Schweitzer-Stenner R. (2015). Coexistence of Native-Like and Non-Native Cytochrome *c* on Anionic Lipsomes with Different Cardiolipin Content. J. Phys. Chem. B.

[49] Pandiscia L.A., Schweitzer-Stenner R. (2015). Coexistence of Native-like and Non-Native Partially Unfolded Ferricytochrome c on the Surface of Cardiolipin-Containing Liposomes. J. Phys. Chem. B.

[50] Steele H.B.B., Elmer-Dixon M.M., Rogan J.T., Ross J.B.A., Bowler B.E. (2020). The Human Cytochrome *c* Domain-Swapped Dimer Facilitates Tight Regulation of Intrinsic Apoptosis. Biochemistry.

[51] Rezaei Sani S.M., Akhavan M., Jalili S. (2018). Salt-Induced Effects on Natural and Inverse DPPC Lipid Membranes: Molecular Dynamics Simulation. Biophys. Chem..

[52] Kawai C., Pessoto F.S., Rodrigues T., Mugnol K.C.U., Tórtora V., Castro L., Milícchio V.A., Tersariol I.L.S., Di Mascio P., Radi R. (2009). PH-Sensitive Binding of Cytochrome *c* to the Inner Mitochondrial Membrane. Implications for the Participation of the Protein in Cell Respiration and Apoptosis. Biochemistry.

[53] Kawai C., Prado F.M., Nunes G.L.C., Di Mascio P., Carmona-Ribeiro A.M., Nantes I.L. (2005). PH-Dependent Interaction of Cytochrome *c* with Mitochondrial Mimetic Membranes: The Role of an Array of Positively Charged Amino Acids. J. Biol. Chem..

[54] Milorey B., Schweitzer-Stenner R., Kurbaj R., Malyshka D. (2019). PH-Induced Switch between Different Modes of Cytochrome *c* Binding to Cardiolipin-Containing Liposomes. ACS Omega.

[55] Milorey B., Malyshka D., Schweitzer-Stenner R. (2017). PH Dependence of Ferricytochrome *c* Conformational Transitions during Binding to Cardiolipin Membranes: Evidence for Histidine as the Distal Ligand at Neutral PH. J. Phys. Chem. Lett..

[56] O’Brien E.S., Nucci N.V., Fuglestad B., Tommos C., Wand A.J. (2015). Defining the Apoptopic Trigger: The Interaction of Cytochrome *c* and Cardiolipin. J. Biol. Chem..

[57] Elmer-Dixon M.M., Bowler B.E. (2017). Site A-Mediated Partial Unfolding of Cytochrome *c* on Cardiolipin Vesicles Is Species-Dependent and Does Not Require Lys72. Biochemistry.

[58] Rosell F.I., Ferrer J.C., Mauk A.G. (1998). Proton-Linked Protein Conformational Switching: Definition of the Alkaline Conformational Transition of Yeast Iso-1-Ferricytochrome c. J. Am. Chem. Soc..

[59] Döpner S., Hildebrandt P., Resell F.I., Mauk A.G. (1998). Alkaline Conformational Transitions of Ferricytochrome c Studied by Resonance Raman Spectroscopy. J. Am. Chem. Soc..

[60] Hagarman A., Duitch L., Schweitzer-Stenner R. (2008). The Conformational Manifold of Ferricytochromec Explored by Visible and Far-UV Electronic Circular Dichroism Spectroscopy. Biochemistry.

[61] Elmer-Dixon M.M., Bowler B.E. (2018). Electrostatic Constituents of the Interaction of Cardiolipin with Site A of Cytochrome c. Biochemistry.

[62] Elmer-Dixon M.M., Hoody J., Steele H.B.B., Becht D.C., Bowler B.E. (2019). Cardiolipin Preferentially Partitions to the Inner Leaflet of Mixed Lipid Large Unilamellar Vesicles. J. Phys. Chem. B.

[63] Elmer-Dixon M.M., Xie Z., Alverson J.B., Priestley N.D., Bowler B.E. (2020). Curvature-Dependent Binding of Cytochrome *c* to Cardiolipin. J. Am. Chem. Soc..

[64] Basova L.V., Kurnikov I.V., Wang L., Ritov V.B., Belikova N.A., Vlasova I.I., Pacheco A.A., Winnica D.E., Peterson J., Bayir H. (2007). Cardiolipin Switch in Mitochondria: Sutting off the Reduction of Cytochrome and Turning on the Peroxidase Activity. Biochemistry.

[65] Kapralov A.A., Kurnikov I.V., Vlasova I.I., Belikova N.A., Tyurin V.A., Basova L.V., Zhao Q., Tyurina Y.Y., Jiang J., Bayir H. (2007). The Hierarchy of Structural Transitions Induced in Cytochrome *c* by Anionic Phospholipids Determines Its Peroxidase Activation and Selective Peroxidation during Apoptosis in Cells. Biochemistry.

[66] Kapralov A.A., Yanamala N., Tyurina Y.Y., Castro L., Samhan-Arias A., Vladimirov Y.A., Maeda A., Weitz A.A., Peterson J., Mylnikov D. (2011). Topography of Tyrosine Residues and Their Involvement in Peroxidation of Polyunsaturated Cardiolipin in Cytochrome c/Cardiolipin Peroxidase Complexes. Biochim. Biophys. Acta Biomembr..

[67] McClelland L.J., Steele H.B.B., Whitby F.G., Mou T.-C., Holley D., Ross J.B.A., Sprang S.R., Bowler B.E. (2016). Cytochrome *c* Can Form a Well-Defined Binding Pocket for Hydrocarbons. J. Am. Chem. Soc..

[68] Englander S.W., Mayne L. (2017). The Case for Defined Protein Folding Pathways. Proc. Natl. Acad. Sci. USA.

[69] Hong Y., Muenzner J., Grimm S.K., Pletneva E.V. (2012). Origin of the Conformational Heterogeneity of Cardiolipin-Bound Cytochrome *c*. J. Am. Chem. Soc..

[70] Hanske J., Toffey J.R., Morenz A.M., Bonilla A.J., Schiavoni K.H., Pletneva E.V. (2012). Conformational Properties of Cardiolipin-Bound Cytochrome *c*. Proc. Natl. Acad. Sci. USA.

[71] Muenzner J., Toffey J.R., Hong Y., Pletneva E.V. (2013). Becoming a Peroxidase: Cardiolipin-Induced Unfolding of Cytochrome *c*. J. Phys. Chem. B.

[72] Malyshka D., Schweitzer-Stenner R. (2017). Ferrocyanide-Mediated Photoreduction of Ferricytochrome C Utilized to Selectively Probe Non-Native Conformations Induced by Binding to Cardiolipin-Containing Liposomes. Chem. Eur. J..

[73] Milazzo L., Tognaccini L., Howes B.D., Sinibaldi F., Piro M.C., Fittipaldi M., Baratto M.C., Pogni R., Santucci R., Smulevich G. (2017). Unravelling the Non-Native Low-Spin State of the Cytochrome c-Cardiolipin Complex: Evidence of the Formation of a His-Ligated Species Only. Biochemistry.

[74] Mandal A., Hoop C.L., Delucia M., Kodali R., Kagan V.E., Ahn J., Van Der Wel P.C.A. (2015). Structural Changes and Proapoptotic Peroxidase Activity of Cardiolipin-Bound Mitochondrial Cytochrome *c*. Biophys. J..

[75] Li M., Sun W., Tyurin V.A., DeLucia M., Ahn J., Kagan V.E., van der Wel P.C.A. (2021). Activation of Cytochrome C Peroxidase Function Through Coordinated Foldon Loop Dynamics upon Interaction with Anionic Lipids. J. Mol. Biol..

[76] Hu W., Kan Z.-Y., Mayne L., Englander S.W. (2016). Cytochrome *c* Folds through Foldon-Dependent Native-like Intermediates in an Ordered Pathway. Proc. Nat. Acad. Sci. USA.

[77] Zhan J., Zeng D., Xiao X., Fang Z., Huang T., Zhao B., Zhu Q., Liu C., Jiang B., Zhou X. (2024). Real-Time Observation of Conformational Changes and Translocation of Endogenous Cytochrome *c* within Intact Mitochondria. J. Am. Chem. Soc..

[78] Paul M., Govind C., Karunakaran V. (2024). Significance of the Double Bond in the Acyl Chain of Cardiolipin Revealed by the Partial Unfolding Dynamics of Cytochrome *c* Using Femtosecond Transient Absorption Spectroscopy. J. Phys. Chem. B.

[79] Hildebrandt P., Stockburger M. (1989). Cytochrome *c* at Charged Interfaces. 2. Complexes with Negatively Charged Macromolecular Systems Studied by Resonance Raman Spectroscopy. Biochemistry.

[80] Wackerbarth H., Hildebrandt P. (2003). Redox and Conformational Equilibria and Dynamics of Cytochrome *c* at High Electric Fields. ChemPhysChem.

[81] Hildebrandt P., Heimburg T., Marsh D. (1990). Quantitative Conformational Analysis of Cytochrome *c* Bound to Phospholipid Vesicles Studied by Resonance Raman Spectroscopy. Eur. Biophys. J..

[82] Oellerich S., Lecomte S., Paternostre M., Heimburg T., Hildebrandt P. (2004). Peripheral and Integral Binding of Cytochrome *c* to Phospholipids Vesicles. J. Phys. Chem. B..

[83] De Kruijff B., Cullis P.R. (1980). Cytochrome *c* Specifically Induces Non-Bilayer Structures in Cardiolipin Containing Model Membranes. Biochim. Biophys. Acta.

[84] Mohammadyani D., Yanamala N., Samhan-Arias A.K., Kapralov A.A., Stepanov G., Nuar N., Planas-Iglesias J., Sanghera N., Kagan V.E., Klein-Seetharaman J. (2018). Structural Characterization of Cardiolipin-Driven Activation of Cytochrome *c* into a Peroxidase and Membrane Perturbation. Biochim. Biophys. Acta (BBA)—Biomembr..

[85] Beales P.A., Bergstrom C.L., Geerts N., Groves J.T., Vanderlick T.K. (2011). Single Vesicle Observations of the Cardiolipin-Cytochrome *c* Interaction: Induction of Membrane Morphology Changes. Langmuir.

[86] Bergstrom C.L., Beales P.A., Lv Y., Vanderlick T.K., Groves J.T. (2013). Cytochrome *c* Causes Pore Formation in Cardiolipin-Containing Membranes. Proc. Natl. Acad. Sci. USA.

[87] Kitt J.P., Bryce D.A., Minteer S.D., Harris J.M. (2017). Raman Spectroscopy Reveals Selective Interactions of Cytochrome *c* with Cardiolipin That Correlate with Membrane Permeability. J. Am. Chem. Soc..

[88] Gajhede M., Schuller D., Henricksen A., Smith A., Poulos T. (1997). Crystal Structure Determination of Classical Horseradish Peroxidase at 2.15A Resolution. Nat. Struct. Mol. Biol..

[89] Kincaid J.R., Zheng Y., Al-Mustafa J., Czarnecki K. (1996). Resonance Raman Spectra of Native and Mesoheme-Reconstituted Horseradish Peroxidase and Their Catalytic Intermediates. J. Biol. Chem..

[90] Belikova N.A., Vladimirov Y.A., Osipov A.N., Kapralov A.A., Tyurin V.A., Potapovich M.V., Basova L.V., Peterson J., Kurnikov I.V., Kagan V.E. (2006). Peroxidase Activity and Structural Transitions of Cytochrome *c* Bound to Cardiolipin-Containing Membranes. Biochemistry.

[91] Patriarca A., Polticelli F., Piro M.C., Sinibaldi F., Mei G., Bari M., Santucci R., Fiorucci L. (2012). Conversion of Cytochrome *c* into a Peroxidase: Inhibitory Mechanisms and Implication for Neurodegenerative Diseases. Arch. Biochem. Biophys..

[92] Shah R., Schweitzer-Stenner R. (2008). Structural Changes of Horse Heart Ferricytochrome *c* Induced by Changes of Ionic Strength and Anion Binding. Biochemistry.

[93] Kurbaj R., Milorey B., Schweitzer-Stenner R. (2019). Substrate Induced Conformational Changes of Liposomes Bound Cytochrome *c*. Biophys. J..

[94] McClelland L.J., Mou T.-C., Jeakins-Cooley M.E., Sprang S.R., Bowler B.E. (2014). Structure of a Mitochondrial Cytochrome *c* Conformer Competent for Peroxidase Activity. Proc. Nat. Acad. Sci. USA.

[95] Kleckner I.R., Foster M.P. (2011). An Introduction to NMR-Based Approaches for Measuring Protein Dynamics. Biochim. Biophys. Acta (BBA)—Proteins Proteom..

[96] Olafsson G., Sparr E. (2013). Ionization Constants pKa of Cardiolipin. PLoS ONE.

[97] Kooijman E.E., Swim L.A., Garber Z.T., Tyurina Y.Y., Bayir H., Kagan V.R. (2017). Magic Angle Spinning ^31^NMR Spectroscopy Reveals Two Essentially Identical Ionization States for the Cardiolipin Phosphates in Phospholipid Liposomes. Biochim. Biophys. Acta.

[98] Schwalbe M., Ozenne V., Bibow S., Jaremko M., Jaremko L., Gajda M., Jensen M.R., Biernat J., Becker S., Mandelkow E. (2014). Predictive Atomic Resolution Descriptions of Intrinsically Disordered HTau40 and α-Synuclein in Solution from NMR and Small Angle Scattering. Structure.

[99] Schweitzer-Stenner R. (2023). The Relevance of Short Peptides for an Understanding of Unfolded and Intrinsically Disordered Proteins. Phys. Chem. Chem. Phys..

[100] Tanaka S., Scheraga H.A. (1976). Statistical Mechanical Treatment of Protein Conformation. 4. A Four-State Model for Specific-Sequence Copolymers of Amino Acids. Macromolecules.

[101] Tanaka S., Scheraga H.A. (1977). Statistical Mechanical Treatment of Protein Conformation. 5. A Multistate Model for Specific Sequence Copolymers of Amino Acids. Macromolecules.

[102] Khare S.D., Chinchilla P., Baum J. (2023). Multifaceted Interactions Mediated by Intrinsically Disordered Regions Play Key Roles in Alpha Synuclein Aggregation. Curr. Opin. Struct. Biol..

[103] Davidson W.S., Jonas A., Clayton D.F., George J.M. (1998). Stabilization of α-Synuclein Secondary Structure upon Binding to Synthetic Membranes. J. Biol. Chem..

[104] Andersson A., Linse S., Sparr E., Fornasier M., Jönsson P. (2024). The Density of Anionic Lipids Modulates the Adsorption of α-Synuclein onto Lipid Membranes. Biophys. Chem..

[105] Busch D.J., Houser J.R., Hayden C.C., Sherman M.B., Lafer E.M., Stachowiak J.C. (2015). Intrinsically Disordered Proteins Drive Membrane Curvature. Nat. Commun..

[106] Hellstrand E., Grey M., Ainalem M.-L., Ankner J., Forsyth V.T., Fragneto G., Haertlein M., Dauvergne M.-T., Nilsson H., Brundin P. (2013). Adsorption of α-Synuclein to Supported Lipid Bilayers: Positioning and Role of Electrostatics. ACS Chem. Neurosci..

[107] Makasewicz K., Wennmalm S., Stenqvist B., Fornasier M., Andersson A., Jönsson P., Linse S., Sparr E. (2021). Cooperativity of α-Synuclein Binding to Lipid Membranes. ACS Chem. Neurosci..

[108] Adair G.S., Bock A.V., Field H. (1925). THE HEMOGLOBIN SYSTEM. J. Biol. Chem..

[109] Monod J., Wyman J., Changeux J.-P. (1965). On the Nature of Allosteric Transitions: A Plausible Model. J. Mol. Biol..

[110] Braun A.R., Lacy M.M., Ducas V.C., Rhoades E., Sachs J.N. (2017). α-Synuclein’s Uniquely Long Amphipathic Helix Enhances Its Membrane Binding and Remodeling Capacity. J. Membr. Biol..

[111] Viennet T., Wördehoff M.M., Uluca B., Poojari C., Shaykhalishahi H., Willbold D., Strodel B., Heise H., Buell A.K., Hoyer W. (2018). Structural Insights from Lipid-Bilayer Nanodiscs Link α-Synuclein Membrane-Binding Modes to Amyloid Fibril Formation. Commun. Biol..

[112] Denisov I.G., Sligar S.G. (2016). Nanodiscs for Structural and Functional Studies of Membrane Proteins. Nat. Struct. Mol. Biol..

[113] Pirc K., Ulrih N.P. (2015). α-Synuclein Interactions with Phospholipid Model Membranes: Key Roles for Electrostatic Interactions and Lipid-Bilayer Structure. Biochim. Biophys. Acta (BBA)—Biomembr..

[114] Middleton E.R., Rhoades E. (2010). Effects of Curvature and Composition on α-Synuclein Binding to Lipid Vesicles. Biophys. J..

[115] Rhoades E., Ramlall T.F., Webb W.W., Eliezer D. (2006). Quantification of α-Synuclein Binding to Lipid Vesicles Using Fluorescence Correlation Spectroscopy. Biophys. J..

[116] Rocha S., Kumar R., Nordén B., Wittung-Stafshede P. (2021). Orientation of α-Synuclein at Negatively Charged Lipid Vesicles: Linear Dichroism Reveals Time-Dependent Changes in Helix Binding Mode. J. Am. Chem. Soc..

[117] Bernadó P., Bertoncini C.W., Griesinger C., Zweckstetter M., Blackledge M. (2005). Defining Long-Range Order and Local Disorder in Native α-Synuclein Using Residual Dipolar Couplings. J. Am. Chem. Soc..

[118] Li B., Ge P., Murray K.A., Sheth P., Zhang M., Nair G., Sawaya M.R., Shin W.S., Boyer D.R., Ye S. (2018). Cryo-EM of Full-Length α-Synuclein Reveals Fibril Polymorphs with a Common Structural Kernel. Nat. Commun..

[119] Nelson R., Sawaya M.R., Balbirnie M., Madsen A., Riekel C., Grothe R., Eisenberg D. (2005). Structure of the Cross-β Spine of Amyloid-like Fibrils. Nature.

[120] Schweitzer-Stenner R. (2024). The Physics of Protein Structure and Dynamics. When and Why Proteins Fold and Don’t Fold.

[121] Haque F., Pandey A.P., Cambrea L.R., Rochet J.-C., Hovis J.S. (2010). Adsorption of α-Synuclein on Lipid Bilayers: Modulating the Structure and Stability of Protein Assemblies. J. Phys. Chem. B.

[122] Galvagnion C., Buell A.K., Meisl G., Michaels T.C.T., Vendruscolo M., Knowles T.P.J., Dobson C.M. (2015). Lipid Vesicles Trigger α-Synuclein Aggregation by Stimulating Primary Nucleation. Nat. Chem. Biol..

[123] Gaspar R., Lund M., Sparr E., Linse S. (2020). Anomalous Salt Dependence Reveals an Interplay of Attractive and Repulsive Electrostatic Interactions in α-Synuclein Fibril Formation. QRB Discov..

[124] Kiskis J., Horvath I., Wittung-Stafshede P., Rocha S. (2017). Unraveling Amyloid Formation Paths of Parkinson’s Disease Protein α-Synuclein Triggered by Anionic Vesicles. Q. Rev. Biophys..

[125] Ghosh S., Mahapatra A., Chattopadhyay K. (2019). Modulation of α-Synuclein Aggregation by Cytochrome *c* Binding and Hetero-Dityrosine Adduct Formation. ACS Chem. Neurosci..

[126] Sinibaldi F., Howes B.D., Piro M.C., Polticelli F., Bombelli C., Ferri T., Coletta M., Smulevich G., Santucci R. (2010). Extended Cardiolipin Anchorage to Cytochrome *c*: A Model for Protein-Mitochondrial Membrane Binding. J. Biol. Inorg. Chem..

